# Executive summary of the European consensus report on the diagnosis and treatment of monoclonal gammopathy of renal significance

**DOI:** 10.1093/ckj/sfag163

**Published:** 2026-05-26

**Authors:** Ben Sprangers, Camille Cohen, Viviane Gnemmi, Amir Shabaka, Gema Fernandez-Juarez, Dominique van Midden, Eric Steenbergen, Paolo Milani, Marguerite Vignon, Benjamin Wilde, Ute Hegenbart, Jack F Wetzels

**Affiliations:** Department of Nephrology, Ziekenhuis Oost-Limburg, Genk, Belgium, and Department of Immunology and Infection, Biomedical Research Institute, UHasselt, Diepenbeek, Belgium; Department of Nephrology, APHP, Hôpital Bichat, Paris, France; Center for Inflammation Research (CRI), INSERM U1149, University de Paris, Paris, France; Department of Pathology, Lille, France; Hospital Universitario La Paz. IdiPAz, Madrid, Spain; Hospital Universitario La Paz. IdiPAz, Madrid, Spain; Department of Pathology, Radboudumc Nijmegen, The Netherlands; Department of Pathology, Radboudumc Nijmegen, The Netherlands; Department of Molecular Medicine, University of Pavia, Pavia, Italy; Amyloidosis Research and Treatment Center, Foundation “Istituto di Ricovero e Cura a Carattere Scientifico (IRCCS) Policlinico San Matteo”, Italy; Hopital Cochin, APHP, Paris, France; Department of Nephrology, University Hospital Essen, University Duisburg-Essen, Essen, Germany; University Hospital, Amyloidosis Center, HeidelbergGermany; Department of Nephrology, Radboud University Medical Center, Nijmegen, The Netherlands

**Keywords:** AL amyloidosis, kidney transplantation, MGRS, monoclonal gammopathy, monoclonal immunoglobulin deposition disease

## Abstract

We introduce the European Union consensus report on the diagnosis and treatment of monoclonal gammopathy of renal significance (MGRS), a generic term to describe kidney disorders caused by a non-malignant monoclonal immunoglobulin clone. There are many subtypes of MGRS, with AL-amyloidosis being most prevalent. The consensus report aims at providing guidance to clinicians, pathologists and laboratory specialists who take care of patients with a (suspected) diagnosis of MGRS. This executive summary provides a condensed overview of the most important aspects of the diagnosis and management of patients with MGRS. Consultation of expert centers is strongly advised. Although most patients will benefit from hematological, clone-directed therapy, treatment decisions must be individualized, in view of the heterogeneity of patients between and within the MGRS subtypes. The final chapter discusses kidney transplantation in patients with MGRS. The working group acknowledges that most advice is based on low-quality evidence. The full report provides the rationale and a detailed discussion of the available literature. This executive summary is an introduction, and not a substitute for the full consensus report.

## INTRODUCTION

Monoclonal gammopathy of renal significance (MGRS) is defined by kidney damage caused by (or attributed to) a monoclonal immunoglobulin (MIg), in the absence of a B cell or plasma cell clone that meets the criteria for specific hematological therapy. MGRS was only recently recognized as disease entity, and all subtypes of MGRS are (very) rare. The European Rare Kidney Disease network (ERKnet) identified MGRS as a (group of) diseases that lacked clinical practice guidelines. A multidisciplinary working group has addressed this need. The working group was composed of nephrologists, hematologists and renal pathologists. In MGRS, making a proper diagnosis is of the utmost importance. Furthermore, in these rare diseases there are no or very few randomized therapeutic trials. Therefore, it is impossible to make evidence-based recommendations. Since clinical practice guidance is often based on expert opinion, and will vary between countries and continents, the working group was limited to members of countries participating in ERKnet, and the consensus report is written from a European perspective. The members of the working group identified relevant literature and provided expert opinion. All chapters were discussed extensively, and the consensus report provides the conclusions.

The full consensus report (available in the Supplementary Appendix) consists of 10 chapters. Each chapter provides a summary of advisory statements, followed by a brief rationale, and extensive supportive evidence containing relevant literature references. Chapter 1 provides an overview and introduction of MGRS, Chapter 10 summarizes aspects of kidney transplantation in patients with MGRS and Chapters 2–9 discuss the most important subtypes of MGRS (Table 1).

**Table 1:** Subtypes of MGRS, discussed in specific chapters of the consensus report.

Chapter 2: MIg-associated amyloidosis
Chapter 3: MIg deposition disease (MIDD)
Chapter 4: Cryoglobulinemia
Chapter 5: Proliferative glomerulonephritis with monoclonal immune deposits (PGNMID)
Chapter 6: Immunotactoid glomerulopathy (ITG)
Chapter 7: Fibrillary glomerulopathy with monoclonal immune deposits
Chapter 8: Light chain proximal tubulopathy and light chain crystalline podocytopathy
Chapter 9: C3 glomerulopathy

The consensus report emphasizes that most patients will benefit from referral to (or consultation of) an expert center. Diagnosis and management of a patient with MGRS requires multidisciplinary collaboration. We underline that management of patients with MGRS should be individualized, therefore the consensus report should not be interpreted as a manual for patient management. Rather we intend to provide relevant and useful information that will guide the clinician on when to suspect a diagnosis of MGRS, and how to properly diagnose (or exclude a diagnosis of) MGRS and evaluate a patient with MGRS. We also introduce general principles of treatment.

This executive summary is an introduction to MGRS and should enable clinicians to quickly gain insight in MGRS, to identify the pitfalls in the diagnosis of MGRS and to understand the complexity of management decisions in the individual patient. This executive summary is not intended to be complete, is not fully referenced and cannot replace the full consensus report. Since MGRS are rare diseases, with limited data and low levels of evidence, the full consensus report should be accessed for detailed information and an extensive discussion of the rationale and supportive literature. In this summary, we specifically highlight Chapters 1, 2 and 10, and briefly quote the most relevant information from Chapters 3–9.

## CHAPTER 1: MONOCLONAL GAMMOPATHY OF RENAL SIGNIFICANCE: DEFINITION, DIAGNOSIS AND GENERAL ASPECTS OF TREATMENT [1–8]

MGRS are rare diseases. The overall MGRS incidence rate is estimated at 10–20 per million patient-years, with AL-amyloidosis being most frequent, and comprising 40%–70% of MGRS cases. With the introduction of effective hematological therapy MGRS has become a treatable disease. Therefore, it is important to diagnose MGRS in an early stage, i.e. before reaching the point of no return. On the other hand, chronic kidney disease (CKD) and monoclonal gammopathy of undetermined significance (MGUS) are frequent, especially in an elderly population. An optimal diagnostic pathway is needed that avoids missing a diagnosis of MGRS while limiting unnecessary diagnostic tests and procedures. The consensus report argued against the routine measurement of M-protein and free light chains (FLC) in patients with CKD and against a routine kidney biopsy in patients with MGUS. There is no evidence that such routine tests improve diagnosis and management. Screening in patients with CKD of serum with electrophoresis (SPEP) and immunofixation (SIFE) has limited positive predictive value. Moreover, sensitivity is relatively low, thus screening cannot be used to rule out MGRS. There are also no good data to support the FLC assay as screening tool; moreover, this test adds considerable costs. Therefore, performing a kidney biopsy without prior screening for an MIg is a cost-effective approach and clinical criteria can be used to guide decisions regarding kidney biopsy. Suggested criteria are new-onset proteinuria >1 g/day, progressive kidney disease [deterioration of estimated glomerular filtration rate (eGFR) or increasing proteinuria] or the presence of (suspected) extrarenal manifestations. These criteria should also be used to guide a decision toward kidney biopsy in patients with known MGUS. Importantly, in patients with CKD in whom the decision to perform a biopsy is equivocal, and no alternative diagnosis for kidney disease is likely, evaluation of serum for the presence of an M-protein, and measurement of FLC might help in decision-making. Of note, guidelines published by the British Society of Haematology and the International Kidney and Monoclonal Gammopathy working group suggest otherwise. We refer to the full consensus report that discusses the supportive evidence. Country-specific guidelines may prefer routine screening for an MIg in patients with kidney injury, and many centers prefer such screening in patients who have a planned kidney biopsy, to guide the pathologist in evaluating the kidney biopsy [performing Congo red, electron microscopy (EM), paraffin immunofluorescence (IF)]. This might be a rational approach in unexperienced centers or centers that use a limited kidney biopsy evaluation. In patients with nephrotic syndrome, presenting with manifestations that are suggestive of amyloidosis MIg testing (by serum and urine IFE and serum FLC assay) may guide the evaluation of the patient by prioritizing a fat biopsy to document AL amyloid deposition.

In the follow-up of patients with MGUS and limited kidney injury, we caution against the use of urine albumin creatinine ratio (UACR) as sole measure of proteinuria, since this will not enable detection of increasing low molecular weight or light chain proteinuria. Therefore, measurement of urine protein creatinine ratio (UPCR) is preferred. The ratio UACR/UPCR can be used to guide requests for additional urine evaluation.

Proper histopathological evaluation of a kidney biopsy is key in diagnosing MGRS. It is important to realize that the absence of an MIg in serum or urine does not exclude MGRS. Therefore, each kidney biopsy should be properly evaluated, which includes the use of appropriate staining for light microscopy, and IF, if necessary IF on paraffin-embedded tissue after pronase/proteinase digestion, and preferably evaluation of the biopsy by EM (Table 2). In selected cases, especially in patients with amyloidosis, additional techniques may be required to firmly establish the diagnosis and amyloid subtype. Characteristics diagnostic features of various MGRS subtypes are listed in Table 3. An updated classification was recently proposed by a working group of the Renal Pathology Society and the International Kidney and Monoclonal Gammopathy Research group (see full consensus report). Admittedly, not all techniques are (easily) available. Therefore, the full consensus report provides some guidance to allow diagnosis of MGRS without using these techniques.

**Table 2:** Kidney biopsy requirements.

Light microscopy	Staining: methenamine silver, PAS, hematoxylin and eosin, trichrome
	On indication: Congo red, DNAJB9
IF on frozen tissue	Minimal staining: IgG, IgM, IgA, к, λ, C3, C1q
	On indication: IgG subclasses; amyloid protein subtypes (see Chapter 2)
Immunohistochemistry on paraffin	Staining as in IF
On indication: IF after pronase digestion	• Fresh tissue unavailable
	• Suspected LCPT, LCCP or crystalglobulin nephropathy
	• Necessary for the evaluation of C3G or MPGN with negative IF in laboratories that use transport medium for kidney biopsies
	• Membranous nephropathy with negative staining for IgG
Electronmicroscopy	• Advised in all MGRS
	• Essential for the diagnosis of ITG and DDD
	• Relevant for the detection of organized deposits
	• Advantageous for confirmation of fibrils and localization of non-organized deposits

**Table 3:** Pathognomonic features in MGRS subtypes.^a^

MGRS subtype	LM	IF	EM
Amyloidosis	Congo red positive		Fibrils 7–12 nm
MIDD		Linear staining of TBM	Powdery, punctate deposits
Cryoglobulinemia	Hyaline thrombi		Annular, microtubular, or cylindrical substructure
PGNMID			
ITG			Microtubules
FGN	DNAJB9 positive		Fibrils
LCPT/LCCP	Crystalline structure in proximal tubule or podocyte	Routine IF negative; use pronase digestion	Crystals, amorphous deposits within lysosomes
C3G		C3 dominant staining	

a Typically, in all but one (C3G) subtype of MGRS staining in IF shows light chain restriction, heavy chain restriction or monotypic IgG subclass staining. FGN, fibrillary glomerulonephritis; LM, light microscopy.

In patients with biopsy-proven MGRS, the culprit MIg should be searched for in serum or urine [9–11]. We advise initial screening of serum for the presence of an MIg using SPEP/SIFE. It is important to be aware that these techniques are not very sensitive, thus small amounts of an MIg may escape detection (<1 g/L). Additionally, serum should be used to measure FLC, an assay that has allowed the detection of subtle hematological abnormalities. Urine evaluation with IFE is advised in patients with negative/normal serum findings. Finding an MIg in serum or urine is important, often confirming the diagnosis in patients with biopsy-proven MGRS, establishing a diagnosis of MGRS in patients with C3G, and providing a biomarker to evaluate hematological treatment response. Of note, when measuring FLC it is important to know that FLC levels are dependent on eGFR. Therefore, in patients with kidney failure the к/λ ratio is different from normal controls (Table 4). Unfortunately, the originally proposed reference intervals are debated, due to an upward drift of the κ Freelite assay. Moreover, there are differences between assays. Although data are limited, clinicians should be aware that these assays cannot be used interchangeably. The same assay should be used consistently in the follow-up of patients. Hematological response to therapy is to some extent based on changes in the measured FLC. Response criteria not only evaluate the κ/λ ratio, but also the difference between the involved and uninvolved light chain (dFLC). Since kidney failure will not only affect the absolute levels but also the FLC ratio and the dFLC, interpretation of response criteria is hampered. In general, hematological response will be underestimated in patients with CKD stage 4 and 5.

**Table 4:** Proposed reference values for к/λ ratio in CKD^a^.

	Lower limit normal	Upper limit normal
eGFR 45–60 mL/min/1.73 m^2^	0.46	2.62
eGFR 30–45 mL/min/1.73 m^2^	0.48	3.38
eGFR <30 mL/min/1.73 m^2^	0.54	3.30

a Values based on FLC measurement using Freelite^®^ assay. The original reference interval was 0.26–1.65. Due to technical issues of the calibrator there has been an upward drift of the κ levels, and new reference values should be implemented. Revised intervals have been proposed: FLC ratio ranging from 0.44–2.16 in <70-year-old persons and 0.46–2.59 in those aged ≥70 years (see references [9] and [10]).

In patients with MGRS, bone marrow (BM) studies are needed to identify the causative clone. It is debated whether BM evaluation is necessary in patients with negative SPEP/SIFE and normal FLC—it is very unlikely to find BM abnormalities in such patients. Still, we suggest performing BM examination, including flow cytometry, in all patients with MGRS before starting therapy. This recommendation is based on the fact that MGRS are rare diseases, and that all efforts should be made for thorough evaluation of the patient. Depending on the nature of the underlying clone and the isotype of the MIg, performance of additional radiological, molecular or cytogenetic studies should be considered, according to hematological guidelines.

**Figure 1:**
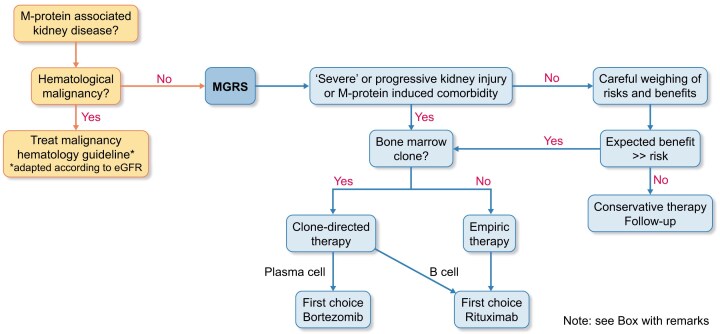
Overall algorithm of management of patients with non-AL-amyloid MGRS.

Chapter 1 discusses general aspects of treatment. Although evidence in the MGRS population is lacking, we suggest that patients with MGRS should receive therapy as proposed in the KDIGO CKD guidelines. This includes targeting low blood pressures, use of angiotensin-converting enzyme inhibitor (ACEi) or angiotensin-receptor blocker (ARB), statin therapy and possibly novel anti-proteinuric therapies. Obviously, conservative treatment should be guided by patient characteristics: as an example, patients with amyloidosis often have low blood pressure, and in these patients ACEi or ARB should be used carefully. In addition, quality of life and life expectancy should be considered. Many patients with MGRS will benefit from hematological therapy. Treatment of patients with AL-amyloidosis is based on evidence from randomized controlled trials (RCTs) (see discussion in Chapter 2). There is no evidence from RCTs to guide management in patients with other MGRS subtypes. Treatment decisions require balancing benefits and risks, adapting treatment to the needs and expectations of the patient. The decision to start therapy and the choice of therapy is dependent on patient characteristics, the underlying disease, the course of proteinuria and eGFR, and patient and physician preference. A “wait and see” policy may be preferred in patients with MGRS who are older, have clinically relevant comorbidities, or low performance status and low life expectancy. Since few patients will die from a hematological malignancy, the goal of treatment is to prevent extrarenal and renal complications. The approach towards treatment is different for the types of MGRS that are associated with a risk for extrarenal (cardiac) disease. In these subtypes a complete or very good partial hematological response is necessary to prevent progressive heart and kidney failure. In contrast, in patients with renal-limited MGRS, where renal outcome is the only relevant parameter, even a partial response to therapy may be sufficient to stabilize eGFR and reduce proteinuria. In general, renal response follows the hematological response. However, in patients with low eGFR or severe proteinuria at start of therapy, kidney disease will often progress despite a hematological response. In such cases treatment may be futile. Since in MGRS the MIg is responsible for causing kidney injury, it is logical to use treatment that targets the underlying clone. An overarching algorithm of management of patients with non-AL-amyloid MGRS and an overview of the relevant questions that guide decision-making are provided in Fig. 1. The management of patients who are prepared for kidney transplantation deserves special attention. These patients are discussed in Chapter 10.

## CHAPTER 2: MONOCLONAL IMMUNOGLOBULIN–ASSOCIATED AMYLOIDOSIS [12–15]

In amyloidosis organ damage is attributed to fibrillary deposits. These fibrils are aggregates of misfolded proteins or degraded protein fragments. Amyloidosis is diagnosed in a biopsy showing pale pink, amorphous, silver-negative and acellular deposits in light microscopy, which are positive in Congo red staining, with the typical apple-green birefringence under polarized light. On EM, amyloid deposits show randomly arranged fibrils of 7–12 nm diameter.

There are >40 different amyloidogenic proteins. Interestingly, these various amyloid subtypes have different organ distribution. Kidney involvement is mainly limited to the forms of amyloidosis listed in Table 5. MIg-associated amyloidosis is the major subtype. In MIg-associated amyloidosis, the amyloid precursor protein can be derived from a light chain (AL-amyloidosis), a heavy chain (AH-amyloidosis) or both (AL/AH-amyloidosis). AL-amyloidosis is most frequent. Although a hematological malignancy can be present, most patients with amyloidosis can be considered part of the spectrum of MGRS. AL-amyloidosis typically is a systemic disease with involvement of many organs and tissue, which explains the large variation in clinical symptoms (Table 6). Some clinical features are typically seen in AL-amyloidosis (macroglossia, peri-orbital hematoma, factor X deficiency).

**Table 5:** Precursor proteins in renal amyloidosis.

Protein	Amyloid nomenclature	Hereditary/acquired
Immunoglobulins		
Light chain	AL-amyloidosis	A
Heavy chain	AH-amyloidosis	A
Light and heavy chains	AL/AH-amyloidosis	A
Amyloid-A protein	AA-amyloidosis	A/H^b^
Leukocyte chemotactic factor 2	ALECT2 amyloidosis	A
Fibrogen α	AFib amyloidosis	H
Lysozyme	ALys amyloidosis	H
Apolipoprotein AI	AApoAI amyloidosis	H
Apolipoprotein AII	AApoAII amyloidosis	H
Apolipoprotein AIV	AApoAIV amyloidosis	A/H
Apolipoprotein CII	AApoCII amyloidosis	H
Apolipoprotein CIII	AApoCIII amyloidosis	H
Gelsolin	AGel amyloidosis	H
Transthyretin^a^	ATTR amyloidosis	H,A
(Pro)calcitonin	ACal amyloidosis	A^c^
Il-1 receptor antagonist protein	AIL1RAP amyloidosis	A^d^

A, amyloid; L, light chain; H, heavy chain; L/H, light/heavy chain; AA, seum amyloid A protein; LEC2, leukocyte chemotactic factor 2; FIb, Fibrogen alpha; Lys, lysozyme; Apo, apolipoprotein; Gel, Gelsolin; TTR, transthyretrin; Cal, (pro)calcitonin; RAP, receptor antagonist protein.

a In ATTR-amyloidosis kidney involvement is very rare; it can be observed in the hereditary form of ATTR amyloidosis, but is unlikely as the sole manifestation.

b Most hereditary forms of AA-amyloidosis concern genetic autoinflammatory diseases.

c Associated with a tumor that produces (pro)calcitonin.

d Iatrogenic, caused by administration of anakinra.

**Table 6:** Organ involvement in AL-amyloidosis.

**Heart**
Edema, fatigue, hypotension
Heart failure with preserved ejection fraction
Low voltage ECG
Thickened ventricular wall (ultrasound), increased myocardial extracellular volume and late gadolinium enhancement (MRI)
Less negative left ventricular global longitudinal strain
Increased NTproBNP (or BNP)
**Nervous system**
Peripheral nervous system: sensorimotor polyneuropathy
Carpal tunnel syndrome
Autonomic: postural hypotension, erectile dysfunction, intestinal motility disorders
Gastrointestinal tract
Malabsorption, diarrhea, weight loss, constipation
**Liver**
Increased alkaline phosphatase
Hepatomegaly
Increased bilirubin
**Other**
Periorbital purpura
Macroglossia
Factor X deficiency
Soft tissue involvement

ECG, electrocardiogram; MRI, magnetic resonance imaging.

It is very important to correctly identify the subtype of amyloidosis. Notably, the presence of an MIg in serum or urine in a patient with Congo red–positive deposits is not sufficient to make a diagnosis of MIg-associated amyloidosis. The involved protein should be typed by the use of IF and/or immunohistochemistry studies. Thus, a diagnosis of amyloidosis should be supported by two techniques, e.g. the identification of a monoclonal light chain as constituent of the fibrils with detection of the involved FLC in serum or urine. Additional studies may be necessary to definitively rule out other types/causes of amyloidosis. Mass spectrometry is recommended when IF, immunohistochemistry (using appropriate antibodies) or immune-electronmicroscopy fail to identify the protein involved in the amyloid deposits in a patient with suspected MIg-amyloidosis. Unfortunately, this technique is costly, and not always available. In such patients a diagnosis of MIg-amyloidosis is highly likely in the presence of an MIg in serum or urine, and after exclusion of other relevant subtypes (e.g. negative staining for AA-amyloid or LECT2, and negative genetic testing for the hereditary forms).

Since AL-amyloidosis is a systemic disease, the diagnosis of amyloidosis should be suspected in a patient with CKD and evidence of extra-renal organ damage. We suggest that evaluation of a patient for extrarenal damage using easily available cardiac biomarkers might be helpful when in doubt.

In some patients, kidney injury is the only clinical sign. Since kidney response to therapy is more likely in patients who start therapy before the onset of nephrotic syndrome or CKD stage 3b, an early diagnosis is needed. Since the prevalence of CKD and MGUS in an elderly population is high, a practical approach to guide the decision to perform a kidney biopsy is suggested. The consensus report suggests considering using the ratio of 24-h proteinuria (in mg/day) to eGFR (in mL/min/1.73 m^2^).

All patients with AL-amyloidosis should routinely be tested using cardiac biomarkers (NTproBNP or BNP and troponin) and cardiac ultrasound. Cardiac magnetic resonance imaging is the most sensitive, and should be considered when available in selected patients. Other extrarenal manifestations must also be carefully evaluated.

Patients with amyloidosis and severe kidney injury (proteinuria >5 g/day or eGFR <50 mL/min/1.73 m^2^) are at very high risk of developing end-stage kidney disease (ESKD). However, patients with amyloidosis and cardiac involvement are at the highest risk, and experience a very high early mortality. AL-amyloidosis thus constitutes an emergency, and an accurate diagnosis must be made quickly, to allow early start of therapy. A rapid reduction (within 4–12 weeks) of the culprit light chain is the goal of therapy, and is associated with improved outcome. Therefore, it is important to evaluate response at an early timepoint, and assessment at 1 month after start of therapy is suggested.

In patients with kidney disease, there is an association between hematological response and renal response. Thus, renal response is observed only in patients with hematological complete or very good partial response. Hematological response criteria are used based on a regular evaluation of the MIg, and the FLC concentration (Table 7). Still, many patients who present with nephrotic syndrome and/or eGFR <50 mL/min/1.73 m^2^ will progress to kidney failure, even when there is a good hematological response. As expected, renal outcome not only is dependent on kidney injury parameters at baseline, but also is associated with renal response. Renal response criteria are given in Table 7.

**Table 7:** Hematological and renal response criteria.

**Hematological response**	
Complete response:	Two criteria must be met:absence of amyloidogenic monoclonal protein defined by negative immunofixation electrophoresis of serum and urineANDFLC ratio within reference range or uninvolved FLC concentration is greater than involved FLC concentration (with or without normal FLC ratio)
Very good partial response:^a^	dFLC <40 mg/dL
Partial response	50% reduction in dFLC
**Renal response**	
Complete response^b^	Proteinuria <0.2 g/day
Very good partial response^b^	>60% reduction in proteinuria
Partial response^b^	>30% and ≤60% reduction in proteinuria
No response	≤30% reduction in proteinuria

These criteria are developed for use in patients with AL-amyloidosis; we suggest using these in patients with other subtypes of MGRS.

a If the initial dFLC is <50 mg/L, a dFLC <10 mg/L is considered low dFLC response (which can be considered very good partial response).

b Renal response requires stable or improved eGFR (decrease eGFR <25%); renal response predicts renal outcome, but is only applicable to patients with hematological response

Historically, AL-amyloidosis was associated with a very poor outcome. The use of melphalan–prednisone only slightly improved survival rates. The introduction of bortezomib-containing regimens, which resulted in a faster and better hematological response rate, contributed to improved renal response and higher survival rates. The addition of daratumumab resulted in more rapid FLC reduction, higher hematological complete response rates and improved progression-free survival.

The choice of therapy is based on patient characteristics, the eligibility for high-dose melphalan therapy with autologous stem cell transplantation (HDM-ASCT), center preference and drug reimbursement based on national authority regulations. It is expected that treatment modalities will change over the years. Therefore, we refer to the 2022 International Society of Amyloidosis guidelines, and the updates. Currently, the preferred induction treatment consists of a combination of daratumumab, cyclophosphamide, bortezomib and dexamethasone. However, there are unsolved questions, such as the optimal duration of daratumumab therapy, the added value of daratumumab in lower risk patients, the plausibility of sequential (FLC-guided) therapy, and the role of HDM-ASCT.

Regular measurement of serum FLC and evaluation of serum and urine by IFE will allow early detection of hematological relapse. The pathophysiology of amyloid fibril formation poses that a lower concentration of monomers is needed to accelerate the fibril growth in case of relapse in patients with persistent amyloid deposits Thus, it can be expected that clinical manifestations will occur more rapidly in case of a relapse. Early treatment appears to improve outcomes in large retrospective studies but prospective data are lacking. The choice for treatment for relapse depends on many variables such as duration of remission, previous treatment and clinical condition.

## CHAPTERS 3–9: NON-AMYLOID MGRS SUBTYPES [16–19]

Chapters 3–9 discuss other subtypes of MGRS. These chapters partly overlap, and often refer to Chapter 1, emphasizing the need for consultation of expert centers, the typical features of diagnostic kidney biopsy, the hematological evaluation and general principles of therapy. Importantly, these chapters provide information on the clinical characteristics and outcome of respective patients cohorts, which although limited by relative small numbers, provide data to be used in balancing risks and benefits of therapy. We summarize relevant points.

MIg deposition disease (MIDD) is defined by the presence of non-organized deposits of MIg chains, usually light chains (LCDD), or less commonly, heavy chains (HCDD) or both (LHCDD), along the basement membranes of various organs. Although nodular glomerulosclerosis is the characteristic glomerular pattern, glomerular injury may be absent, and linear staining along tubular basement membranes (TBM) is the pathognomonic diagnostic feature. In the absence of TBM staining, an alternative diagnosis should be considered. In some cases, IF staining may be negative which is attributed to the loss of the targeted epitope on the immunoglobulin or the lack of the involved immunoglobulin chain in routine IF (e.g. IgD). EM enables establishment or confirmation of a diagnosis of MIDD, since the powdery punctate deposits along the TBM (and often glomerular basement membrane and mesangium) are a characteristic finding. Although routine evaluation of a kidney biopsy with EM is advised, a diagnosis of MIDD can be made without EM, when the kidney biopsy is evaluated by an expert nephropathologist who confirms the typical IF and light microscopy findings. There is one caveat: in rare cases a diagnosis of LCDD by IF only has been made. This may represent an early phase of the disease, and underlines the importance of EM studies. Although patients with MIDD typically present with kidney injury, some patients may show extra-renal involvement, especially of the heart and the liver. Almost all patients with MIDD have abnormal FLC ratio and/or increased dFLC. In patients without such abnormalities, the diagnosis of MIDD should be reconsidered. Treatment will be according to the general approach depicted in Fig. 1, and adapted to the individual patient characteristics. In patients with renal-limited MIDD the merits of treatment are debatable in patients with eGFR <20 mL/min/1.73 m²), since in these patients treatment does not improve renal survival. Similarly, in the elderly patient survival time must be factored against the quality of life.

Cryoglobulinemia is defined by the presence of detectable circulating immunoglobulins that precipitate with cold temperature and dissolve with rewarming. Cryoglobulins are classified according to the composition of the immunoglobulins that are present in the cryoglobulin fraction. In type I there is a single MIg, type II consists of a MIg (often IgM) and polyclonal IgG. Type III contains mixed polyclonal immunoglobulin and there is no MIg. Thus, type III cryoglobulinemia is not included in the spectrum of MGRS.

The mere presence of cryoglobulins in serum is not sufficient to diagnose cryoglobulinemia as disease. The term cryoglobulinemic vasculitis (CryoVasc) is often used to indicate systemic disease caused by cryoglobulins. As such, cryoglobulin-associated disease can present variably with constitutional symptoms (asthenia, fatigue), skin abnormalities (palpable purpura, cutaneous ulcers), neurologic manifestations (sensory or motor neuropathy, cerebral infarction) and vasomotor injury (Raynaud, digital ischemia). Kidney involvement occurs in about one-quarter of patients with CryoVasc. On the other hand, approximately half of patients with cryoglobulin-associated kidney injury have no extra-renal manifestations. Isolated C4 depletion is a characteristic manifestation of cryoglobulinemia, in particular in type II cryoglobulinemia. Histologically, the most frequent pattern of injury is membranoproliferative glomerulonephritis with numerous infiltrating monocytes/macrophages. When present, intracapillary periodic acid–Schiff positive pseudo-thrombi are a helpful clue to the diagnosis. In IF, type I cryoglobulinemia shows staining for the involved MIg. In type II usually there is positive staining for both IgG and IgM, without clear light chain restriction. By EM, there are electron-dense deposits, which may be amorphous, although at higher magnifications substructures are often seen, with curvilinear, microtubular or annular appearance. The detection of cryoglobulins requires a very tightly controlled work-up. Failure to detect cryoglobulins is often explained by insufficient precautions, mostly occurring in the pre-analytical phase, i.e. the period that starts with blood withdrawal. It is essential that blood always remains at a temperature of 37°C, during withdrawal, transport, coagulation and serum separation. Although cryoglobulins often precipitate within hours to days, slower precipitation can occur and the precipitate may be visible only after 7 days. Therefore, in patients with suspected cryoglobulinemia it is advised to repeat the evaluation and adhere to the long waiting time. In some patients a diagnosis of cryoglobulinemic glomerulonephritis can be made even in the absence of detectable serum cryoglobulins (seronegative CryoVasc). By definition, in these patients the diagnosis was based on the observation of intracapillary pseudo-thrombi in the kidney biopsy.

Type I cryoglobulinemia is often associated with a hematological malignancy. Although cryoglobulinemia type II can be the consequence of a hematological malignancy, usually an IgM-secreting hematologic disorder, other underlying causes are more often identified. In many countries, hepatitis C virus (HCV) infection was the underlying cause in 60%–95% of patients with Type II cryoglobulins. In 50%–70% of patients with non-HCV associated CryoVasc, other causes are detected, including hepatitis B infection, Sjögren’s syndrome, or other infections or systemic autoimmune diseases. In patients with CryoVasc the underlying cause is the primary target of therapy. If no underlying cause and no hematological malignancy is identified, patients with type I or type II cryoglobulinemia will fulfill the criteria for MGRS.

To advise treatment the patient should have signs or symptoms that are typical for CryoVasc or there should be unequivocal histological evidence of cryoglobulin-induced kidney injury. Patients with CryoVasc are often aged, and are at risk of infections related to treatment. Thus, treatment must be selected based on comorbidity, age, the severity of the disease, the underlying cause and previous treatment. In patients with kidney injury due to cryoglobulins, the main goal of therapy is to improve kidney function, reduce proteinuria and prevent progression to ESKD. In a patient with a detected BM clone (more often found in cryoglobulinemia type I than in type II), clone-targeted therapy is preferred. In patients with no detectable clone (most often type II) initial treatment with rituximab is suggested, taking into account the risk of a flare caused by immune complex formation. In selected patients, pretreatment with cyclophosphamide and steroids is advised. In patients with high cryoglobulin levels and signs/symptoms compatible with perfusion defects, hyperviscosity or severe acute kidney injury, rapid removal of the cryoglobulins requires plasmapheresis combined with therapy directed at reducing the production of the involved immunoglobulins.

Proliferative glomerulonephritis with MIg deposits (PGNMID): in its first description, PGNMID was defined by a proliferative pattern of glomerular injury, characterized by glomerular non-organized deposits positively staining for a monotypic IgG. It is now evident that this entity also includes non-proliferative patterns of glomerular injury, and glomerular injury caused by other immunoglobulin classes or even light chains only. Most studies showed that in 60%–70% of patients with PGNMID serum immunofixation was negative and the FLC ratio normal. In these patients, the likelihood of finding BM abnormalities was also very low. Therefore, a diagnosis of PGNMID was critically dependent on expert evaluation of a kidney biopsy, and specifically positive IF staining for a monotypic immunoglobulin. A very recent study used sensitive sequencing techniques to determine monoclonality, and showed that there was no evidence for monoclonality in the majority of patients with PGNMID (especially PGNMID with IgG3κ). This finding is important, and explains the heterogeneity in presentation, outcome and response to therapy. Patients who present with limited kidney injury may develop spontaneous remission. Treatment decisions should be primarily guided by the presence of a BM clone, kidney injury parameters and the (expected) rate of progression of kidney disease. Treatment follows the algorithm as proposed in Fig. 1.

Immunotactoid glomerulopathy (ITG) is characterized by the presence of Congo red–negative, immunoglobulin-derived, organized deposits with a microtubular structure in the mesangium and glomerular capillary wall. A diagnosis of ITG thus requires EM evaluation of the kidney biopsy. The deposits stain positive for IgG in IF. Typically, light chain restriction is observed in the majority of patients, thus defining ITG as a subtype of MGRS. Many patients with ITG will develop ESKD without therapy. Therefore, most patients will benefit from hematological therapy, preferably clone-directed. In patients with ITG and no detected BM clone, empirical therapy is advised, and start of therapy should be weighed against age, comorbidity and life expectancy. In some patients with ITG, light chain restriction is absent. There are no data to support treatment advice for patients with polyclonal ITG. However, some patients with “polyclonal” ITG may represent monoclonal ITG. Therefore, in patients with polyclonal ITG, rapid disease progression and/or severe disease empirical treatment as given for monoclonal ITG can be considered.

In the consensus report, we discuss fibrillary glomerulonephritis with MIg deposits, although there is debate as to whether this constitutes a separate disease entity. Fibrillary glomerulonephritis is defined as a glomerular disease, characterized by the presence of immunoglobulin derived, fibrillary deposits in the mesangium and/or glomerular capillary wall. Usually, these deposits consist of polyclonal IgG, are Congo red negative, and stain positive for DNAJB9. Only in very rare cases are the deposits composed of a MIg. We underline that in patients with suspected monoclonal fibrillary glomerulonephritis, all efforts must be made to confirm the diagnosis. This includes additional studies using pronase digestion to unmask undetected immunoglobulin light chains, DNAJB9 staining and IgG subclass staining. If a diagnosis of monoclonal fibrillary glomerulonephritis is made, we advise management according to patients with ITG.

Light-chain proximal tubulopathy (LCPT) and light chain crystalline podocytopathy (LCCP) are discussed in one chapter, since both entities are defined by the presence of crystalline light chain inclusions in the proximal tubular cells and podocytes respectively. Clinically, these diseases are quite different.

We emphasize the difficulties in diagnosing LCPT. In the prototypical patient, there is evidence of proximal tubular dysfunction, with features of Fanconi syndrome. The kidney biopsy shows crystalline inclusions in the proximal tubules, with IF staining showing light chain restriction (most often κ). The culprit light chain can be detected in serum and urine. There are however caveats. (i) In most patients, the Fanconi syndrome is “incomplete,” thus some of the typical elements of Fanconi syndrome (i.e. aminoaciduria, normoglycemic glycosuria, proximal renal tubular acidosis (RTA), hypophosphatemia and hypouricemia with increased fractional excretion of phosphate or uric acid) are lacking. Of note, interpretation of serum test results for the evaluation of Fanconi syndrome is difficult in patients with kidney dysfunction: patients with reduced eGFR might have (low)-normal serum phosphorus and serum uric acid despite increased fractional excretion. Also, in patients with low eGFR acidosis is expected and might not easily be recognized as sign of proximal RTA. (ii) Routine IF staining on frozen tissue is often falsely negative, and thus IF on pronase-digested paraffin-embedded tissue should be done in all patients with suspected LCPT and negative routine IF. (iii) In routine light microscopy, crystals are often not easily detected, and EM evaluation is necessary to demonstrate the cytoplasmic crystalline inclusions.

In adults, LCPT is the major cause of Fanconi syndrome. The presence of complete Fanconi syndrome in patients with a MIg and abnormal FLC in serum and urine is considered sufficient to diagnose LCPT. In such patients a kidney biopsy is not deemed necessary for a diagnosis of LCPT, unless the patient presents with features that are not typical for LCPT, such as severe proteinuria or rapid deterioration of kidney function. Adult patients who present with features of Fanconi syndrome should be evaluated for the presence of increased light chains in serum and urine. On the other hand, patients with “MGUS” and increased light chains in urine should be evaluated for proximal tubular dysfunction.

In some patients with LCPT crystalline inclusions may be absent. Still, a diagnosis of LCPT can be made when proximal tubular injury is present and kidney biopsy demonstrates intracellular fibrillary or amorphous deposits or irregular large lysosomes. Notably, in some patients who present with kidney failure proximal tubule light chain restriction may be observed in the absence of Fanconi syndrome or crystals. It is debated whether this represents LCPT. We refer to the full report, which questions the role of the light chain in causing kidney injury and suggests that the biopsy finding may simply reflect normal physiology in patients with an elevated level of FLC.

Although LCPT is a renal limited disease, patients may present with metabolic complications such as bone disease resulting from phosphate loss or hypokalemia as a consequence of proximal RTA. With respect to therapy, the metabolic complications should be evaluated, and patients may need treatment such as vitamin D supplementation, or correction of acidosis or hypophosphatemia. With respect to hematological treatment, a “wait and see” policy is sometimes preferred in view of the often slow progression rate. Patients with metabolic complications, or patients with (high risk of) kidney function deterioration may benefit from hematological treatment, targeting the abnormal clone, while measuring monitoring FLC to monitor treatment response.

LCCP has only very recently been described and proposed as separate disease entity. LCCP is characterized by the presence of light chain restricted, crystalline deposits in the podocyte. Identification of these crystalline structures often requires EM evaluation. Routine IF on frozen tissue is often falsely negative, and identification of the culprit light chain requires IF or immunohistochemistry on paraffin-embedded tissue after pronase digestion. Many patients with LCCP in parallel show histological features of LCPT, and should be evaluated for proximal tubular dysfunction. Since LCCP is ultrarare, there are limited data. Most patients with LCCP present with moderate to severe proteinuria. The available data suggest that in LCCP renal response is observed in patients with hematological response to therapy. However, in view of the paucity of data, patient characteristics such as age, severity of kidney injury and rate of progression should be balanced against the benefits and risks of aggressive therapy.

MIg-associated C3 glomerulopathies (C3G): the C3 glomerulopathies (C3G) are a recently defined subgroup of rare glomerular diseases. Diagnosis and management requires expertise in nephropathology, laboratory medicine including complement assays, and genetics. C3G are kidney diseases attributed to alternative complement pathway dysregulation and characterized by dominant deposition of C3. It includes C3 glomerulonephritis (C3GN) and dense deposit disease (DDD). C3G is defined by dominant staining for C3 in the glomeruli by IF, with intensity at least 2+ higher than the staining for IgG or other immunoglobulins. Based on the findings on EM, C3 glomerulopathies are subdivided in C3GN and DDD.

Complement dysregulation can be caused by polyclonal or monoclonal autoantibodies that activate the C3 convertase or prevent its inhibition. Less frequently, complement dysregulation is caused by inherited abnormalities in the complement system (e.g. mutations in genes that encode complement regulatory proteins). Genetic and polyclonal auto-immune C3G are typically observed in children and young adults. C3G associated with a monoclonal antibody (MGRS-C3G) is typically (but not exclusively) observed in patients >50 years of age.

In routine clinical practice it is impossible to prove that the MIg interferes with the complement cascade. Therefore, the diagnosis is made by exclusion. In elderly patients (>50 years), the presence of a MIg is usually considered sufficient to make a diagnosis of MGRS-C3G.

C3G is usually a renal limited disease, although a recent study suggests that digital ischemia can develop as rare extra-renal manifestation of C3G. Routine evaluation is not needed to search for subclinical extrarenal disease manifestations. With respect to treatment, a “wait and see” policy may be justified in patients with old age and slow progression of renal disease, taking into account life expectancy, comorbidity and treatment associated toxicity.

## CHAPTER 10: KIDNEY TRANSPLANTATION IN MGRS [20]

Although hematological treatment has undoubtedly improved outcome in patients with MGRS, some patients will develop ESKD, especially when presenting with reduced eGFR at initial diagnosis. Kidney transplantation should be discussed in every patient with MGRS who is otherwise eligible for transplantation. In general, it is accepted that patients with MGRS who have been treated and who have reached complete or very good partial hematological response can be accepted as candidates for kidney transplantation, since there is a low risk of recurrence of the original disease and ensuing graft loss, and a low risk of progression to a hematological malignancy. This holds for all types of MGRS. Obviously, acceptance for kidney transplantation requires that the patient fulfils all other criteria mentioned in the guidelines for evaluation of a kidney transplant candidate.

There is much debate on kidney transplantation in patients with MGRS, who are untreated or have not reached complete or very good partial response. To avoid any risk, some authors suggest that patients with MGRS who have not achieved hematological response should not be accepted for kidney transplantation. However, this recommendation is not based on hard evidence. The discussion in the full consensus report illustrates that decision-making in such patients is quite complex, requires balancing risks and benefits, and must take into account patient preferences and country specific transplant context and guidelines. To be able to balance risks and benefits, detailed and specific information is needed, as illustrated in Table 8.

**Table 8:** Kidney transplantation in patients with MGRS: the most relevant questions.

Question	Evaluation	Relevance/comments
What is the original kidney disease diagnosis?	Confirmation that ESKD was caused by an M-protein. Ascertain the subtype of MGRS	Many patients with MGUS and CKD do not have MGRSEstablishing the correct diagnosis is pivotalThe subtype of MGRS determines risk of extrarenal disease and recurrence rate after kidney transplantation
What was the original kidney disease course?	Evaluate rate of progression in the native kidney	Rapid deterioration of eGFR in the native kidney may herald high risk of early recurrence and graft loss
What is hematological status?	Review of hematological diagnosis and treatmentEvaluate M-protein levels and free light chainsDefine treatment response (CR, VGPR, PR, NR)	Patients with CR/VGPR can be accepted for kidney transplantationAn individualized approach is needed in untreated patients or patients with PR/NRHigh levels of M-protein or high dFLC are considered unfavorableLow C3 in patients with C3G are unfavorable
Is there evidence of extrarenal disease?	Evaluation depending on MGRS subtype; examples are heart, autonomic neuropathy, gastro-intestinal tract in amyloidosis, MIDD, cryoglobulinemia	Presence of extrarenal disease is a contraindication for kidney transplantation without adequate response to (additional) hematological therapyExtrarenal disease also affects posttransplant course (e.g. cardiac performance, drug absorption)
What is the recipient’s CKD stage?	Is patient already on dialysis?	A recipient with CKD stage 4/5 not on dialysis benefits from preemptive kidney transplantation, and has increased risk of side effects of hematological therapy (specifically HDM-ASCT)
Is a living donor kidney available?	Donor accepted and ready for donation?	Availability of a living donor assures short waiting time, and allows preemptive transplantationA living donor kidney does not add to shortage of organs
What is the expected waiting time?	Depends on availability of living donor, and patient- and country-specific factors	A long waiting time necessitates pretransplant hematological therapy
What is the rate of recurrent disease and the time to recurrence?	Depends on MGRS subtype and pretransplant native kidney disease course	A high risk of early recurrence argues against preemptive kidney transplantation without prior hematological therapy. The most feared subtype is C3 glomerulopathy
Can recurrent disease be treated?	Discuss with hematologist which treatment modalities are still an option for the patient	Most patients with MGRS recurrence will respond to hematological therapy. Lenalidomide is associated with rejection post-transplantInformation on pretransplant hematological therapy will dictate possible treatment options
What are recipient’s characteristics?	Age and comorbidity	Usual risk factors for kidney transplantationLong-term patient and graft survival might be less relevant in the elderly patient
Kidney shortage	Country specific	From a societal perspective a deceased donor kidney should be used in a patient with *a priori* acceptable graft survival

See full consensus report for detailed discussion and references. CR, complete response; VGPR, very good partial response; PR, partial response; NR, no response.

**Figure 2:**
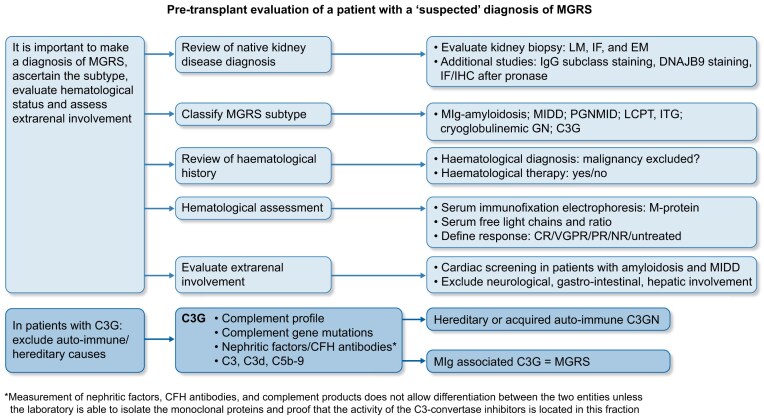
Pre-transplant evaluation of a patient with a “suspected” diagnosis of MGRS.

**Figure 3:**
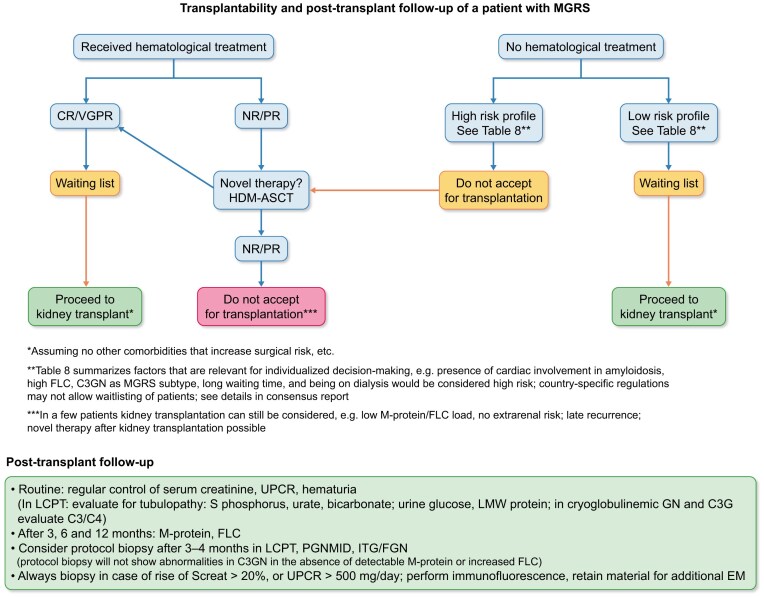
Transplantability and post-transplant follow-up of a patient with MGRS.

It is evident that the decision process requires consultation in a multidisciplinary team, is dependent on individual patient characteristics and also differs between countries. We refer to the consensus report and the referenced literature for details. Figures 2 and 3 provide an algorithm which illustrates the decision process. Overall, we suggest that kidney transplantation is contraindicated in patients with MIg-associated C3G who have not reached hematological complete or very good partial response. In these patients, recurrence of disease develops early after kidney transplantation, within weeks to months, likely explained by the activation of complement inherent to the transplantation procedure (ischemia–reperfusion, surgery, delayed graft function, dialysis, rejection). Moreover, these recurrences are not easy treatable, and cause graft loss. In contrast, kidney transplantation can be considered in patients with other subtypes of MGRS, and the ultimate decision is based on a balanced risk estimate using the answers to the questions posed in Table 8.

## CONCLUSION

This executive summary introduces the European Union consensus report on the diagnosis and treatment of MGRS. This summary provides an introduction to MGRS, with a general overview of diagnosis and management of patients with MGRS. Key issues are listed in Table 9. MGRS are rare diseases, and there is a large heterogeneity, both between and within subtypes of MGRS. Therefore, there is no high-quality evidence to support recommendations. We emphasize that consultation with expert centers is necessary, and management must be individualized, taking into account patient and disease characteristics. This summary does not substitute for the full consensus report, which includes the rationale and an extensive discussion of the relevant literature.

**Table 9:** Key summary points.

In patients with CKD there is no evidence to support routine screening of serum for the presence of a MIg In patients with MGUS and CKD there is no evidence to support routine kidney biopsy A diagnosis of MGRS requires expert nephropathological evaluation of a kidney biopsy Be aware of pitfalls: A serum M-protein can be absent in patients with MGRS (low sensitivity) Interpretation of FLC ratio should take into account eGFR, the manufacturer of the assay and the appropriate reference values IF studies can be false negative (may need multiple antibodies and/or pronase digestion) Light chain restriction does not prove monoclonality A BM clone can be absent in patients with MGRS AL-amyloidosis is an emergency, necessitating immediate diagnosis, and early start of therapy Most patients with MGRS will benefit from (clone-directed) therapy hematological therapy The choice of therapy must be adapted to the individual patient MGRS is not a malignancy In renal-limited MGRS treatment should prevent development of ESKD Treatment may be futile, especially in the frail elderly patient In patients with cryoglobulinemia, there is a risk of flare when using rituximab

## Supplementary Material

sfag163_Supplemental_File

## Data Availability

The data underlying this article are available in the article and in its online Supplementary material.
