# Supplementary material for: Executive summary of the European consensus report on the diagnosis and treatment of monoclonal gammopathy of renal significance
Source: Clin Kidney J. 2026 May 26;19(6):sfag163. doi: 10.1093/ckj/sfag163 (PMC13284707; doi:10.1093/ckj/sfag163)
Supplement: sfag163_Supplemental_File [file sfag163_supplemental_file.docx]

**Monoclonal Gammopathy of Renal Significance:**

**European consensus report on diagnosis and treatment**

**Working group**

**Prof. em. Jack F Wetzels MD, PhD**; Dept Nephrology, Radboud University Medical Center, Nijmegen The Netherlands (chair)

**Ute Hegenbart MD**; University Hospital, Amyloidosis center, Heidelberg, Germany (co-chair)

**Prof. Ben Sprangers MD, PhD** ;Department of Nephrology, Ziekenhuis Oost-Limburg, Genk, Belgium. (co-chair)

and Department of Immunology and Infection, Biomedical Research Institute, UHasselt, Diepenbeek, Belgium.

**Camille Cohen, MD,PhD**: Associate Professor Nephrology, Hôpital Bichat; Center for Inflammation Research (CRI), INSERM U1149, Paris France

**Viviane Gnemmi MD, PhD**; Dept Pathology, Lille, France

**Amir Shabaka, MD**. Nephrology Department, Hospital Universitario La Paz, Madrid, Spain

**Prof. Gema Fernandez-Juarez, MD,PhD**: Nephrology Department, Hospital Universitario La Paz, Madrid, Spain

**Dominique van Midden MD;** Dept Pathology, Radboudumc Nijmegen The Netherlands

**Paolo Milani, MD, PhD** Department of Molecular Medicine, University of Pavia, Italy; Amyloidosis Research and Treatment Center, Foundation "Istituto di Ricovero e Cura a Carattere Scientifico (IRCCS) Policlinico San Matteo",Italy.

**Eric Steenbergen MD,PhD**; Dept Pathology, Radboudumc Nijmegen The Netherlands

**Marguerite Vignon MD,PhD**; Hopital Cochin, APHP, Paris, France

**Benjamin Wilde, MD,** Dept of Nephrology, University Hospital Essen, University Duisburg-Essen, Germany

**Acknowledgements:**

**This project has been supported by ERKNet. ERKNet is funded by the European Union within the framework of the EU4Health Programme 2021-2027.**

**We thank the following colleagues for critical review of the draft document and their feedback:**

**Prof. Dario Roccatello, University of Turin, Director of the Center of Research on Immunopathology and Rare Diseases (CMID), San Giovanni Bosco Hospital, Turin, Italy.**

**Prof. Amelie Dendooven, Dept Pathology, UZ Gent, Belgium**

**Prof. Amaryllis van Craenenbroeck, Dept Nephrology, UZ Leuven, Belgium**

**Dr. Sandra Croockewit, Dept Hematology, Raboudumc, Nijmegen, The Netherlands**

**Abbreviations**

AL-amyloidosis light chain amyloidosis

AH-amyloidosis heavy chain amyloidosis

ALH-amyloidosis light&heavy chain amyloidosis

ACEi Angiotensin converting enzyme inhibitors

ARB Angiotensin Receptor blocker

BJP bence jones protein

C3G C3-glomerulopathy

C3GN C3-glomerulonephritis

CR complete response

CryoVasc cryoglobulinemic vasculitis

DDD Dense Deposit Disease

EM Electronmicroscopy

ESKD End Stage Kidney Disease

FGN fibrillary glomerulonephritis

FLC free light chains

FLCr free light chain ratio

HCDD heavy chain deposition disease

HDM-ASCT high dose melphalan-autologous stem cell transplant

IF immunofluorescence

IF-F immunofluorescence on frozen tissue

IF-P immunofluorescense after protease digestion

IHC immunohistochemistry

ITG immunotactoid glomerulopathy

LCDD light chain deposition disease

LECT leucocyte chemotactic factor 2

MIg monoclonal immunoglobulin

MIg-amyloidosis Monoclonal immunoglobulin amyloidosis (includes AL, AH, ALH amyloidosis)

MGRS monoclonal gammopathy of renal significance

MGUS monoclonal gammopathy of unknown significance

MIDD monoclonal immunoglobulin deposition disease

NR no response

PGNMID proliferative glomerulonephritis with monoclonal immunoglobulin deposits

PR partial response

SIFE serum immunofixation

SPEP serum protein electrophoresis

UACR urine albumin-creatinine ratio

UIFE urine immunofixation

UPCR urine protein-creatinine ratio

UPEP urine protein electrophoresis

VGPR very good partial response

**Note:** in the text *hematologic therapy* is used to encompass all forms of therapy that are used to treat patients with MGRS. This includes corticosteroids, alkylating agents, proteasome inhibitors, Immunomodulatory drugs, anti-B cell therapy and anti-Plasmacell therapy.

**Introduction:**

Monoclonal gammopathy of renal significance (MGRS) is defined by kidney damage caused by (or attributed to) a Monoclonal Immunoglobulin (MIg), in the absence of a B cell or plasma cell clone that meets the criteria for specific hematological therapy. MGRS was only recently recognized as disease entity, and all subtypes of MGRS are (very) rare. The European Rare Kidney Disease network (ERKnet) identified MGRS as a (group of ) diseases that lacked clinical practice guidelines. A multidisciplinary working group has addressed this need. The working group was composed of nephrologists, hematologists, and renal pathologists. In MGRS, making a proper diagnosis is of utmost importance. Furthermore, in these rare diseases there are no or very few randomized therapeutic trials. Therefore, it is impossible to make evidence-based recommendations. Since clinical practice guidance is often based on expert opinion, and will vary between countries and continents, the working group was limited to members of countries participating in ERKnet, and the consensus report is written from a EU perspective. The members of the working group identified relevant literature and provided expert opinion. All chapters were discussed extensively, and the consensus report provides the conclusions.

The report consists of 10 Chapters. Each Chapter provides a summary of advisory statements, followed by a brief rationale, and extensive supportive evidence containing relevant literature references. Chapter 1 provides an overview of diagnosis and management of MGRS, chapters 2-9 discuss the most important subtypes of MGRS, and Chapter 10 summarizes aspects of kidney transplantation in patients with MGRS.

The British Society of Haematology recently published a good practice paper on the diagnosis and management of MGRS. (1). Overall, there is agreement between both reports. There are some differences. These are summarized below. We refer the reader to the supportive evidence text in this report to value the conclusions.

| British Society of Haematology | This document (ERKnet consensus report) |
| --- | --- |
| All patients referred to nephrology should be tested using SPEP/SIFE and FLC assay | Restrictive testing based on clinical criteria |
| Patients should be accepted for kidney transplantation only if treated and having developed complete or very good partial response | Kidney transplantation can be considered in untreated patients or in patients with partial response |
| LCPT can occur without crystalline inclusions | A diagnosis of LCPT should be questioned in the absence of crystalline inclusions and features of Fanconi syndrome |

We expect that the diagnosis and management of MGRS will be further refined in future. One example of novel findings is the discovery that many patients with monotypic PGNMID may not have a monoclonal disorder, and may no longer qualify for a diagnosis of MGRS (see discussion in Chapter 5). (2)

We hope that this consensus document assists clinicians in diagnosing MGRS, supports discussions in a multidisciplinary team, and is of help in selecting and discussing management with patients.

**Chapter 1: Monoclonal Gammopathy of Renal Significance (MGRS): definition, diagnosis and general aspects of treatment**

**Introduction: definition and nomenclature of MGRS**

MGRS is defined as a disease entity characterized by kidney damage caused by (or attributed to) a Monoclonal Immunoglobulin (MIg), in the absence of a B cell or plasma cell clone that meets the criteria for specific hematologic therapy. Thus, this definition of MGRS includes patients with kidney injury attributed to a MIg in patients with conditions such as smouldering multiple myeloma or indolent lymphoma/CLL.(3)

Although the pathogenesis of MGRS is not fully elucidated, kidney injury is attributed to either a direct or indirect mechanism. (4) In the former, kidney injury is caused by the deposition of a MIg (intact or truncated, single heavy or light chain) in the kidney, with the specific type of kidney injury determined by the involved compartment (glomerulus, tubules, interstitium, vasculature) and the biochemical characteristics such as size, charge, and cleavage sites of the monoclonal immunoglobulin. Alternatively, kidney injury can be caused by indirect effects, as occurs in C3-nephropathy, attributed to interference of the MIg with normal complement alternative pathway regulation.

There are many subtypes of MGRS. The distinction between these various subtypes is important, in view of the differences in diagnostic work-up, outcome, and treatment-response. An overview of the subtypes was given in the 2019 consensus report of the International Kidney and Monoclonal Gammopathy research group (IKMG).(3). Table 1.1 (first column) provides an overview of the various forms of MGRS, arbitrarily grouped according the presence of extrarenal manifestations, and the suggested mechanism of toxicity of the MIg (direct vs indirect). A slightly adapted classification was used by the British Society of Haematology, in the good practice paper on diagnosis and management of MGRS.(1) Most recently, a consensus report of the Renal Pathology Society and the IKMG provided an update on pathological description and terminology.(5). This updated classification is added to Table 1.1 (second column).

Table 1.1: Classification of MGRS&

| MGRS subtypes (2019)(3) | MGRS subtypes (2025)(5) |
| --- | --- |
| MGRS with extrarenal involvement | |
| Amyloidosis  AL-amyloidosis (light chains)  AH-amyloidosis (heavy chains)  ALH-amyloidosis (light & heavy chains) | Amyloidosis  AL-amyloidosis (light chains)  AH-amyloidosis (heavy chains)  ALH-amyloidosis (light & heavy chains) |
| MIg-deposition disease (MIDD)  Light chain deposition disease (LCDD)  Heavy chain deposition disease (HCDD)  Light &heavy chain deposition disease (LHCDD) | MIg-deposition disease (MIDD)  Light chain deposition disease (LCDD)  Heavy chain deposition disease (HCDD)  Light &heavy chain deposition disease (LHCDD)  Light chain deposition disease by IF only |
| Cryoglobulinemic glomerulonephritis (type I and type II) | Cryoglobulinemic glomerulonephritis types I and II |
| Crystalglobulin nephropathy# | Crystal-globulin induced nephropathy# |
| MGRS renal limited, direct effect | |
| Proliferative glomerulonephritis with monoclonal immune deposits (PGNMID), includes membranous nephropathy | Proliferative glomerulonephritis with MIg deposits (PGNMID), subtypes according variant (IgG, IgM, IgA, light chain only) |
|  | Monoclonal membranous nephropathy |
| Immunotactoid nephropathy | Monoclonal immunotactoid glomerulopathy |
| Fibrillary glomerulonephritis with MIg deposits |  |
| Light chain proximal tubulopathy | Crystalline light chain proximal tubulopathy |
|  | Non-crystalline light chain proximal tubulopathy |
|  | Light chain crystalline podocytopathy |
|  | Crystalline cryoglobulinemic glomerulonephritis type I # |
| Crystal storing histiocytosis# | light chain crystal storing histiocytosis # |
| MGRS renal limited, indirect effect | |
| C3-glomerulopathy | C3-glomerulopathy due to MIg  (subtypes C3GN and DDD) |
| Thrombotic microangiopathy## |  |
|  | Intracapillary monoclonal IgM nephropathy # |

**Adapted from (3),(1),(5)**

&Other subtypes of apparent MGRS have been described (case reports). These include anti-GBM nephritis with monotypic deposits and IgA nephropathy with monotypic lambda deposits. In these cases often no MIg is detected and it remains uncertain if this really reflects monoclonal Ig associated disease.

Very rarely, patients may present with > 1 subtype e.g. LCDD + amyloidosis, or LCDD + LCPT. Combination of > 1 subtype are not discussed in the document, in general management should be according management of patients with the most severe component (e.g amyloidosis prevails over LCPT).

# these are very rare entities and not discussed in this document.

## An association between the presence of a MIg and TMA has been described (6) (7). In the absence of definitive data this entity is not further discussed in this consensus document.

**MGRS: Diagnosis and general aspects of treatment**

This chapter provides an overview of the diagnosis and management of patients with “suspected” MGRS. We address the role of kidney biopsy in patients with known “MGUS”, and the diagnostic accuracy of assessment of a MIg in the serum or urine of a patient with chronic kidney injury. We describe the evaluation of the kidney biopsy, the role of additional laboratory and hematologic evaluation, and general treatment strategies. We discuss the pharmacokinetics of the drugs that are used in hematologic treatment of patients with MGRS. Information on specific disease entities is given in Chapters II-IX

**Summary statements**

***We do not advise a routine kidney biopsy in patients with MGUS. A kidney biopsy should be considered if there is evidence of proteinuria, deterioration of eGFR, proximal tubular dysfunction (compatible with (in)complete Fanconi syndrome), or evidence of extrarenal manifestations that are suggestive of a MIg-associated disease (diarrhea, heart failure, neuropathy, low systolic BP, liver dysfunction).***

***We do not advise routine screening of serum or urine for the presence of a MIg in patients with UPCR < 1g/g (proteinuria < 1g/day) and/or stable eGFR > 60 ml/min/1.73m^2^ (or > 45 ml/min/1.73m^2^ in the elderly aged > 70 years). Prebiopsy screening of serum or urine for the presence of a MIg is not evidence-based but can be considered. There is variation between country-specific guidelines.***

***Adequate handling and interpretation of kidney biopsies is essential to diagnose (or exclude with certainty) MGRS. A detailed evaluation is required and includes light microscopy, immunofluorescence and/or immunohistochemistry, and electron microscopy studies. In some patients, additional studies should be considered.***

***We recommend the search for a MIg in serum and/or urine, with appropriately sensitive laboratory techniques, in patients with a histologically documented MGRS. A similar approach is recommended in patients with kidney injury patterns that are typically associated with a MIg (e.g. C3GN).***

***In patients with documented MGRS evaluation should include bone marrow examination including flowcytometry.***

***In patients with MGRS we recommend treatment targeting blood pressure, proteinuria, and cholesterol, and life style measures as indicated for patients with CKD.***

***In patients with MGRS the choice of treatment should be guided by disease and patient specific characteristics. In patients with a documented bone marrow abnormality, and who are considered candidates for therapy, clone-directed hematologic therapy is preferred.***

***We advise consultation with an expert center for all patients with MGRS.***

**Rationale**

***We do not advise a routine kidney biopsy in patients with MGUS. A kidney biopsy should be considered if there is evidence of proteinuria, deterioration of eGFR, proximal tubular dysfunction (compatible with (in)complete Fanconi syndrome), or evidence of extrarenal manifestations that are suggestive of a MIg-associated disease (diarrhea, heart failure, neuropathy, low systolic BP, liver dysfunction).***

Patients with a MIg in the serum or urine should be evaluated according existing guidelines for MGUS. The prevalence of MGUS is high, especially in the elderly population. This elderly population also often has CKD defining abnormalities (such as eGFR < 60 ml/min/1.73m^2^ or urinary albumin/creatinine ratio > 30 mg/g), attributed to hypertension, diabetes, and obesity. In contrast, the incidence of MGRS is very low. While MGRS must be considered in each patient with MGUS and kidney injury, there is no evidence to support a non-restrictive biopsy strategy. To allow early detection of MGRS as underlying disease, we advise careful monitoring of patients with MGUS and subtle kidney injury. Patients should be followed according the KDIGO-CKD guidelines, with more frequent visits in case of UPCR > 0.3 g/g. MIg-amyloidosis and MIDD are systemic diseases, and often present with extrarenal manifestations. The presence of extrarenal manifestations should increase the suspicion of MGRS. A kidney biopsy should be considered in patients with new onset or increasing proteinuria ( > 0.5 - 1g/day), or patients with progressive deterioration of eGFR, patients with features of Fanconi syndrome, or in patients with limited kidney injury (proteinuria < 1g/day, stable eGFR even if below normal) but (suspected) extrarenal manifestations. The role of hematuria in decision making is debated, although the presence of new onset glomerular hematuria argue in favor of a kidney biopsy. In the follow-up of patients with MGUS and limited kidney injury, we caution against the use of UACR as sole measure of proteinuria, since this will not enable to detect increasing low molecular weight or light chain proteinuria. Therefore, measurement of UPCR is preferred. The ratio UACR/UPCR can be used to guide requests for additional urine evaluation.

***We do not advise routine screening of serum or urine for the presence of a MIg in patients with UPCR < 1g/g (proteinuria < 1g/day) and/or eGFR > 60 ml/min/1.73m^2^ (or > 45 ml/min/1.73m^2^ in the elderly aged > 70 years). Prebiopsy screening of serum and urine for the presence of a MIg is not evidence-based but can be considered. There is variation between country specific guidelines.***

This topic is debated, and our position differs from other commentaries. The prevalence of MGRS is low, and therefore routine screening in patients with CKD of serum with SPEP/SIFE, has limited positive predictive value. Moreover, sensitivity is relatively low, thus screening cannot be used to rule out MGRS (although it is debated whether glomerular diseases with monotypic deposits can be considered MGRS in the absence of a documented MIg, see also Chapter 5 PGNMID). There is also no robust data to support the FLC assay as screening tool, especially considering its costs. Of note, country specific guidelines may prefer routine screening of serum and urine with SPEP in patients with kidney injury. Importantly, in patients with CKD in whom the decision to perform a biopsy is equivocal, and no alternative diagnosis for kidney disease is likely, evaluation of serum for the presence of an M-protein, and measurement of free light chains might help in decision making. Many centers prefer screening for MIg in patients who are planned for a kidney biopsy, to guide the pathologist in evaluating the kidney biopsy (performing EM or paraffin immunofluorescence). This might be a rational approach in unexperienced centers or centers that use a limited kidney biopsy evaluation. In patients with nephrotic syndrome, presenting with manifestations that are compatible/suggestive of amyloidosis MIg testing (by serum or urine IFE and FLC assay) may guide the evaluation of the patient by prioritizing a fat biopsy to document AL amyloid deposition (see chapter II).

***Adequate handling and interpretation of kidney biopsies is essential to diagnose (or exclude with certainty) MGRS. A detailed evaluation includes light microscopy, immunofluorescence and/or immunohistochemistry, and electron microscopy studies. In some patients, additional studies should be considered***

A diagnosis of MGRS heavily relies on the histopathological evaluation. A diagnosis of MGRS does not require the presence of a monoclonal Ig in serum or urine. Therefore, independent of prebiopsy laboratory evaluation, the kidney biopsy should be properly evaluated in a laboratory with expert nephropathology service, including the use of appropriate staining for light microscopy, and IF, if necessary IF on paraffin embedded tissue after pronase/proteinase digestion, and preferably evaluation of the biopsy by EM. In selected cases, especially in patients with amyloidosis, additional techniques may be required to firmly establish the diagnosis and amyloid subtype (see detailed information in chapter II). Laboratories lacking EM or other specialized techniques should collaborate with referral laboratories to secure adequate work-up.

***We recommend the search for a MIg in serum and/or urine, with appropriately sensitive laboratory techniques, in patients with a histologically documented MGRS. A similar approach is recommended in patients with kidney injury patterns that are typically associated with a MIg (e.g. C3GN)***

In patients with MGRS, the culprit MIg can often be detected in serum or urine. With the availability of FLC assays, urine testing can be limited to patients with negative SPEP/SIFE and “normal” serum FLC results. Thus, in patients with MIg deposition in the kidney as well as in patients with C3GN, we advise initial screening of serum for the presence of a MIg using electrophoresis and immunofixation and FLC analysis. It is important to be aware that immunofixation is not a very sensitive technique, thus small amounts of a MIg may escape detection (<1 g/l). The availability of (expensive) commercial methods to measure free light chains has been pivotal in the detection of subtle hematologic abnormalities in patients with MGRS. It is relevant to know that there are different FLC assays: these assays provide dissimilar results (even more in patients with kidney failure) and should never be used interchangeably.

In patients with negative/normal serum findings additional urine evaluation is advised. Documenting the presence of a MIg in serum or urine, or finding an abnormal κ/λ ratio serves multiple goals: it confirms the diagnosis in patients with biopsy proven MGRS, it provides support for a diagnosis in patients with suspected MGRS with kidney injury attributed to an indirect effect of the MIg (eg C3GN), and it provides a biomarker that can be used to evaluate hematologic treatment response.

***In patients with a documented MGRS evaluation should include bone marrow examination including flowcytometry.***

In patients with MGRS, identification of the disease-causing clone is important to guide therapy. There is debate if bone marrow studies provide additional information in patients with negative SPEP/IFE and normal FLC ratio. The likelihood of finding BM abnormalities is very low in patients with negative serum immunofixation and normal FLC ratio. Still, we suggest to perform BM examination including flowcytometry in all patients with MGRS before starting therapy. This recommendation is based on the fact that MGRS are rare diseases, and that all efforts should be made for thorough evaluation of the patient. In patients with an identified bone marrow clone, we recommend additional evaluation according to hematologic guidelines. Depending on the nature of the underlying clone, and the isotype of the MIg, it should be considered to perform additional radiological, molecular, or cytogenetic studies according hematologic guidelines.

***In patients with MGRS we recommend treatment targeting blood pressure, proteinuria, and cholesterol, and life style measures as indicated for patients with CKD***

Proteinuria and eGFR are risk markers of progressive kidney disease. The KDIGO-CKD guidelines provide guidance for the management of patients with kidney injury and proteinuria. Although evidence in the MGRS population is lacking, it is unlikely that kidney injury in patients with MGRS will follow different rules. Therefore, most patients with MGRS should receive therapy as proposed in the CKD guidelines. This includes targeting low blood pressures, use of ACEi or ARB, statin therapy, and possibly novel anti-proteinuric therapies. Obviously, the treatment should be guided by patient characteristics: as an example, patients with amyloidosis often have low blood pressure and in these patients the use of ACEi or ARB is contra-indicated. Also, quality of life and life-expectancy should be considered.

***In patients with MGRS the choice of treatment should be guided by disease and patient specific characteristics. In patients with a documented bone marrow abnormality, and who are considered candidates for therapy, clone-directed hematologic therapy is preferred.***

Decisions regarding the use of hematologic therapy must take into account the clinical characteristics of the patient, the progressive nature of the underlying disease, and the side effects of therapy. Since in MGRS the MIg is responsible for causing kidney injury, it is logical to use treatment that targets the underlying clone. In the absence of a defined clone, empirical therapy is often used. Many different (combinations) of drugs are used in the treatment of patients with MGRS.

***We advise consultation with an expert center for all patients with MGRS.***

MGRS are very rare diseases. It is often difficult to establish a diagnosis and select the appropriate treatment. Patients with MGRS will benefit from consultation with expert centers where patients are counseled by a team consisting of nephrologists, hematologists, pathologists, with upon request added expertise from other specialties such as laboratory specialists, cardiologists, neurologists etc.

**Supportive evidence**

***We do not advise a routine kidney biopsy in patients with MGUS. A kidney biopsy should be considered if there is evidence of proteinuria, deterioration of eGFR, proximal tubular dysfunction (compatible with (in)complete Fanconi syndrome), or evidence of extrarenal manifestations that are suggestive of a MIg-associated disease (diarrhea, heart failure, neuropathy, low systolic BP, liver dysfunction).***

MGUS is prevalent. Kyle et al. documented an overall prevalence of 3.2% in the >50 yr population with males being more affected than females (3.7 vs 2.9%) (8). The prevalence increased with age: 1.7% in the 50-60 yr old, 3% in 60-70yr old, 4.6% in 70-80 yr old and 6.6% in those above 80 yrs. Using mass spectrometry, the prevalence is even higher, amounting 5.1% in the > 50 yr population (9). During follow-up of patients diagnosed with MGUS, the risk of progression to hematologic malignancy is approximately 1%/yr. The vast majority of patients with a diagnosis of MGUS will die from unrelated causes during follow-up. For comparison, the rate of death after 10, 20 and 25 yrs was 53%, 72% and 76% as compared to rate of progression to multiple myeloma of 6,10, and 11% respectively. MGUS guidelines define risk of progression to a hematologic malignancy based on M-protein level, type of monoclonal Ig, and FLC ratio(10, 11). Low risk is defined by a MIg of the IgG class, a serum MIg level <15 g/L, and normal к/λ ratio. In addition, a diagnosis of MGUS requires the absence of evidence of end-organ damage or myeloma defining events. A diagnosis of Multiple Myeloma is based on 1: ≥10% clonal bone marrow plasma cells or a biopsy-proven plasmacytoma plus evidence of one or more multiple myeloma defining events (MDE) related to the plasma cell proliferation, using CRAB criteria which include hypercalcemia, renal insufficiency, anemia, and bone lesions; 2. 60% or greater clonal plasma cells on bone marrow examination; 3. serum involved / uninvolved FLC ratio of 100 or greater (provided the absolute level of the involved light chain is at least 100 mg/L); 4. more than one focal lesion on MRI that is at least 5mm or greater in size (12). Of note, in the CRAB criteria a serum creatinine value of > 173 µmol/l (2 mg/dl) is used to define renal insufficiency. This represents an eGFR of 40 ml/min/1.73m^2^ in a 50 yr old male patient and 32 ml/min/1.73m^2^ in a 80yr old male patient. A recent review nicely summarizes current knowledge. (13). The authors explicitly mention that screening of the population for MGUS is not recommended and that patients with MGUS do not need treatment. They also provide guidance for the frequency of follow-up visits in patients with MGUS, according predicted risk of progression to myeloma, and as example low risk patients should be evaluated after six months, and at 1-3 yr interval thereafter.

Chronic kidney disease (CKD) defined as albuminuria (UACR > 30mg/day) or eGFR < 60 ml/min/1.73m^2^ is also prevalent. It is estimated that worldwide > 10% of persons will have micro-albuminuria or eGFR <60 ml/min/1.73m^2^.(14) The prevalence of CKD increases with age. In a Danish general population 5% of patients aged 40-69 yr and 46% of those aged > 70 yr had eGFR <60 ml/min/1.73m^2^.(15). In the US, CKD stage 3+4 were diagnosed in 0.7% of 20-39yr old and in 37.8% of the general population > 70 yr (16). In clinical practice these figures will even be higher, since patients who have visited a physician or the hospital do not reflect the normal healthy population. In a Dutch study, all available serum creatinine values of patients known in general practice were used to calculate eGFR using the MDRD formula. Approximately 7% of patients < 65 yr and 37% of patients >65 yr had eGFR < 60 ml/min/1.73m^2^ (17). Therefore, it is not unexpected that many patients with MGUS will have some evidence of kidney injury as defined by KDIGO-CKD criteria.

MGRS is rare in patients with MGUS and/or CKD. There are many studies that show that the risk of progressive kidney disease (and the prevalence of clinically relevant MGRS) is low in patients with MGUS.

Burwick et al. evaluated retrospectively the development of ESKD in patients followed at a VA Medical Center.(18). The study included 2,156,317 patients with known eGFR. Most patients hade normal eGFR. There were 283,988 patients with eGFR of 45-59 ml/min/1.73m^2^ (13%), 103,123 with eGFR of 30-44 ml/min/1.73m^2^ (4.8%) and 27,499 with eGFR of 15-29 ml/min/1.73m^2^ (1.2%). Overall, 21,898 patients had M-protein testing (1%). Patients with eGFR <60 ml/min/1.73m^2^ were more likely to be tested for a monoclonal protein (overall 2%), but not more likely to be positive. Median follow-up was more than 10 years (123 months). A positive M-protein test was not associated with development of ESKD in patients with eGFR 15-59 ml/min/1.73m^2^. Interestingly, a positive M-protein test was associated with higher rates of ESKD in patients with preserved eGFR, likely explained by patients diagnosed with multiple myeloma, in whom eGFR decreased from > 60 ml/min/1.73m^2^ to < 15 ml/min/1.73m^2^ within 10 years, or a progression rate of > 5 ml/min/1.73m^2^/yr.

Kristinsson (2009) evaluated 4,259 patients with MGUS diagnosed in Sweden in the period 1986-2005.(19) During follow-up 1,565 patients died. There was an excess mortality, especially in the elderly, however the mortality was mainly related to diabetes and hypertension. This suggests confounding by indication, i.e. there may have been a reason to assess the presence of a MIg. Overall, 259 patients with a diagnosis of MGUS died due to a hematologic malignancy. In contrast, only 42 patients had kidney disease reported as cause of death (< 1%).

Paueksakon (2003) reported data from a retrospective US study of 4,682 native kidney biopsies. (20). They selected patients with either a MIg in serum or urine and/or patients with a kidney biopsy diagnosis of MIg-associated kidney disease (the authors included cast nephropathy; PGNMID and C3GN were not considered). In total 121 patients fulfilled the inclusion criteria. In only 87 patients a serum or urine monoclonal immunoglobulin was detected (i.e. patients with ‘MGUS’”). Of these, 32 patients had a paraprotein-related kidney disease. In the other 55 patients other diagnoses were made such as diabetic nephropathy, hypertensive GN etc. Thus, in biopsied patients with known ‘MGUS’ and kidney injury a diagnosis of MGRS was made in 37%. On the other hand, a M-protein is not always detected in patients with diagnosed MGRS. In this study, a diagnosis of monoclonal immunoglobulin associated kidney disease was made in 66 patients, whereas a serum or urine M-protein were detected in only 32 (48%). Of note, FLC assay was not available at the time. The overall prevalence of “MGRS” in native kidney biopsies was low at 66/4,682 (1.4%). The diagnoses included cryoglobulinemic glomerulonephritis (n=20), LCDD (n=14), LHCDD (n=5), AL amyloidosis (n=13) and cast nephropathy (n=13).(the latter is formally not included in MGRS).

A study from Poland confirmed the low prevalence rate of MGRS in elderly patients.(21) This study evaluated the clinical diagnoses of 352 patients aged > 65 yr who were biopsied because of kidney injury (mean eGFR 39 ml/min/1.73m^2^; 55.6% nephrotic). Only 3.9% of patients had proven MGRS (mainly AL amyloidosis, MIDD). The number of patients with other forms of MGRS was not specified, however the total number of patients with “unspecified” MPGN was 3.1%. It is likely that some of these may have had MGRS (PGNMID, C3GN). Still, even in the elderly population the prevalence of MGRS is only 5% of all biopsied patients.

A study from the Mayo clinic confirmed the low biopsy and MGRS diagnosis rate in patients with CKD. (22).In this analysis of 101047 patients with CKD, only 2167 (2.1%) were tested for an M-protein at the time of CKD diagnosis. A kidney biopsy was done in 213 patients, and in 59 a diagnosis of MGRS was made (MGRS diagnostic rate 0.058% of all CKD, 3.85% of patients with CKD tested for an M-protein, 8.3% of patients with CKD who tested positive for an M-protein). Kidney biopsy rate was 17.5% in patients with CKD and MGUS, and 10.7% in patients with CKD and negative M-protein. This study thus illustrates that a kidney biopsy is only performed in selected patients with CKD and a documented MIg. In a separate study, the authors evaluated factors associated with the likelihood of performing a kidney biopsy in patients with a monoclonal gammopathy and CKD, diagnosed in 2017-2018,. There were 1608 patients with “MGUS”, and of these 596 patients had CKD (37.1%), illustrating that MGUS and CKD often coincide. A kidney biopsy was only done in 62 patients (10.4%). In 70 patients a kidney biopsy was not needed nor justified, since a diagnosis was already known (n=62, with AL amyloidosis in all but one) or patients would not tolerate treatment (n=8). The majority of patients were not biopsied, either because they were never seen by a nephrologist or hematologist (n=180), the presence of CKD or an M-protein was not known (n=106 and n=36 respectively), MGRS was considered unlikely (n=132 patients with stable eGFR, low level proteinuria, another likely cause of CKD), or possible but kidney biopsy postponed while waiting for evidence of progression (n=10). It is evident that the patient characteristics and the clinical course influenced decision making. Indeed, patients who were biopsied were younger (66 vs 71 years), had more severe proteinuria (1.67 vs 0.67 g/day) and higher serum creatinine (2.2 mg/dl vs 1.6 mg/dl). The patients who were biopsied had MGRS in 40% of cases, illustrating the selection bias.

***We do not advise routine screening of serum or urine for the presence of a MIg in patients with UPCR <1g/g (proteinuria < 1g/day) and/or eGFR >60 ml/min/1.73m^2^ (or >45 ml/min/1.73m^2^ in the elderly aged > 70 years). Prebiopsy screening of serum and urine for the presence of a MIg is not evidence-based but can be considered. There is variation between country-specific guidelines.***

Routine screening of serum in patients with CKD is debated. The KDIGO glomerular disease guideline briefly mentions MGRS, however the advise to perform full hematological evaluation is limited to patients with biopsy proven MGRS. (23). KDIGO does not address prebiopsy assessment. The British society of haematology advise M-protein testing and FLC assay in all patients with CKD referred to nephrology. (1). Unfortunately, this statement is not supported with literature references. Similarly, prebiopsy testing is not clearly discussed in the seminal paper of the International Kidney Monoclonal Gammopathy work group, although from reading between the lines one might conclude that IKMG also prefers routine screening.(3). Here also no supportive data is provided. We suggest that most studies support a strategy of restricted M-protein testing in patients with CKD.

Mendu et al. studied 1,487 patients referred for CKD management.(24) Median age was 70 yr. There were 61.4 % males. Only 12.3% of patients had eGFR > 60 ml/min/1.73m^2^. SPEP was done in 1,012 patients (68%) and was abnormal in 84 (5.6%). The result of SPEP affected diagnosis or management in only 22 patients, and all but one of these had a history of monoclonal disease, anemia, hypercalcemia, or severe CKD (defined as KDIGO very high risk i.e. CKD 4 or CKD 3b and ACR > 3 mg/mmol, OR CKD 3a AND ACR > 30 mg/mmol). Of note, these patients all had eGFR < 45 ml/min/1.73m2 or proteinuria > 1 g/day. A kidney biopsy was done in only 70 patients. A diagnosis of monoclonal disease, MPGN, or cryoglobulinemic-glomerulonephritis was made in 6 patients. In addition, a clinical diagnosis of myeloma related CKD was made in another 8 patients, and of amyloidosis in 1. Oncology referral, as proxy for possible MGRS, was done in 17 patients. In a companion study in this cohort, FLC testing was done in 374 patients (25%) (25). Although FLC was considered abnormal in approximately half of the tested patients, diagnosis was affected in only 5 and management in only 8 cases, respectively. The high number of abnormal tests likely reflects the effect of eGFR on serum FLC, reflecting the uncertainty of FLC interpretation in patients with reduced eGFR.

Chew et al. performed SPEP/UPEP or both in 165 patients with proteinuria > 3 g/day (26). A monoclonal spike was found in 19 patients. In only three a diagnosis of multiple myeloma (n=2) or amyloidosis (n=1) was made, the other 16 patients were diagnosed with MGUS. In 10 patients proteinuria/low eGFR were attributed to diabetes, hypertension, or crescentic glomerulonephritis. In 2 patients without a detectable MIg a diagnosis of AL-amyloidosis was made.

Koo et al. studied 1,591 adult patients who were biopsied.(27) In 943 patients, one or more tests were done to evaluate the presence of a monoclonal Ig (SPEP, sIFE, UPEP, uIFE or serum FLC). There were 303 patients with nephrotic syndrome, 249 with hematuria or non-nephrotic proteinuria, 331 with CKD and reduced eGFR, and 60 patients with AKI. Patients with a diagnosis of MM were included, and amounted 17 (of 303), 2 (of 249), 17 (of 331), and 1 (of 60), respectively. In the subgroups there were 17 (of 303), 6 (of 249), 3 (of 331), and 0 (of 60) patients with a diagnosis of MGRS. Most cases of MGRS were amyloidosis (21/26). The study is biased since M-protein testing was not done per protocol, thus SPEP was done in 832 patients (a plasma cell dyscrasia was found in 7%). The likelihood of diagnosing a plasma cell disorder was higher (11-20%) when using SIFE, UIFE or FLC assay, however these tests were done in a lower number of patients (311-551), indicating that these test were done for cause. The PPV of SPEP for diagnosing a plasma cell disorder was only 56%, and although the NPV is high at 97%, this is not very relevant in view of the fact that 93.5% of patients had no plasma cell disorder. The accuracy of the testing would even be more limited if patients with multiple myeloma had been excluded.

A recent study argued in favor of routine evaluation of patients with CKD for the presence of an M-protein or abnormal FLC ratio. (28). The authors present the “Mayo MGRS prediction tool”, a webbased calculator based on a model to predict the probability of MGRS in patients with CKD. The authors suggest that the tool could help to guide decision toward a kidney biopsy in patients with CKD. The study population included selected patients with CKD who had a monoclonal gammopathy, defined by a positive SPEP/SIFE or an abnormal FLC ratio, and available kidney biopsy. Data of 280 patients were used. The final model included 8 predictors: proteinuria, hematuria, systolic blood pressure, serum creatinine, diabetes, serum C3 level, positive urine M-protein, and affected/unaffected FLC ratio. The model performed well: using a threshold probability of > 0.25 (this means that a diagnosis of MGRS will be made in one of four biopsied patients), sensitivity was 88%, specificity 70.2%, and positive predictive value (PPV) 59.1%. Unfortunately, this study has many limitations, and firm conclusions cannot be drawn. An accompanying editorial comments summarizes the limitations. (29). Most importantly, there were many missing data (FLC ratio missing in 5%, complement C3 missing in 45%). The study only included patients with positive monoclonal gammopathy. A diagnosis of MGRS was made in 92 (32.9%) of biopsies, with AL amyloidosis present in 38 (41.3%). Patients with MGRS had more often positive urine M protein (82% vs 49%), and more often abnormal FLC ratio (70% vs 35%). Importantly, the authors did not provide data on how the decision to measure M protein and to perform a kidney biopsy was made. There is a major risk of confounding by indication. Indeed, in the abovementioned study of patients with CKD it was shown that in their practice only 2% of patients with CKD were tested for the presence of an M-protein, and less than 20% of patients with a positive M-protein were biopsied. Proper selection explains the high incidence of MGRS in the biopsied cohort. This explains the high PPV rate. Interestingly, the model did not include age, which further reflects bias, compatible with the common practice that a search for a monoclonal gammopathy is more often done in elderly patients.

There are other studies that often are quoted in support of routine testing of M-protein and FLC in patients with CKD. These are summarized below. While these studies suggest that an abnormal FLC increases the likelihood of finding a MGRS lesion in a kidney biopsy, these studies are all biased, with patient selection being a major flaw. When reading the study data, it should be realized that CKD and MGUS are common, whereas MGRS are rare diseases. Thus whereas MGUS is diagnosed in 3-5% of patients aged > 50 years, MGRS is diagnosed in approximately 20 per million/year. Studies from the US, Europe, Australia, Japan and India have reported that a diagnosis of MGRS is made in 1-2% of native kidney biopsies, with AL-amyloidosis being most prevalent (60-85%).(30, 31) (32-34)

In a Chinese cohort, older age, proteinuria >1.5g/day and an abnormal FLC ratio were independently associated with MGRS (35). In this study, 4604 patients with an M-protein were evaluated. Overall, 75 patients were excluded because of a hematologic malignancy. A kidney biopsy was done in 687 individual patients (15.1%) and in 261 (38%) a diagnosis of MGRS was made. AL-amyloidosis was the most frequent subtype of MGRS, accounting for 63% of cases. Patients were characterized by severe proteinuria (median 4.9 g/day). In this study hematuria, which is less prevalent in amyloidosis, was observed less frequently in the MGRS subgroup. In multivariable analysis, older age, proteinuria > 1.5g/day, and abnormal FLC ratio were associated with MGRS. Although MGRS patients had less often hematuria, diabetes, or hypertension these markers were not independent predictors. The study allowed to analyze accuracy of an abnormal FLC ratio, although this biomarker was available in a limited number of patients (MGRS 103/261; non MGRS 132/426). Specificity was calculated at 78%, sensitivity at 64%, PPV at 69%. Clearly, this is not the accuracy which would be required for patient care.

A similar study from China was recently reported. Dong et al. developed a nomogram model for predicting MGRS.(36) This study included 347 patients with a MIg, no hematologic malignancy, and a kidney biopsy (period 2018-2022). In 116 patients a diagnosis of MGRS was made (33%) Determinants of MGRS were older age, abnormal FLC, and absence of hematuria. The score predicted MGRS with an AUC of 0.85-0.88. However, this study clearly illustrates the bias of studies that tried to develop predictive models for the diagnosis of MGRS. First, there is biopsy bias. It is not mentioned how many patients with MGUS were identified, and how a decision was made toward biopsy. Clearly, the prevalence of MGRS in this biopsied cohort is much higher than expected, at 33%. Second, the distribution of subtypes of MGRS is notable: in this study 74% of patients were diagnosed with AL-amyloidosis and another 12 % with cast nephropathy (not an MGRS lesion), and 7.8% with LCDD. In accordance, the patients presented with overt proteinuria, often nephrotic syndrome (Uprot 5.5 g/day; serum albumin 27.8 g/l). The comparator cohort (MGUS, non-MGRS) also were often nephrotic and the most prevalent diagnosis was Membranous nephropathy. It is evident that these data cannot be used to decide upon the role of M-protein and/or FLC testing in patients with non-nephrotic CKD. The characteristics of the patient population likely explains that in this study absence of hematuria was associated with a higher likelihood of MGRS, in clear contrast to the findings of Klomjit et al (where the presence of hematuria was an independent predictor of MGRS)

In a s study from Japan proteinuria > 1.5 g/day, abnormal FLC ratio, and absence of diabetes were predictors of MGRS in multivariable analysis. (32)The authors evaluated 2972 kidney biopsies, in 166 patients an M-protein was found and in 44 patients a diagnosis of MGRS was made(1.5% of all biopsies, 45% of patients with CKD and MGUS).The authors acknowledge that the study is flawed by selection bias.

A study from Italy included data from 483 patients with biopsy proven kidney disease. (37). Most patients were screened for the presence of an M-protein, and a positive M-protein test was found in 63% of patients. A diagnosis of MGRS was made in 65% of the latter patients (or in 35% of all biopsies). A diagnosis of MGRS was more often associated with an abnormal FLC ratio. There were many missing data, and missingness was not at random. Importantly, the high prevalence of “MGUS” and “MGRS” is unexplained.

In another study form Italy, 25% of patients with a native kidney biopsy tested positive for an M-protein. (38). In approximately 50% of these patients a diagnosis of MGRS was made. We cannot explain these very high values, in fact even in patients with a non-MIg related kidney disease (such as membranous nephropathy) the prevalence of finding an MGUS was unusually high, e.g. 23% in patients aged 45-54 years, and 51% in patients > 75 years.

Taken altogether, the reported studies all suffer from bias and patient selection. The abovementioned study of Sy-Go et al. clearly argues against routine M-protein testing in patients with CKD. They identified from the patient records 101047 adult patients with CKD seen in the period 2013-2018. M-protein testing was done in 2167 patients (2%). After excluding 197 patients with ESRD (98 dialyzed, 99 transplanted) and 435 patients with no follow-up. 1535 patients remained. Of these 828 had no monoclonal protein, whereas 707 patients tested positive. A kidney biopsy was done in 89 M-protein negative patients, and in 124 of M-protein positive patients. A diagnosis of MGRS was made in 59 (42%) of the latter group. Patients with MGUS had no increased risk of kidney failure or death in adjusted analysis. Patients with MGRS had increased mortality risk, HR 1.75 (0.94-4.59) after adjustment for age, gender, eGFR and proteinuria) and HR 2.49 (1.35-4.59) after adjustment for Charlson comorbidity index. Of note, increased mortality was solely caused by the patients with AL-amyloidosis. It should be noted that the Charlson comorbidity index does not include amyloid involvement of the nervous or gastro-intestinal system (neuropathy, GE involvement etc). Thus, this study argues against routine testing,and indicates that targeted testing ensures a high diagnostic rate.

A study from the UK provided supportive information.(39) These authors analysed data of 3 UK cohorts of individuals with CKD. Most relevant were the data from the Renal Impairment in Secondary Care cohort. This study included patients with severe CKD, defined by either eGFR <30 ml/min/1.73m^2^ or eGFR 30-59 ml/min/1.73m^2^ AND either decline of eGFR > 5ml/min/yr or >10 ml/min/5yr OR UACR > 70 mg/mmol. Patients with a hematologic disorder were excluded. There were 878 patients, median age 65 years with median eGFR 31 ml/min/1.73m^2^ and ACR 33.4 (6.3-130) mg/mmol. In this population 77 individuals were diagnosed with an M-protein (8.8%) and 25 with a light chain only MGUS (2.8%). Participants with a monoclonal gammopathy were older (74 vs 64 years). Notably, the prevalence of MGUS was higher in patients with CKD, even adjusting for age. However, in multivariable analysis the presence of a monoclonal protein was not associated with kidney failure nor overall survival. This study provides further arguments to be restrictive with M-protein assays especially in the elderly (arbitrarily > 60-70 years) even if there is severe CKD.

***Adequate handling and interpretation of kidney biopsies is essential to diagnose (or exclude with certainty) MGRS. A detailed evaluation is required and includes light microscopy, immunofluorescence and/or immunohistochemistry, and electron microscopy studies. In some patients, additional studies should be considered.***

There are no controlled trials on the diagnostic performance of a kidney biopsy and the relevance of different techniques. Still, it is evident that histological evaluation is critical in diagnosing MGRS. A diagnosis of MGRS may be suspected on the basis of the light microscopy findings (e.g. amyloid, MIDD). However a definite diagnosis of MGRS usually requires the demonstration of monoclonal deposits by immunofluorescence (IF), defined as the deposition of a complete immunoglobulin with light chain restriction, or as the deposition of a single light chain and/or a single heavy chain. Therefore, all kidney biopsies should be evaluated by immunofluorescence (IF). The standard technique is IF on fresh frozen tissue, using specific antibodies directed against immunoglobulin heavy chains (IgG, IgA, IgM), light chains (κ/λ) and complement (C3, C1q). In patients with MGRS involving IgG, additional IgG subclass staining is preferred to demonstrate IgG subclass restriction. Of note, light chain staining can be false-negative or equivocal in some cases. l Consultation with laboratories with experience in IF may be warranted. It is important to realize that specificities of antibodies are different. Indeed, it has been shown (using immune-histochemistry) that the sensitivity to detect lambda light chains and thus diagnose AL-amyloidosis increases when using a panel of anti-λ antibodies.(40, 41) (42). These antibodies were often made “in house”, but are now commercially available. Immunofluorescence on fresh frozen tissue cannot always be used, e.g when fresh frozen, unfixed tissue is not available, or when the frozen part does not contain glomeruli. To overcome these problems, immunofluorescence techniques on formalin-fixed, paraffin embedded tissue using proteolytic enzymes for antigen retrieval have been developed (43). These techniques (IF-P; often using protease, proteinase XXIV, or proteinase-K) can be used as “salvage” techniques. Importantly, the use of IF-P is associated with lower sensitivity, therefore whenever possible, immunofluorescence on frozen tissue is preferred. There is one exception, and IF-P has been promoted to discover “hidden” monoclonal immunoglobulins. It is well known that the standard IF-F technique does not work well in biopsies characterized by the presence of crystalline deposits such as observed in LCPT. The crystalline structures likely contain masked epitopes, prohibiting binding of the antibodies. Antigen retrieval using IF-P allows staining of the relevant light chain. Most recently the IF-P technique was also proposed to allow unmasking of monoclonal immunoglobulins in other subtypes of MGRS. In fact, several studies showed that IF-P allowed a diagnosis of PGNMID or monoclonal Ig related MN in patients characterized by MPGN or MN patterns of injury, with solely C3 staining (reviewed in (43)). Thus, it has been suggested to perform IF-P in patients with equivocal or C3-dominant staining pattern in routine IF-F, particularly in patients with a detectable serum monoclonal immunoglobulin or abnormal FLC. Table 1.3 illustrates potential situations where IF-P should be considered.

| Table 1.3 Indications for paraffin immunofluorescence |
| --- |
| Frozen tissue unavailable or insufficient |
| Suspected LCPT or crystalglobulin nephropathy |
| MPGN with negative staining for IgG and complement |
| C3GN associated with auto-immune disease or monoclonal Ig in serum |
| Membranous nephropathy with negative staining for IgG |
| Fibrillary GN with apparent monotypic deposits |

Adapted from (43)

Messias described the experience of the Nephropath Laboratory (currently Arkana) in the period 1 January 2013 through 30 september 2013.(44) Biopsies were transported in Michel’s medium. In this period 4969 kidney biopsies were examined, and IF-P was requested in 324 (6.5%). In the majority IF-P was used as “salvage” technique, in 97 cases (approximately 2% of all biopsies) IF-P was requested to evaluate the presence of masked immunoglobulins or light chains. In 36 cases IF-P was used to evaluate light chain associated diseases such as amyloidosis, LCDD, and LCPT. In only 2 cases (both LCPT) a contribution to diagnosis was made. In 61 cases, IF-P was used to evaluate immune complex glomerulonephritis. In 41 the diagnosis was not changed. However, IF-P was deemed necessary to reach the correct diagnosis in others: this included 7 patients with MN and negative (6) or very weak (1) IgG staining, 7 patients with MPGN staining positive for C3 positive but negative for IgG, and rare cases of pauci-immune crescentic glomerulonephritis or pauci-immune MPGN. In these patients IF-P unmasked a monoclonal immunoglobulin, most often of the IgGκ class. Notably, in all biopsies deposits were visible in EM.

Of note, the reports of the added value of pronase/proteinase digestion in glomerular diseases with non-crystalline C3 dominant deposits are from centers that use transport medium (Zeus, Michel’s) for kidney biopsies. The added value of IF-P has not been unequivocally documented in centers that perform IF on tissue that is directly frozen. The personal experience of the authors (with IF-F as standard technique) also suggests limited advantage of this novel technique in patients with non-crystalline, glomerular deposits.

An alternative for IF is immunoperoxidase-based immunohistochemistry on fixed tissue. Although the results of IHC are in general comparable to the findings in IF, in our view IHC is less sensitive due to the relative high background staining.

It is important to point out that the kidney biopsy will not demonstrate MIg deposits by IF when kidney injury is caused by an indirect mechanism. In these cases, suspicion for an MGRS-associated lesion should be raised by the combination of the pathological diagnosis (e.g. C3 glomerulonephritis), age and the presence of a detectable M-Ig in serum (see chapter IX)

A congo-red (amyloid) stain needs to be performed with a low threshold and is especially advised in all suspected cases of amyloidosis, i.e. in patients with a detectable MIg in the serum or urine, in patients with fibrillary deposits on EM, or in patients with nephrotic syndrome and normal glomeruli by light microscopy. It may be considered to evaluate congo-red staining with fluorescence microscopy, a technique which is more sensitive, in cases with high suspicion of amyloidosis especially with scant deposits in non-renal tissues.(see Chapter II) Staining for DNAJB9 is advised in patients with findings suggestive of fibrillary GN.(45). It is also useful in patients with fibrillary deposits by EM and negative/nonspecific IF findings, in order to differentiate fibrillary GN from other glomerular lesions, including amyloidosis. Rare cases of congo-red positive fibrillary GN can also be distinguished from amyloidosis using DNAJB9 staining (46). Additionally, DNAJB9 staining is helpful to differentiate fibrillary GN from AH or AHL amyloidosis.

Although not universally available, we suggest electron microscopy in all cases to evaluate the localization and structure of deposits. At least two glomeruli should be examined, as deposits can be scattered. Also, the proximal tubules and TBM should be critically evaluated for crystals or non-organized deposits. Electron microscopy is essential to confirm the organization of the deposits in AL amyloidosis, fibrillary GN, cryoglobulinemic GN and immunotactoid glomerulonephritis. In MIDD, typical powdery electron-dense deposits can be seen linearly along tubular basement membranes, often but not always accompanied by glomerular deposits. In a significant proportion of cases of cryoglobulinemic glomerulonephritis, (focal) deposits can show microtubular, cylindrical, or annular substructures.(47) The “fingerprint” substructure is typically seen in patients with lupus nephritis and cryoglobulinemia. (48) In PGNMID and C3 glomerulopathy, electron microscopy demonstrates non organized deposits. In centers where EM is not available, some MGRS-associated renal lesions can be diagnosed when the kidney biopsy is evaluated by an expert nephropathologist; a diagnosis of AL amyloidosis and fibrillary GN may be made when the typical light microscopy and IF features are present, and a positive staining with congo-red or DNAJB9 is found, respectively. A diagnosis of MIDD without EM could also be rendered when the typical LM and IF findings are found (see Chapter III). A diagnosis of C3 glomerulopathy can be based on the typical LM and IF findings alone, although the distinction between C3 glomerulonephritis and DDD requires ultrastructural examination. Also, EM findings may help to distinguish between C3GN per se and MPGN with masked immunoglobulin deposits: subepithelial humps, intramembranous deposits, or deposits with slight electron density favor C3GN, whereas predominant subendothelial deposits, or any organizational substructure favors MPGN with masked deposits (43).

Additional staining and/or mass spectrometric analysis of laser microdissected kidney tissue (LCMS) containing congo-red positive deposits to determine the type of protein involved in amyloidosis should be considered when the findings by IF and/or immunohistochemistry are equivocal (see Chapter II). Of note, rare cases of MGRS related to the deposition of monoclonal IgD or IgE will not be detected by routine IF and may require LCMS to determine the composition of the deposits. Royal et al. reported a 77yr old patient, who presented with nephrotic proteinuria and renal insufficiency (serum creatinine 194 umol/l). Notably, κ/λ ratio was abnormal (0.02, with a free λ concentration of 2303 mg/l) (49). Kidney biopsy revealed nodular glomerulosclerosis. Routine IF staining (IgG,IgA,IgM, κ, λ) was negative. Suspicion of MIDD was raised by EM which showed punctate powdery deposits in GBM and TBM. LC-MS/MS was done, and revealed IgD spectra, and IgD-HCDD was confirmed by IHC using anti IgD antibodies. The absence of epitopes that are recognized by the antibodies used in routine IF may also explain negative staining. In HCDD deposits usually contain a truncated immunoglobulin, explaining negative staining, as illustrated in a recent case report (50). In this case, a patient presented with deteriorating kidney function and moderate proteinuria. Laboratory studies revealed a monoclonal IgAκ band, and an increased κ/λ ratio (37). Bone marrow biopsy revealed 8% IgAκ restricted plasma cells. Kidney biopsy revealed nodular glomerulosclerosis. IF staining was negative (using two different anti IgA-antbodies). Again, suspicion of MIDD was raised by EM showing punctate powdery deposits in mesangium, TBM, and segmentally in the GBM. HCDD was proven by LCMS showing abundant spectra of the IgA constant region.

Since expertise is needed to diagnose MGRS, it is advised that biopsies of patients with suspected MGRS should be evaluated by expert renal pathologists with thorough knowledge and experience in the diagnosis of MGRS-related renal lesions. Although not studied in MGRS, it is likely that expert pathology review will contribute to the precise diagnosis, as has been demonstrated for lymphoma diagnosis in the French Nephropath Network. (51). minimum set of required investigations for the histopathological evaluation of a kidney biopsy is given in Table 1.4. Although not required, the pathologist may benefit from information on the presence of a MIg in serum at the time of the pathological study for optimal evaluation and correlation of the pathological diagnosis with the clinical findings. This is particularly relevant in centers where routine kidney biopsy evaluation does not include standard IF with κ/λ staining, nor standard EM.

Table1.4: kidney biopsy requirements

| Light microscopy | Staining: methenamine silver, PAS, hematoxylin and eosin, trichrome |
| --- | --- |
|  | On indication: congo-red, |
| Immunofluorescence on frozen tissue | Minimal staining: IgG, IgM, IgA, к, λ, C3, C1q |
|  | On indication: IgG subclasses; amyloid protein subtypes (see chapter 2) |
| Immunohistochemistry on paraffin | Staining as in IF; additional DNAJB9 staining |
| On indication: Immunofluorescence after pronase/proteinase digestion | - Fresh tissue unavailable, - suspected LCPT, LCCP, or crystalglobulin nephropathy - necessary for the evaluation of C3G or MPGN with negative IF in laboratories that use transport medium for kidney biopsies. - Membranous nephropathy with negative staining for IgG |
| Electronmicroscopy | - Preferred routinely in all glomerular diseases. - Essential for the diagnosis of immunotactoid glomerulopathy and DDD. - Relevant for the detection of organized deposits. - Advantageous for confirmation of fibrils, and localization of non-organized deposits. |

Note of caution: although light chain restriction is often used to define MGRS, it is important to realize that light chain restriction per se does not proof monoclonality. Therefore, it is suggested to use the term monotypic in cases with documented light chain restriction. Monoclonality (and thus MGRS) can be presumed when biopsy findings parallel the findings of a monoclonal gammopathy in serum, urine, or bone marrow. Definite proof of monoclonality would require more detailed and technically demanding studies such as finding unique sequences of the heavy and light chains variable domains. This difference between monotypic and monoclonal is not merely a theoretical exercise. Some patients with PGNMID and monotypic staining may not represent MGRS ( see chapter V PGNMID)

***We recommend the search for a MIg in serum and/or urine, with appropriately sensitive laboratory techniques, in patients with a histologically documented MGRS. A similar approach is recommended in patients with kidney injury patterns that are typically associated with a MIg (e.g. C3GN).***

The standard technique for MIg detection in serum and urine was electrophoresis and immunofixation. These techniques have limited sensitivity, e.g. SPEP and SIFE are not able to detect MIg in concentrations < 1g/l. The introduction of free light chain assays has increased the sensitivity of testing to diagnose disorders associated with a monoclonal immunoglobulin. The first available assay (Freelite^TM^) uses polyclonal sheep antibodies. The proposed reference values amounted 3-19 mg/l for κ light chains, and 6-26 mg/l for λ light chains with the κ/λ ratio ranging from 0.26-1.65 (52). Investigators have noted an upward drift of the κ levels, likely related to interference of the old calibrator. As a result, it was suggested that the diagnostic reference range should be adapted, in order to avoid “overdiagnosis”. (Table 1.5) (53) . It was suggested that in the serum κ light chains are present as monomers, whereas λ light chains are to a greater extent present as dimers (respective molecular weight 22.5 and 45 kDa) (54). In patients with renal insufficiency, serum levels of both free к and free λ chains increase. However, in view of the differences in molecular weight, κ light chains are more affected by a decrease in eGFR. As a result, in patients with renal failure, different cut-off values should be used for the к/λ ratio. Hutchison proposed a renal reference ratio of 0.35-3.10 (52). A population-based study from Iceland, that included more than 6500 participants with eGFR < 60 ml/min/1.73m^2^, with FLC measured by Freelite^TM^, and excluding participants who were tested positive for an M-protein by SPEP and SIFE, proposed new reference values (55). The authors confirmed that free κ and λ chains increased with decreasing eGFR, the effect being more notable for κ chains. As a result there was a negative correlation between the κ/λ ratio and eGFR. Table 1.5 ilustrates the proposed reference values for the κ/λ ratio:

Table 1.5: proposed reference values for κ/λ ratio in healthy persons and patients with CKD

|  | Lower Limit Normal | Upper Limit Normal |
| --- | --- | --- |
| **eGFR > 60 ml/min/1.73m^2^** |  |  |
| Age < 70 years | 0.44 | 2.16 |
| Age ≥ 70 years | 0.46 | 2.59 |
| **CKD** |  |  |
| eGFR 45-60 ml/min/1.73m^2^ | 0.46 | 2.62 |
| eGFR 30-45 ml/min/1.73m^2^ | 0.48 | 3.38 |
| eGFR < 30 ml/min/1.73m^2^ | 0.54 | 3.30 |

Adapted from (53, 55)

The Binding Site (manufacturer of the Freelite assay) has introduced a new calibrator, and further upward drift is no longer expected. Still, in a comparison of the most recent old and new calibrator, serum FLC levels and FLC ratio were comparable, suggesting that old reference values can no longer be used. We suggest to use the reference values as shown in table 1.5. When reading literature, be aware of the upward drift and the ensuing differences in FLC levels between the older and most recent studies.

In view of the high sensitivity of the FLC assay, the usefulness of urine IFE testing can be questioned. Based on kidney physiology, it is not expected that increased levels of urine MIg could appear in patients with absent MIg in serum and/or normal FLC ratio. Indeed, Katzmann et al. (56) evaluated 428 patients with a monoclonal Ig associated disease and a positive Urine IFE test (56). When combining results of SPEP, SIFE and FLC ratio only 2 patients would have been missed, and in both patients (diagnosed with MGUS) this was without clinical relevance. Others have reported similar conclusions (reviewed in (54)). Still, there are proponents of performing UIFE, pointing to the high sensitivity especially when using concentrated urine and in situations where the monoclonal free light chain may be overwhelmed by high polyclonal free light chain production (57). When evaluating the diagnostic accuracy of serum and urine immunofixation plus FLC measurement in 115 patients with proven AL-amyloidosis, the combination of serum IFE and FLC detected 96% of patients (110/115). Urine IFE was positive in all 5 negative patients, thus increasing sensitivity to 100% (58). Similarly, in another study positive urine IFE was observed in approximately 1.5% of patients with negative serum SPEP/SIFE and normal FLC ratio. (59). A similar conclusion was reached in a recent abstract (Nazzal ASH 2024), based on a meta-analysis of published data: the pooled sensitivity in AL-amyloidosis was 72.4% for SPEP/SIFE, 97.7% for SIFE + FLC ratio, and 99% for SIFE, UIFE, + FLC ratio, indicating that UIFE increased sensitivity by 1.3%. Notably, the absence of a MIg in urine is a criterium to evaluate hematologic complete response in MIg-amyloidosis. (60) Thus, urine IFE should be performed in patients with (suspected) MGRS and normal serum IFE and FLC ratio. Of note, since free light chains are reabsorbed by the proximal tubules, proximal tubular dysfunction will affect free light chain excretion. There is insufficient date to allow firm conclusions, however we caution against building firm conclusions on the presence of very low amounts of urine free light chains (<100 mg/l) in patients with kidney damage and documented proximal tubular injury.

In 2011 a novel assay for FLC detection was introduced. This N-latex assay is based on monoclonal antibodies (61, 62). Reference values are 7-22 mg/l for κ light chains, 8-27 mg/l for λ light chains, with the κ/λ ratio ranging from 0.31-1.56 . Although the Freelite^TM^ assay (Binding Site) and the N-Latex assay (Siemens) have similar diagnostic accuracy in patients with normal kidney function, there is emerging evidence that these assays cannot be used interchangeably, in particular in patients with kidney dysfunction. Kennard studied 105 patients treated with hemodialysis (patients with abnormal M-protein were excluded) (63). As expected κ and λ light chains were increased. However, there was a marked difference between the assays. In particular, λ light chains levels were lower when using the Freelite^TM^ as compared to the N-latex assay . As a result, using the Freelite^TM^ assay the κ/λ ratio was higher in dialysis patients and exceeded the normal reference range. When using the renal reference range all but one patient had normal values. In contrast, the N-latex assay provided κ/λ ratio’s within the range of healthy individuals. The authors also analyzed dialyzer clearance: there was a 2/3 reduction of κ light chains independent of the assay used. However, λ light chains were reduced 2/3 when using N-latex assay, but only 1/3 when using the Freelite^TM^ assay. From a physiologic perspective, and assuming that λ  light chains are present as dimers, less reduction of λ over κ would be expected. An explanation for these findings is lacking. A recent study extended these observations to patients with a wide range of eGFR. Sprangers et al. included 477 patients with CKD (64). In this study, κ FLC were higher by Freelite^TM^ and λ FLC were higher by N-Latex. In both assays, both κ and λ FLC correlated inversely with eGFR, but this effect was more pronounced in λ-FLC measurement by N Latex. Consequently, although the κ/λ ratio by Freelite^TM^ was inversely correlated by eGFR, the κ/λ ratio by N Latex was positively correlated with eGFR.

Meanwhile other commercial assays are available such as Kloneus^TM^ (Trimero diagnostics), Diazyme free light chain assay, Sebia FLC ELISA’s and Seralite^TM^ (65). Although compararative studies are limited, it is evident that these assays cannot be used interchangeably in patients with CKD. From a clinical perspective it is important to know that assays are different, and the same assay should be used consistently in the follow-up of patients. For diagnostic purposes, in patients with kidney disease the κ/λ chain ratio should take into account the actual eGFR. The use of different cut-offs values for the к/λ ratio is appropriate when using the Freelite^TM^ assay. Of note, hematologic response to therapy is based on (changes and/or disappearance) of M-protein levels by SIFE, UIFE, and changes in the measured FLC’s (studies have evaluated absolute levels of the individual light chains, as well as the ratio, and the difference between the involved and uninvolved light chain (dFLC). Since kidney failure will not only affect the absolute levels but also the FLC ratio and the dFLC, interpretation of response criteria must be done cautiously. In general in patients with severe kidney failure (low eGFR) dFLC will remain high, and as a consequence hematologic response will be underestimated. Literature data is limited. Cohen et al. cautioned against the use of (d)FLC.(66). Values of dFLC <10mg/L or a concentration of involved FLC <20mg/L were not observed pretransplantation. They also evaluated hematologic response using laboratory data obtained at the time of renal transplantation and at re-assessment immediately post-transplant: CR rate were 60.0% and 67.5%, VGPR 15.0% and 20.0%, PR 15.0% and 5.0% and NR 7.5% and 2.5%, respectively. Based on post-renal transplant sFLC measurements, 7 patients (17.5%) had their hematologic response re-classified, of which 6 were assigned an improved response category including two patients who had been classified as non-responders pre-transplantation. Notably, evaluation of urine may also pose problems, either because of anuria and inability to perform immunofixation electrophoresis of urine, or because of aberrant FLC excretion due to tubular dysfunction.(52, 67). In another study Heaney et al. measured serum FLC using Seralite^TM^ in patients with non-myeloma associated AKI (68). Patients with AKI showed increased levels of both κ and λ chains compared to controls, with increases in λ surpassing increases in κ. As a result, the ratio κ/λ was below the normal range (0.5-2.5) in 89% of patients with AKI. Moreover, dFLC was elevated (median 57 mg/l (range 0-376 mg/l) and the authors proposed a cutoff of 399 mg/l to differentiate non-myeloma AKI from myeloma associated AKI. Results will be different when using other assays.

***In patients with documented MGRS evaluation should include bone marrow examination including flowcytometry.***

Bone marrow examination (aspirate and biopsy) to identify the responsible BM clone is standard practice in patients with MGRS and an abnormal M-protein or FLC ratio. Flow cytometry allows the detection of small clones. There are few studies that evaluated the added value of BM investigation in patients with normal SPEP/IFE and normal FLC ratio. Bhutani *et al.* evaluated the accuracy of hematologic testing in patients with PGNMID.(69). This study included 60 patients, mean eGFR 36 ml/min/1.73m^2^, and proteinuria 3.6 g/day. Serum immunofixation was positive in 12 patients, in 4 confirmed by abnormal к/λ ratio. In addition, there were 8 patients with an abnormal FLC ratio in the absence of an M-protein. In 40 patients bone marrow was evaluated, including flowcytometry. Abnormal BM findings were found in 100% of patients with abnormal SPEP and FLC ratio (3/3), in 75% of patients with abnormal SPEP and normal FLC ratio (6/8), and in 16% of patients with abnormal FLC ratio only (1/6) and in none of the patients with normal SPEP and FLC ratio (0/23). This study thus suggests that in patients with PGNMID, negative serum and urine immunofixation and normal FLC ratio the value of BM studies is negligible. Of note, there is an interesting observation in this study; the authors have used the normal upper cut-off value of 1.65 to define an abnormal FLC ratio. If they used the cut-off value of 3.1 (which was advised in patients with severe kidney failure) only 1 patient had an abnormal FLC ratio. In all patients with a FLC ratio between 1.65 and 3.1 an IgGκ was found. This is compatible with the idea that κ light chains are more affected by kidney function. There are reports of rare patients with PGNMID in whom a BM clone was found despite normal serum/urine evaluation (admittedly, it is not always clear if in these studies FLC were measured, and how sensitive the SPEP/IFE was.The study of Bhutani et al. should be interpreted with caution, since it is now argued that PGNMID in many patients may not represent a monoclonal disorder (see Chapter 5). To the best of our knowledge there are no such studies in patients with other forms of MGRS disease entities. In view of the rarity of MGRS, and the uncertainty surrounding PGNMID, we suggest that BM evaluation be considered in all patients with suspected MGRS who are considered for hematological therapy.

In patients with a plasmocytic clone additional genetic PCR testing and fluorescent in situ hybridization enable detection of aberrations that may inform treatment and prognosis. Examples are the t(11;14) translocation, (patients will respond to venetoclax), gain of chromosome 1q21, or the presence of the MYD88 mutation. (70-72). We refer to recent hematologic reviews for relevant information. (73-75). Therefore, when perfoming a bone marrow biopsy it seems a good idea to harvest a tube for iFISH, thus avoiding a second bone marrow exam.

Some centers advise flow cytometry of peripheral blood in patients with proven MGRS, and negative BM examination. In rare patients an abnormal clone of lymphocytic cells can be found in peripheral blood using flowcytometry. If the detected clone is of similar subclass/light chain as the causative MIg, than this likely can be used to guide treatment and as marker during follow-up. There is no evidence that this approach is cost-effective, and improves management.

It is expected that in the near future novel techniques will be introduced in clinical practice that are more sensitive in detecting low levels of serum MIg, or the presence of very small bone marrow clones. Examples are mass spectrometry, RNA sequencing, and PCR techniques. (76, 77). These techniques are important in detecting minimal residual disease in patients treated for multiple myeloma. Research is necessary to evaluate the value of these techniques in patients with MGRS, since more sensitive techniques might cause benefit (preventing undertreatment) and harm (causing overtreatment).

***In patients with MGRS we recommend treatment targeting blood pressure, proteinuria, and cholesterol, and life style measures as indicated for patients with CKD***

There are no data of conservative therapy in patients with MGRS. However, it is expected that blood pressure, lipids, and life style affect kidney injury progression and cardiovascular risk in patients with MGRS as in patients with CKD of other causes. Therefore patients with MGRS should receive treatment and advice according the KDIGO-CKD guideline.(78)

***In patients with MGRS the choice of therapy should be guided by disease and patient specific characteristics. In patients with a documented bone marrow abnormality, and who are considered candidates for therapy, clone-directed hematologic therapy is preferred.***

Treatment of patients with AL-amyloidosis is based on evidence from RTC’s (see Chapter II). There is no evidence from RCT’s to guide management in patients with other MGRS subtypes. In MGRS, treatment decisions require balancing benefits and risks, adapting treatment to the needs and expectations of the patient. The decision to start therapy and the choice of therapy is dependent on patient characteristics, the underlying disease, the course or proteinuria and eGFR, and patient and physician preference. A wait and see-policy may be preferred in patients with MGRS who are old, have clinically relevant comorbidities, or low performance status and low life expectancy. Of note, few patients will die from a hematologic malignancy, thus treatment is directed at preventing renal and extrarenal complications. The approach towards treatment is different for the types of MGRS that are associated with a risk for extrarenal (cardiac) disease. In these subtypes a complete or very good partial hematologic response is necessary to prevent progressive heart and kidney failure. In contrast, in patients with renal-limited MGRS, where renal outcome is the only relevant parameter, even a partial response to therapy may be sufficient to stabilize eGFR, and reduce proteinuria. In general renal response follows the hematologic response. However, in patients with low eGFR or severe proteinuria at start of therapy, a “point of no return” may have been reached, with kidney disease progression occurring despite a hematologic response. An overarching algorithm of management of patients with non-AL-Amyloid MGRS and an overview of the relevant questions that guide decision making are provided in Figures 1 and Box 1. Treatment of AL-amyloidosis is discussed in Chapter II. Non-amyloid MGRS subtype specific information is given in the chapters III-IX. The management of patients that are prepared for kidney transplantation deserves special attention. These are discussed in Chapter X.

As a practice point we here provide an overview of the most commonly used drugs (Table 1.6)

The table provides information on drug toxicity, and also provides pharmacokinetic information that is relevant for the use of these agents in patients with reduced GFR or patients with ESKD. Special considerations relevant for the care of patients who have received a kidney transplantation are mentioned in chapter X.

Table 1.6 drugs used in the treatment of patients with MGRS

| **Product** | **Target**  Bcell plasma cell | **Dose reduction in CKD** | **Renal toxicity** | **Common side effects** |
| --- | --- | --- | --- | --- |
| **Proteasome inhibitors**  Bortezomib  Carfilzomib | +  + | No  Yes | No  AKI, TMA | Thrombocytopenia, polyneuropathy, VZV  Thrombocytopenia, HSV/VZV, Cardiac toxicity |
| **Monoclonal antibodies**  Rituximab  Obinutuzumab  Daratumumab  Isatuximab | +  +    +  + | No  No  No  No | No  No  No  No | Infusion reaction, HBV reactivation  Infusion reaction, HBV reactivation  Infusion reaction, infections  Infusion reaction, infections |
| **Cytostatics**  Cyclophosphamide  Melphalan  Bendamustine  Venetoclax (for t(11;14) | + +  +  + +  + + | yes  Yes  Yes  No | No  No  No  Yes | All: infections  Nausea, cytopenia  Cytopenia  Cytopenia  Cytopenia, Nausea, Diarrhoea, Fatigue |
| **IMiDs**  Lenalidomide  Pomalidomide | + +    + | Yes  Unknown | AKI  No | Teratogenicity, cytopenia, DVT  increase NTproBNP  Teratogenicity, cytopenia, increase NTproBNP |
| **Others**  Fludarabine  Ibrutinib | +  + | Yes  Unknown | No  ↑ sCr | Cytopenia, hemolytic anemia, neuropathy  Cardiac toxicity; bleedings |

Adapted from (79)

***We advise consultation with an expert center for all patients with MGRS.***

Since MGRS are very rare diseases, and it is often difficult to establish a diagnosis and select the appropriate treatment, consultation with expert centers where patients are counseled by a team consisting of nephrologists, hematologists, pathologists, and other specialists (e.g. laboratory specialist, cardiologists, neurologists) is advised. Although not studied in MGRS, in other rare kidney diseases early referral to or consultation of an expert center was important to establish early a correct diagnosis. (80) The prevalence of MGRS in a population is not well defined. Khera et al. retrospectively recruited patients with MGRS, excluding patients with AL-amyloidosis, diagnosed in the period 2004-2017, in 5 Centers located in the UK and Ireland.(81) In total, 41 patients were diagnosed in this period, i.e. < 1 patient with MGRS per center per year. The most common diagnosis was LCDD, being present in 61% of patients (Table 1.7).

Mancuso et al. analysed data from Italy.(82) The authors included patients with renal limited MGRS, thus excluding patients with AL-amyloidosis and multi-organ involvement. In a period of 15 years (2006-2021) 60 patients were diagnosed in 8 Italian centers. The majority of these (70%) had AL-amyloidosis. The incidence thus translates to 2 patients with AL-amyloidosis and 1 patient with another subtype of MGRS per 5 years. Gozzetti et al. performed a retrospective survey, amongst 19 centers in 12 countries, covering the period from 2003-2020. (83). The survey included patients with amyloidosis. In total 280 patients were included, the majority (180 or 64%) with a diagnosis of amyloidosis. Thus, 100 patients had non-amyloid MGRS, which amounts 0.33 patient per center per year. Also in this survey LCDD was the most prevalent non-amyloidosis MGRS subtype (Table 1.7)

Table 1.7 epidemiology of MGRS

|  | Khera 2019  N=41  (81) | Mancuso 2024  N=60  (82) | Gozzetti 2020  N=280  (83) |
| --- | --- | --- | --- |
| Period | 2004-2017 | 2006-2021 | 2003-2020 |
| Centers | 5 | 8 | 19 |
| Countries | 2 (UK, Ireland) | 1 (Italy) | 12 |
| Diagnosis |  |  |  |
| Amyloidosis | Excluded | 42 (70%) | 180 |
| LCDD | 25 (61%) | 7 (12%)* | 53 (53%)* |
| LHCDD | 2 (4.9%) |  | - |
| PGNMID | 3 (7.3%) | 2 (3.3%) | 14 (14%) |
| Immunotactoid | 1 (2.4%) |  | 4 (4%) |
| LCPT | 6 (14.6%) | 5 (8%) | 11 (11%) |
| C3GN | 2 (4.9%) | 1 (1.7%) | 7 (7%) |
| TMA | 1 (2.4%) |  |  |
| Other |  |  | 5 (5%) |
| Monoclonal fibrillary GN |  | 2 (3.3%) | 4 (4%) |
| Cryoglobulinemic GN |  | 1 (1.7%) | 2 (2%) |

*percentage of non-amyloid MGRS. In Mancuso and Gozetti MIDD is used, no differentiation between LCDD, HCDD, LHCDD

**Chapter 2: Monoclonal Immunoglobulin associated amyloidosis (AL, AH, AL/AH)**

**Introduction**

Amyloidosis is a term to describe a group of disorders with organ damage caused by extracellular fibrillary deposits. These fibrils typically originate from proteins or degraded protein fragments that misfold and aggregate in a β sheet structure (84-86). Monoclonal immunoglobulin-associated amyloidosis, where the fibril formation is caused by an abnormal “amyloidogenic” MIg (fragment), is considered part of the spectrum of MGRS. In MIg-associated amyloidosis, the amyloid precursor protein can be derived from a light chain (AL amyloidosis), and less frequently from a heavy chain (AH amyloidosis) or both (AL/AH Amyloidosis). Although AL-amyloidosis is the most frequent form, and the evidence from the literature is mainly based on studies in patients with AL-amyloidosis, we will in this chapter use the overarching term monoclonal-immunoglobulin-associated amyloidosis (MIg-amyloidosis).

There are many other types of amyloidosis, and we provide a brief overview to enable a differential diagnosis. A recent overview on amyloidosis nomenclature mentions 42 proteins that can form amyloid fibrils (87). The organ involvement of these amyloid fibrils is quite variable; many form only localized deposits, whereas others cause systemic disease with involvement of different organs or the nervous system. Some forms are hereditary (due to mutations in the genes that encode the proteins) whereas others are acquired.

It is important to know the physiology (or kinetics) of amyloid fibril formation. The process involves the production of misfolded proteins, that are present as protofilaments, in a beta-sheet structure. These protofilaments form fibril monomers, which then combine to form dimers, tetramers, octamers etc. Finally, a larger aggregate is formed, the so-called nucleus. This nucleus then acquires more monomers to further elongate into fibrils. The concentration of monomers needed to elongate the nucleus to fibrils is 10-20-fold lower than the concentration needed to form dimers or tetramers and the process of fibril elongation is exponential. This physiological mechanism might explain why early treatment is more successful than late treatment.

Amyloid deposits not only contain the specific amyloidogenic protein, but also components that are present in most amyloid tissue. These are called “amyloid signature proteins”, examples are serum amyloid P, heparan-sulfate proteoglycan, Apolipoprotein AIV, and apolipoprotein E; hence these proteins are important in verifying a proteome as derived from an amyloid deposit in mass spectrometric typing of amyloidosis.

It is suggested that the cell or tissue injury in amyloidosis might be explained by two separate processes: 1. cell toxicity induced by soluble prefibrillar aggregates and 2. tissue toxicity by deposition of the large fibrillar aggregates. According this theory, treatment might limit or stop injury (by inhibiting the formation of the prefibrillar aggregates) despite the persistence of the amyloid fibril deposits.

Interestingly, the various amyloid subtypes have different organ distribution. This “organ tropism” is unexplained, but local fibril formation is likely dependent on the amyloidogenic protein, other constituents of amyloid (Serum amyloid P protein, ApoAIV, ApoE) and the composition of the organ tissue, with an important role for the proteoglycan constitution. Thus, not all forms of amyloidosis involve the kidney. Table 2.1 provides an overview of the proteins involved in the different forms of amyloidosis with kidney involvement. Some forms of the systemic amyloidosis predominantly affect the kidney (ALECT2, AFib, ALys, AApoAI, AApoAII, AApoAIV, AApoCII, AApoCIII), whereas other forms of systemic amyloidosis can affect all organs to a variable degree, and morbidity is often related to extrarenal involvement (e.g. heart involvement in AL amyloidosis). Some clinical features are typically seen in AL amyloidosis (macroglossia, peri-orbital hematoma; factor X deficiency). Transthyretin (ATTR) amyloidosis mainly affects the heart and nervous system, and only vary rarely the kidney.(88)

Table 2.1: precursor proteins in renal amyloidosis

| Protein | Amyloid nomenclature | Hereditary/Acquired |
| --- | --- | --- |
| Immunoglobulins  Light chain  Heavy chain  Light & heavy chains | AL-amyloidosis  AH- amyloidosis  AL/AH- amyloidosis | A  A  A |
| Amyloid-A protein | AA-amyloidosis | A/H** |
| Leukocyte chemotactic factor 2 | ALECT2 amyloidosis | A |
| Fibrogen α | AFib amyloidosis | H |
| Lysozyme | ALys amyloidosis | H |
| Apolipoprotein AI | AApoAI amyloidosis | H |
| Apolipoprotein AII | AApoAII amyloidosis | H |
| Apolipoprotein AIV | AApoAIV amyloidosis | A,H |
| Apolipoprotein CII | AApoCII amyloidosis | H |
| Apolipoprotein CIII | AApoCIII amyloidosis | H |
| Gelsolin | AGel amyloidosis | H |
| Transthyretin* | ATTR amyloidosis | H,A |
| Procalcitonin | ACal amyloidosis | A*** |
| Il-1 receptor antagonist protein | AIL1RAP amyloidosis | A**** |

Adapted from (87) and (89)

* In ATTR the heart and peripheral nervous system are primarily involved; kidney involvement is very rare, it can be observed in the hereditary form of ATTR amyloidosis, single cases have been described in wild type ATTR amyloidosis, but kidney involvement was never the sole manifestation.

**Most hereditary forms of AA-amyloidosis concern genetic autoinflammatory diseases, although a familial non-inflammatory AA amyloidosis has been described (90, 91)

*** associated with tumor that produces (pro)calcitonin

**** Iatrogenic, caused by administration of Anakinra (92)

A diagnosis of amyloidosis (in the kidney or a surrogate site) is made by the identification of pale pink, amorphous, silver-negative and acellular deposits in light microscopy, showing congo-red positivity with apple-green birefringence under polarized light. On electron microscopy, amyloid deposits show randomly arranged fibrils of 7-12 nm in diameter. Typing of the involved protein requires the use of immunofluorescence and/or immunohistochemistry studies. In hereditary amyloidosis, DNA typing will reveal amyloidogenic mutations. It is important to realize that a mutation in itself is not sufficient as diagnosis: some mutations do not cause amyloidosis, and some mutations even protect against amyloidosis (e.g some mutations in lysozyme or thransthyretin) (93). Preferably, a diagnosis of hereditary amyloidosis should be supported by two techniques e.g. confirmation of a mutation in the fibrinogen gene and positive staining of amyloid tissue with anti-fibrinogen antibodies. A definite diagnosis of AL-amyloidosis requires the identification of a monoclonal light chain as constituent of the fibrils with detection of the M-protein/ free light chains in serum or urine. Additional studies may be necessary to definitely rule out other types/causes of amyloidosis (Table 2.2). Mass spectrometry is recommended when an expert center fails to identify the subtype the amyloid deposits by IF, immunohistochemistry (using appropriate antibodies)(40, 41, 94), or immune-electronmicroscopy.. Unfortunately, this technique is costly, and not always available.

Table 2.2 Tools to evaluate amyloidosis

| Histology | Immunohistochemistry with appropriate antibodies  Immunofluorescence  Immuno-electronmicroscopy    Antibodies against κ and λ light chains, Ig heavy chains, Amyloid-A protein, Transthyretin, Fibrinogen, LECT-2, Lysozyme, Apolipoprotein A-I |
| --- | --- |
| Genetic | Mutation analysis  Transthyretin, Fibrinogen, Lysozyme, Apolipoprotein A-I, Apolipoprotein A-II, Apolipoprotein CII, Apolipoprotein CIII, Gelsolin. |
| Biochemistry** | Mass spectrometry of amyloid containing deposits. |
|  |  |

** not widely available

**Summary statements**

***Diagnosis, evaluation and management of patients with MIg-associated amyloidosis should be done in (consultation with) expert centers.***

***A diagnosis of MIg-associated amyloidosis requires histological proof of the presence of amyloid, and unequivocal confirmation of the culprit light and/or heavy chain as the constituent of the amyloid fibrils.***

***Early detection and treatment of MIg-associated amyloidosis is needed to prevent organ damage and improve survival.***

***MIg-associated-amyloidosis should be suspected in patients who present with unexplained nephrotic syndrome, or patients with proteinuria and/or kidney failure especially in the presence of extrarenal organ damage involving the heart, liver, gastrointestinal tract, soft tissues or nervous system.***

***We recommend thorough evaluation of patients with renal MIg-associated amyloidosis to detect extrarenal manifestations. Perform routine echocardiography and measure cardiac biomarkers (NT-proBNP or BNP, and troponin). Additional studies should be done in patients with clinically suspected organ involvement (MRI heart, nerve conduction studies, autonomic function tests, abdominal ultrasound).***

***Patients with MIg-associated amyloidosis should receive detailed hematologic evaluation including serum (and urine) protein electrophoresis, and immunofixation, and serum FLC measurement as well as bone marrow aspirate with flowcytometry, bone marrow biopsy, and fluorescent in situ hybridization (FISH) of bone marrow plasma cells.***

***In patients with confirmed MIg-associated amyloidosis the following should be used to guide treatment: age, systolic blood pressure, cardiac function, eGFR, oxygen saturation, autonomic failure, presence of peripheral neuropathy, performance status and liver dysfunction.***

***In patients with MIg-associated amyloidosis, and involvement of heart or severe kidney injury, a rapid decrease in serum light chains is warranted. Treatment should be aimed at achieving complete hematologic response.***

***In patients with MIg-associated amyloidosis, who are eligible for hematologic therapy we recommend treatment according the guidelines of the International Society of Amyloidosis. The currently preferred induction treatment consists of a combination of Daratumumab, Cyclophosphamide, Bortezomib and Dexamethasone or CyBorDEX if Daratumumab is unavailable. Bortezomib should be used cautiously in patients with polyneuropathy or suspected lung fibrosis.***

***We suggest early and close monitoring of hematologic response parameters, at least monthly after start of therapy, and consideration of second line therapy especially in patients not using Daratumumab, patients with high baseline difference in concentration of the involved and non-involved light chain (dFLC), early non-response, or absence of organ response after 6-18 months with a persistent M-protein/abnormal FLC.***

***We suggest treatment with HDM-ASCT in selected patients according to the guidelines of the International Society of Amyloidosis. It is possible to defer ASCT in patients who have achieved complete hematologic response after initial hematologic therapy.***

***Regular monitoring by measurement of FLC,serum SPEP/IFE, and urine IFE is required in the follow-up of patients with hematologic response. Early treatment must be considered in patients with a relapse, with choice of therapy dependent on duration of response, previous therapy, and patients’ condition. Treatment guidelines are expected to be updated regularly.***

**Rationale**

***Diagnosis, evaluation and management of patients with MIg-associated amyloidosis should be done in (consultation with) expert centers***

MIg-amyloidosis is a rare disease, and associated with a very high early mortality rate, especially in patients with cardiac involvement. Diagnosis should be done quickly and must be accurate, and early start of treatment is required. Patients with amyloidosis need management by an expert multidisciplinary team. The patients will benefit from consultation with expert centers where patients are counseled by a team consisting of nephrologists, hematologists, cardiologists, neurologists, pathologists, and laboratory specialists. In view of the many subtypes of amyloidosis, and the high prevalence of MGUS, the first and most important step is firmly establishing a correct diagnosis of MIg-associated amyloidosis.

**A diagnosis of MIg-associated amyloidosis requires histological proof of the presence of amyloid, and unequivocal confirmation of the presence of the culprit light and/or heavy chain as constituent of the amyloid fibrils**

In patients with kidney injury, light microscopical evaluation of the kidney biopsy often suggests the presence of amyloid deposits characterized by congo-red positivity with apple-green birefringence under polarized light. Electron microscopy shows the typical randomly arranged fibrils of 7-12 nm in diameter. Correct identification of the culprit protein is essential for patient management. This requires thorough evaluation of tissue biopsies. There are different methods for the identification of the amyloidogenic protein. Often the presence of a protein in amyloid deposits can be determined by appropriate immunostaining. Immunofluorescence on frozen tissue is the golden standard, using antibodies against κ and λ light chains and IgG, IgA, IgM heavy chains. When necessary antibodies against other amyloidogenic proteins should be used. Sometimes antibodies, which are often raised against the intact, full length folded protein, can be unreliable, since in amyloid tissue epitopes can be absent or hidden. This is particularly known for anti-λ antibodies. False positive and negative staining is common and remains a crucial confounder. Therefore, many centers use a panel of antibodies to allow multiple staining for the light chains. A diagnosis of MIg-associated amyloidosis requires the unequivocal identification of the culprit light and/or heavy chain in the deposits. In AL-amyloidosis the causative light chain is most often of the λ subtype. When appropriate, IgG subclass staining should be performed. Preferably, the diagnosis of AL-amyloidosis is strengthened by a combination of clinical findings and the presence of the culprit light or heavy chain in serum or urine and/or the presence of a pathogenic clone in the bone marrow. It is required to perform immunostaining for AA-amyloid in all patients with suspected MIg-amyloidosis, since immunoglobulins can be entrapped in AA-amyloidosis When in doubt, additional investigations are needed to correctly identify the amyloid subtype.

***Early detection and treatment of MIg--amyloidosis is needed to prevent organ damage and improve survival.***

MIg-amyloidosis is a systemic disease. Untreated, survival is poor, with a high mortality rate within one year after diagnosis, especially in patients with cardiac involvement. Prognosis has markedly improved attributed to early diagnosis and the introduction of immediately started and more effective therapy. Still, many patients with severe disease have poor outcome despite effective chemotherapy. A large and rapid decrease (within 1 week - 3 months) of the involved FLC (and in parallel dFLC) is associated with improved outcome.

***MIg-amyloidosis should be suspected in patients who present with nephrotic syndrome, or patients with proteinuria and/or kidney failure and evidence of extrarenal organ damage involving the heart, liver, gastrointestinal tract, or nervous system.***

Although MIg-amyloidosis should always be considered in the differential diagnosis of a patient who presents with nephrotic syndrome, kidney response to therapy is more likely if AL-amyloidosis is diagnosed in an early stage i.e before the onset of nephrotic syndrome or the development of CKD stage 3b. Since the prevalence of CKD and MGUS in an elderly population is high, a practical approach is needed. In this respect, the ratio 24h proteinuria (in mg/day)/eGFR (in ml/min/1.73m^2^) might be useful to guide the decision to perform a kidney biopsy. Since AL-amyloidosis is a systemic disease, the suspicion of a diagnosis of AL-amyloidosis should be raised in any patient with CKD and evidence of extra-renal organ damage. We suggest that evaluation of a patient for extrarenal damage using easily available biomarkers might be helpful when in doubt (see below).

***We recommend thorough evaluation of patients with renal MIg-amyloidosis to detect extrarenal manifestations. Perform routine echocardiography and measure cardiac biomarkers (NTproBNP, BNP, troponinT). Additional studies should be done in patients with suspected organ involvement (MRI heart, nerve conduction studies, autonomic function tests, abdominal ultrasound).***

AL-amyloidosis is a systemic disease. The most severe complication is involvement of the heart. Therefore, patients with MIg-amyloidosis should routinely be tested using cardiac biomarkers (NTproBNP or BNP and troponinT) and cardiac ultrasound. Cardiac MRI is more sensitive than ultrasound to detect cardiac involvement and should be considered when available in selected patients. Other extrarenal manifestations must be carefully evaluated. If the clinical or laboratory evaluation suggests extrarenal involvement consultation with other specialties is advised (neurology, gastro-enterology etc)

***Patients with MIg-associated amyloidosis should receive detailed hematologic evaluation including serum and urine electrophoresis, and immunofixation, and serum FLC as well as bone marrow aspirate with flowcytometry, bone marrow biopsy, and fluorescent in situ hybridisation (FISH) of bone marrow plasma cells.***

Patients with MIg-amyloidosis have an underlying clonal Plasma-cell or clonal B-cell disorder. Still, many patients (90%) will fulfill the criteria of MGRS. Most patient have measurable MIg in serum or urine, or an increased FLC ratio and dFLC. Approximately 15% of patients have dFLC < 50 mg/l, and 1-5% may have no abnormalities in serum or urine. We advise to perform BM examination in all patients with MIg-associated amyloidosis independent of the results of serum and/or urine M-protein assays, and additional studies as discussed in Chapter I.

.

***In patients with confirmed MIg-associated amyloidosis the following should be used to guide treatment: age, systolic blood pressure, cardiac function, eGFR, oxygen saturation, autonomic failure, peripheral neuropathy, performance status and liver dysfunction.***

Although the introduction of chemotherapy has improved survival, it is important to realize that patients with severe disease or elderly, frail patients have poorer outcomes and may succumb due to advanced end organ failure, despite start of effective chemotherapy. Thus, in such patients treatment may be futile, and the decision should balance benefits and risks, and ensure survival with quality of life. .

***In patients with MIg-associated amyloidosis, and involvement of heart or severe kidney injury, a rapid decrease in serum light chains is warranted. Treatment should be aimed at achieving complete hematologic response***

Patients with MIg-amyloidosis and involvement of the heart are at very high risk for early mortality. Patienst with severe kidney injury (proteinuria > 5g/day or eGFR < 50 ml/min/1.73m^2^) are at very high risk of developing ESRD. There is a clear association between the decrease of serum light chains within 1 week to 3 months after start of therapy and outcome. Organ response is dependent on reaching hematologic response. Achieving at least very good partial hematologic response is associated with improved outcome.

***In patients with MIg-associated amyloidosis who are eligible for hematologic therapy we recommend treatment according the International Society of Amyloidosis (ISA) guidelines. The currently preferred induction treatment consists of a combination of Daratumumab, Cyclophosphamide, Bortezomib and Dexamethasone OR - if Daratumumab is not available - a combination of Cyclophosphamide, Bortezomib and Dexamethasone. Bortezomib should be used cautiously in patients with polyneuropathy or suspected lung fibrosis.***

Historically, AL-amyloidosis was associated with a very poor outcome. The use of Melphalan-prednisone only slightly improved survival rates. The introduction of bortezomib-containing regimens, which resulted in a faster and better hematologic response rate contributed to improved renal response and higher survival rates. The addition of daratumumab resulted in more rapid and higher hematologic complete response rates. Therefore, Daratumumab is a major advantage especially in patients with cardiac amyloidosis where achieving a rapid hematologic response is crucial. The choice of therapy is based on patient characteristics, the eligibility for HDM-ASCT, center preference, and drug reimbursement based on national authority regulations. It is expected that treatment modalities will change over the years. Therefore, we refer to the 2022 ISA guidelines, and the updates. (see discussion in the supportive evidence paragraph). In patients with neuropathy, bortezomib should be used cautiously and other regimens may be used (e.g. lenalidomide-dexamethasone, daratumumab single agent, or a daratumumab-Lenalidomide combination, carfilzomib-dexamethasone, or melphalan-Dexamethasone). Issues related to treatment of (planned) kidney transplant recipients are discussed in chapter X.

***We suggest early and close monitoring of hematologic response parameters, at least monthly after start of therapy, and consideration of second line therapy especially in patients not using Daratumumab, patients with high baseline dFLC, patients with early (one month) hematologic non-response, or absence of organ response after 6-18 months with persistent M-protein or abnormal FLC.***

There is an association between hematologic response and renal response. Thus, renal response is observed only in patients with hematologic complete or very good partial response. Hematologic response criteria are used based on a regular evaluation of the MIg, and the (d)FLC. Organ response is associated with the rapidity of hematologic response. Notably, patients with severe kidney injury at start of therapy may develop kidney failure, even when there is a good hematologic response.

***We suggest treatment with HDM-ASCT in selected patients according the guidelines of the International Society of Amyloidosis. It is possible to defer ASCT in patients who have achieved complete hematologic response after initial chemotherapy***

HDM-ASCT is used in selected patients to improve or maintain hematologic remission. Many patients with amyloidosis will not be suitable candidates for HDM-ASCT. The improved hematologic response rate with current therapy likely will affect the use of HDM-ASCT. It is plausible to defer HDM-ASCT in patients with complete hematologic response after chemotherapy.

***Regular monitoring by measurement of FLC, SPEP/SIFE, and UPEP/UIFE is required in the follow-up of patients with hematologic response. Early treatment must be considered in patients with a relapse, with choice of therapy dependent on duration of response, previous therapy, and patients’ condition. Treatment guidelines are expected to be updated regularly.***

Regular measurement of serum FLC and evaluation of serum and urine by electrophoresis and immunofixation will allow early detection of hematologic relapse. The pathophysiology of amyloid fibril formation poses that a lower concentration of monomers is needed to accelerate the fibril growth in case of relapse in patients with persistent amyloid deposits Thus, it can be expected that clinical manifestations will occur more rapidly in case of a relapse. Early treatment appears to improve outcomes in large retrospective studies but prospective data is lacking. The choice for treatment of relapse depends on many variables such as duration of remission, previous treatment, clinical condition. We refer to current ISA consensus statements.

**Supportive evidence**

***Diagnosis, evaluation and management of patients with MIg-associated amyloidosis should be done in (consultation with) expert centers.***

The incidence of MIg-associated amyloidosis is low. Kyle et al. calculated the incidence rate of AL-amyloidosis from 1990 through 2015 in Olmsted County, Minnesota, US at 12 per million person-years (95% CI, 8-16 per million person-years)(95). Rates were similar across the decades 1990-1999, 2000-2009, and 2010-2015 at 11, 9, and 16 per million person-years, respectively, with no suggestion of an increasing rate in this period. In another study from the US, the incidence of AL amyloidosis between 2007 and 2015 ranged from 9.7 to 14.0 cases per million person-years also with no statistically significant increase.(96). Many reviews quote a incidence of 6-12 per million patient years (86). A recent report included 16 studies. The authors calculated a median incidence rate of 12 per million patient years, but suggested that this likely was an overestimation since in some studies, which reported rates as high as 50 per million patients years, prevalence rates were used.(97).

MIg-associated amyloidosis is associated with a very high and early mortality rate, especially in patients with cardiac involvement. Thus, diagnosis should be done quickly and must be accurate, and early start of treatment is required. Patients with MIg-amyloidosis need management by an expert multidisciplinary team. The patients will benefit from consultation with expert centers where patients are counseled by a team consisting of nephrologists, hematologists, cardiologists, neurologists, pathologists, and laboratory specialists. In view of the many subtypes of amyloidosis, and the prevalence of MGUS, the first and most important step is firmly establishing a correct diagnosis of MIg-associated amyloidosis

***A diagnosis of MIg--associated amyloidosis requires histological proof of the presence of amyloid, and unequivocal confirmation of the presence of the culprit light and/or heavy chain as constituent of the amyloid fibrils.***

Congo-red staining is a sensitive technique to detect amyloid deposits. Some authors suggest that a evaluating congo-red by immunofluorescence using ultraviolet light and special filters (TRITC filter) is more sensitive and allows detection of tiny amounts of amyloid (as can be observed in glandular tissue). In clinical practice, in kidney biopsies of patients with kidney injury, standard congo-red staining and detection of birefringence using polarization filters is the golden standard. Immunostaining, preferentially using fresh frozen tissue, is the first step toward identification of the amyloid subtype. For the detection of the various subtypes of MIg-amyloidosis, antibodies against the heavy and light chains are used. The specificity and especially the sensitivity of immunostaining is debated. A study from 2002 is often quoted to warn against misdiagnosis of MIg-amyloidosis(98). This study reported detection of a genetic variant in 34 out of 350 patients with suspected AL-amyloidosis. Mutations were found in Fibrinogen (n=18), Transthyretin (n=13), ApoAI (2), and lysozyme (n=1). An M-protein in serum or urine was detected in 8/34 patients. A close look at the data showed that in most patients a definite tissue diagnosis of AL-amyloidosis was lacking. In fact, immunostaining for light chains was negative in all patients with hereditary amyloidosis and in 195/316 patients (62%) with non-hereditary amyloidosis. Thus, in this study the sensitivity of immunostaining to diagnose AL-amyloidosis was unusually low at 38%, although specificity was 100%.

It is evident that the presence of a MIg in serum or urine, or an abnormal FLC ratio is insufficient to diagnose monoclonal immunoglobulin associated amyloidosis. Thus, the suboptimal sensitivity of immunostaining presents a problem. However, the percentage of false negative staining is dependent on the technique used and the panel of antibodies. In recent years, sensitivity has increased. Schönland et al. summarized 6 studies and reported that 2-33% of amyloid biopsies remained unclassified.(94) The authors reassessed the accuracy of immunohistochemistry in a prospective, blinded matter, in patients with amyloidosis with assessment of clinical features, histology, and genetics. Importantly, in the study period there were 353 patients with amyloidosis, of these 156 had a negative or inconclusive immunohistochemical analysis and were included in the study. The excluded patients (n-197) had mostly undergone kidney biopsy, evaluated by experienced pathologists (likely diagnosed with AL amyloidosis). Of the 156 patients, 39 were excluded (37 for localized amyloid). The remaining 117 patients were studied. Only 14 patients had undergone a kidney biopsy, most tissue concerned biopsies of the heart (47) or gut (41). Immunohistochemistry was done with commercially available antibodies against serum AA, fibrinogen, lysozyme, transthyretin, κ and λ chains, and at the time non-commercially available antibodies against ApoA1 and λ chains (3 different antibodies). Genomic analysis included Transthyretin, Fibrinogen, ApoA1, ApoAII, and Lysozyme. Sensitivity and specificity of immunohistochemistry are shown in the tables 2.3 and 2.4. Table 2.3 included patients with an established diagnosis based on clinical, laboratory and genetic data, thus allowing independent assessment of the sensitivity and specificity of the IHC technique. It is evident that sensitivity and specificity are very good. Table 2.4 included patients where a formal diagnosis of a defined amyloid subtype was not possible, thus the diagnosis was mainly based on the histological (IHC) findings, and where applicable confirmed by genetic studies.

Table 2.3 accuracy of IHC in patients with amyloidosis subtype confirmed by clinical, laboratory and genetic data (table adapted from (94)

|  | clinical | Typical symptoms | M-protein | Positive DNA | Family history | sensitivity | Specificity |
| --- | --- | --- | --- | --- | --- | --- | --- |
| AL | 39 | 39 | 39 | 0 | 0 | 39/39 | 100% |
| ATTR | 5 | 0 | 0 | 5 | 2 | 4/5 | 100% |
| AApoAI | 2 | 0 | 0 | 2 | 1 | 2/2 | 100% |
| AFib | 4 | 0 | 0 | 4 | 1 | 3/4 | 100% |
| AA | 1 | 0 | 0 | 0 | 0 | 1/1 | 100% |

Table 2.4 results of IHC staining in patients with unknown subtype amyloidosis. Table adapted from (94)

|  | clinical | Typical symptoms | M-protein | Positive DNA | Family history | IHC positive |
| --- | --- | --- | --- | --- | --- | --- |
| AL | 56 | 0 | 56 | 0 | 0 | 52/56 |
| ATTRvariant | 3 | 0 | 2 | 3 | 1 | 3/3 |
| ATTR wild type | 7 | 0 | 0 | 2 | 0 | 6/7 |

False positive findings were not reported. Sensitivity is also good, still, in 4/56 patients with AL amyloidosis immunostaining failed to identify the culprit protein. Ideally, in these cases, the confirmation of amyloid fibril type needs to be undertaken by another method such as LCMS or immune-electronmicroscopy (99). In view of the limited availability of these techniques, it may be acceptable to treat patients as AL-amyloidosis, if there is a correct clinical phenotype, in the presence of a monoclonal Ig in serum or urine, if the other relevant hereditary and acquired amyloid types have been ruled out, or the AL-amyloid subtype confirmed in other tissues. Although there are many other amyloid proteins associated with kidney involvement (see Table 2.1), most can be diagnosed (or ruled out) using immunofluorescence and immunohistochemistry techniques (eg AA-amyloid, ALECT2, Afibrinogen, ATTR) or genetic studies (eg ATTR, Afibrinogen, Alysozyme, Agelsolin). A diagnosis of ATTR may be suspected by the characteristic visualization of the heart on bone scintigraphy.

The specificity of immunostaining is almost 100% (94, 98), certainly when evaluated by expert pathologists. (42) However, some reports suggest that false positive staining does occur (100) (Gonzalez-Suarez 2019). In the latter study, LCMS was used a golden standard technique, and the authors concluded that immunofluorescence had lower sensitivity and specificity. There were 170 renal biopsies with a written report; of these 104 cases were positive for AL amyloid. IF was reported as negative in 16 (sensitivity 85%). On the other hand, there were 66 cases negative for AL amyloidosis (mostly AA and Alect2). Of these 5 were reported positive in IF. Thus, false positive rate was 5/93 (5%, specificity 95%). Of note: all 5 had confirmed AA amyloidosis by LCMS, and in two patients staining for AA was positive (in three not done). A recent report noted that in all patients with suspected MIg-amyloidosis routine immunostaining for AA-amyloid is required, in view of the entrapment of immunoglobulins in AA-amyloid. (5) Thus, it is likely that routine staining for AA would have suggested an alternative diagnosis. Most importantly, in this study the IF results were based on reports of several participating institutions, of unknown quality, unknown antibody characteristics and not validated. Therefore, false positive staining is infrequent, if at all. To address this problem, it remains important that a proper diagnosis of the amyloid subtype requires positive confirmation in the appropriate IHC/IF staining, in parallel with exclusion of other subtypes depending on the clinical characteristics (eg AA amyloid in patients with inflammation, genetic forms of amyloid in patients with a positive family history etc).

Importantly, literature data are likely biased, since the reports are based on the evaluation of biopsies in expert centers. Accuracy of the diagnosis of amyloidosis and assessment of its subtype will vary between centers dependent on the expertise, the used antibodies, performance of genetic analysis etc. This further underlines the awareness of the difficulties in diagnosing the amyloid subtype, the necessity to have confirmation of the diagnosis by independent techniques/procedures, and the importance to consult expert centers.

Although a kidney biopsy is usually performed to diagnose kidney amyloidosis, we suggest that a kidney biopsy can be omitted in patients with severe proteinuria, and documented amyloid present in a biopsy of skin, abdominal fat pad, rectal mucosa, bone marrow, or other organ; where the amyloid is stained positive for a MIg and the presence of the corresponding MIg in serum or urine is confirmed.

***Early detection and treatment of MIg-amyloidosis is needed to prevent organ damage and improve survival.***

MIg-amyloidosis is a systemic disease. Untreated, survival is poor. In patients who participated in a RCT in the period 1982-1992 median survival was only 8 months in “untreated” patients (colchicine was used in all), which improved to 18 months in patients treated with melphalan-prednisolone (101). Overall survival was worst in patients with cardiac involvement (median survival 5 months vs 15 months in patients with kidney involvement and 34 months in patients with peripheral neuropathy). Prognosis has markedly improved in recent years, likely attributed to early diagnosis and the introduction of immediately started and more effective therapy. Muchtar et al. compared outcome among 1551 newly diagnosed AL-amyloidosis patients seen at the Mayo Clinic between 2000 to 2014.(102) As compared with the periods 2000-2004 and 2005-2009, patients diagnosed in 2010-2014 were less likely to have >2 involved organs. The rate of equal or better than very good partial response was highest in the period 2010-2014 compared to 2005-2009 and 2000-2004 (66% vs 58% vs 51%; *P* = .001). In addition overall survival was improved in both the ASCT and non-ASCT population over time and fewer patients died within 6 months of diagnosis in the 2 later periods (24% vs 25% vs 37%; *P* < .001). Similarly, kidney outcome has improved in patients with renal amyloidosis. In a study that included patients with renal AL amyloidosis treated with HDM-ASCT, ESKD rate was lower in the period 2012-2020 than in the period 2003-2011, although still associated with renal stage at diagnosis: The 3 yr cumulative incidence of ESKD was 0% vs 6% for patients in stage I, 5% vs 13% for patients in Stage II, and 24% vs 46% for patients in Stage III.(103) The differences are likely explained by changes in treatment (more induction pre-ASCT in the recent period; more full dose conditioning in the most recent period). Importantly, patients in the most recent period presented with lower renal stage, reflecting earlier diagnosis. In a recent RCT time interval between diagnosis and start of therapy was 48 days. (104). Patients with severe disease (e.g. systolic blood pressure < 100 mm Hg; severe heart failure with NT pro BNP> 8,500 ng/l (1,000 pmol/l), severe autonomic dysfunction, bad performance status; bilirubin >2 mg/dl) have poorer outcome and, despite effective hematologic therapy, may succumb due to advanced end organ failure. Such patients are usually not included in RCT’s.

***MIg-associated amyloidosis should be suspected in patients who present with nephrotic syndrome, or patients with proteinuria and/or kidney failure and evidence of extrarenal organ damage involving the heart, liver, gastrointestinal tract, soft tissues or nervous system.***

Patients with MIg-associated amyloidosis and kidney involvement typically present with nephrotic syndrome. It is important to diagnose AL-amyloidosis in an early stage: treatment may not prevent renal failure in patients who present with severe kidney injury. A risk score, using eGFR <50 ml/min/1.73m^2^ and proteinuria > 5 g/day as risk biomarker, predicted outcome: 5 yr renal survival was approximately 90% if no risk factor was present at the start of therapy, 70% if only one risk factor was present, and 30% if both risk factors were present.(105). As mentioned above, renal outcome has improved in recent years, however the renal stage has remained an important predictor. Therefore, it is important to diagnose AL-amyloidosis in an early stage well before the onset of nephrotic syndrome or the development of CKD stage 3b. AL-amyloidosis thus should be in the differential diagnosis in patients with moderate proteinuria or CKD stage 2-3a. Since the prevalence of CKD and MGUS in an elderly population is high, a practical approach is needed. In this respect, the ratio of 24h proteinuria (in mg/day)/eGFR (in ml/min/1.73m^2^) might be useful.(106) The latter study included consecutive patients with newly diagnosed AL-amyloidosis and kidney involvement, treated and followed in a single center in Greece. The ratio of 24h proteinuria to eGFR proved a sensitive marker of renal risk: a ratio <30 (in mg/ml/min/1.73 m^2^ ) was associated with a 2-year progression to dialysis-dependency of 0% compared to 9% for a ratio of 31-99 and 35% for a ratio ≥100 (P < .001). Furthermore, patients who achieved a reduction of this ratio ≥25% or reached a value ≤100 (if initially >100) at 3 months had a 2-year progression to dialysis of 0% vs 24% for patients who either did not reduce to or still had a ratio >100 (P = .001). Since this ratio is not regularly used in clinical practice we provide some examples: a ratio of 30 translates in a proteinuria of 1,000 mg/day and eGFR 35 ml/min/1.73m^2^ or proteinuria 1,500 mg/day and eGFR of 50 ml/min/1.73m². These data can be used to guide the decision to perform a kidney biopsy.

Since AL-amyloidosis is a systemic disease, the suspicion of a diagnosis of AL-amyloidosis should also be raised in a patient with CKD and evidence of extra-renal organ damage. Cardiac involvement is most feared, and not easily detectable in an early stage. We suggest that assessment of easily available biomarkers might be helpful when in doubt. Typical examples are the cardiac biomarkers NTproBNP and TroponinT (see below table 2.5).

Notably, although AL-amyloidosis is predominantly associated with glomerular injury (causing proteinuria and nephrotic syndrome), AL-amyloidosis may be present in patients with decreased eGFR but no or minimal proteinuria. In these patients amyloid deposits are mainly present in the tubulo-interstitial compartment or in the vasculature. (107)

***We recommend thorough evaluation of patients with renal MIg-associated amyloidosis to detect extrarenal manifestations. Perform routine echocardiography and measure cardiac biomarkers (NTproBNP, BNP,and TroponinT). Additional studies should be done in patients with clinically suspected organ involvement (MRI heart, nerve conduction studies, autonomic function tests, abdominal ultrasound).***

AL-amyloidosis is a systemic disease. The most severe complication is involvement of the heart. Therefore, patients with MIg-amyloidosis should routinely be tested using cardiac biomarkers and cardiac ultrasound. NTproBNP, BNP and troponinT are the best studied and most frequently used biomarkers. NTproBNP is a very sensitive biomarker, and it has been shown that a normal NTproBNP level excluded heart involvement. (108, 109). In contrast, specificity if limited since NTproBNP levels are affected by age, inflammation, and BMI. Particularly relevant is the role of eGFR in NTproBNP clearance: high levels of NTproBNP are observed in patients with CKD, values increasing more than 10-fold in patients on dialysis. (109). Some centers use BNP, although for diagnostic and prognostic purposes NTproBNP performs better. Importantly, in one study BNP was normal in 17% of patients with heart involvement, indicating lower sensitivity. (109). BNP might be more suited in patients with CKD stage V, since in patients with CKD stage V serum levels of BNP are less increased (threefold) than serum levels of NTproBNP. However, it remains unproven if the use of BNP in this population is of real added value. In the literature a conversion formula can be found, which allows to calculate NTproBNP based on BNP. (110) When using NTproBNP as prognostic biomarker, it should be realized that most data derive from studies in patients with preserved eGFR. Although NTproBNP is very sensitive, a cardiac ultrasound is advised in all patients with AL-amyloidosis to detect possible cardiac involvement. Cardiac involvement is typically signalled by increased thickness of the interventricular septum, often accompanied by pericardial effusion, a restrictive filling pattern, and a reduced ejection fraction. An abnormal cardiac ultrasound in combination with increased NTproBNP almost proofs cardiac amyloidosis. However, a normal ultrasound does not exclude cardiac involvement in a patient with abnormally elevated NTproBNP. Since specificity of NTproBNP is limited, other techniques are used to evaluate cardiac amyloidosis. Cardiac MRI is more accurate in identifying cardiac involvement in amyloidosis. Late gadolinium enhancement (LGE) and increased myocardial extracellular volume (ECV) are typical findings in cardiac amyloidosis when using contrast MRI. The increased sensitivity was shown in one study. (111) In this study an abnormal ultrasound was observed in 32/35 patients with histological proven cardiac amyloidosis, whereas LGE was noted in 34/35. In this study, almost all patients with suspected cardiac amyloidosis and abnormal ultrasound had abnormal MRI. In contrast, 47% of patients with normal ultrasound had abnormal LGE, and this correlated with BNP: mean BNP was 267 pg/ml in patients with abnormal LGE and 84 pg/ml in patients with normal LGE. Myocardial ECV might even perform better than LGE both for diagnostic and prognostic purposes.(112). Since the use of gadolinium contrast is debated in patients with low eGFR, some centers prefer non-contrast MRI, using T1 mapping. Indeed, in a meta-analysis T1-mapping was equal to LGE for diagnostic purposes, but less suited for prognostic purposes. (112) In another study, threshold values for T1 mapping were suggested, with values < 1036 ms having very high (98%) negative predictive value and values > 1164 ms similarly high positive predictive value. (113). The authors suggested that gadolinium MRI could be restricted to the patients with intermediate probability (T1 between 1036 and 1164 ms), which include in their study 58% of patients.

In patients with MIg-amyloidosis other non-cardiac, extrarenal manifestations must be carefully evaluated (table 2.5). If the clinical or laboratory evaluation suggests extrarenal involvement consultation with other specialties and additional studies are advised.

Table 2.5 organ involvement in AL amyloidosis

| **Heart** |
| --- |
| Edema, fatigue, hypotension |
| Heart failure with preserved ejection fraction |
| Low voltage ECG |
| Thickened ventricular wall (ultrasound), increased myocardial ECV , late gadolinium enhancement (MRI) Less negative left ventricular global longitudinal strain |
| Increased NTproBNP (or BNP) |

| **Nervous system** |
| --- |
| Peripheral nervous system: sensorimotor polyneuropathy |
| Carpal tunnel syndrome |
| Autonomic: postural hypotension, erectile dysfunction, intestinal motility disorders |

| **Gastrointestinal tract** |
| --- |
| Malabsorption, diarrhea, constipation, weight loss |

| **Liver** |
| --- |
| Increased Alkaline phosphatase |
| Hepatomegaly |
| Increased bilirubin |

| **Other** |
| --- |
| Periorbital purpura |
| Macroglossia |
| Soft tissue |
| Factor X deficiency |

Adapted from (86)

***Patients with MIg-amyloidosis should receive detailed hematologic evaluation including serum and urine electrophoresis, and immunofixation, and serum FLC measurement as well as bone marrow aspirate with flow cytometry, bone marrow biopsy, and fluorescent in situ hybridization (FISH) of the bone marrow plasma cells.***

Patients with MIg-amyloidosis have an underlying clonal Plasma cell or clonal B cell disorder. Still, few patients have an underlying hematologic malignancy, and approximately 90% will fulfill the criteria of MGRS (114). Most patients have a measurable MIg in serum or urine, or an increased dFLC or abnormal FLC ratio. For a detailed discussion of laboratory evaluation see Chapter I. Approximately 15% of patients have dFLC < 50 mg/l, and 1-5% may have no hematologic abnormalities in serum/urine. We advise to perform BM examination in all patients with MIg-amyloidosis independent of the results of serum and/or urine M-protein assays, and additional studies as described in chapter I.

***In patients with confirmed MIg-associated amyloidosis the following should be used to guide treatment: age, systolic blood pressure, cardiac function, eGFR, oxygen saturation, autonomic failure, presence of peripheral neuropathy, performance status and liver dysfunction.***

Although the introduction of chemotherapy has improved survival, it is important to realize that patients with severe disease and extra-renal involvement (as manifested by systolic blood pressure <100 mm Hg; severe heart failure with NT-proBNP > 8,500 ng/l, severe autonomic dysfunction, bad performance status; bilirubin >2 mg/dl) have poorer outcomes. The same holds for elderly, frail patients. Thus, in these patients treatment may be futile, and a treatment decisions should be guided by quality of life and not length of survival. ’’.

***In patients with MIg-associated amyloidosis, and involvement of heart or severe kidney injury, a rapid decrease in serum light chains is warranted. Treatment should be aimed at achieving complete hematologic response***

Patients with MIg-amyloidosis and cardiac involvement are at very high risk of early mortality, whereas patients with severe kidney injury will develop ESRD. A rapid decrease of the involved FLC is needed in patients with severe disease. This was illustrated in an analysis of patients enrolled in the prospective UK AL amyloidosis chemotherapy study (ALchemy). (115). The data showed that patients who achieved ≥ 90% decrease of dFLC within three months after start of therapy had significantly better overall survival (undefined vs 31.8 months, p= 0.02), prolonged time to dialysis (23 vs 6.1 months, p=0.003), and prolonged time to the composite endpoint of 'death or dialysis' (17.3 vs 5.3 months, p<0.001) compared to those who achieved lesser degrees of hematologic response at the same time point. Of note, in this study this good hematologic response was observed in 34% of patients who received Bortezomib based therapy. Unfortunately, outcome was not optimal in patients who reached the threshold of 90% reduction of dFLC beyond three months after start of therapy. Clearly evaluation of the response after 3 months is too late, and not preferred in patients with severe disease. In a subsequent study of the same cohort it was shown that assessment after 1 month might be justified: patients who had developed CR or VGPR at 1 month after start of therapy had good outcome.(116) In this study, all patients had received Bortezomib therapy upfront, good hematologic response was observed at 1 month in one third of the patients (CR 12.1%, VGPR 23.8%). There was no difference in survival between patients who developed CR or VGPR. Patients who do not rapidly respond to treatment, should switch to an alternative regime early (thus preferably based on regular evaluation of the effect of therapy after 1 and 3 months. The advantage of adding Daratumumab to standard therapy is likely explained by the more rapid response rate.(104) Daratumumab thus is particularly beneficial in the treatment of patients with cardiac involvement, where a rapid response is crucial. Rapid measurement of dFLC after start of daratumumab based therapy should also be considered. Liu et al. evaluated 48 patients, all treated with daratumumab/Bortezomib/Dexamethasone (+/- cyclpphosphamide). A decrease > 67% of dFLC after 1 week was associated with hematologic and renal response after 6 months (CR 90% versus 23%, renal response 72% versus 27%).(117) In another analysis, it was shown that dFLC > 87% or VGPR after 1 cycle (4 weeks) performed equally well. (118) Baseline dFLC has also been quoted as important risk biomarker. In the Bortezomib era, baseline dFLC > 180 mg/l was associated with worse outcome. However, in patients treated with daratumumab, baseline dFLC was no longer associated with outcome, underlining that the rapidity of response now is most informative. (118). Most predictor studies excluded patients with low baseline dFLC (< 50 mg/l). Dittrich et al. reported better outcome in patients with low dFLC. (119) This study included 106 patients with dFLC < 50 mg/l. Daratumumab was not used. Treatment consisted of MelDex, BorDex, or HDM-ASCT. Overall outcome was good, with 5 year survival of 80%, approaching 100% in patients treated with HDM-ASCT. Nguyen et al. also reported good survival (median 9.2 years) in patients with AL-amyloidosis, who presented with dFLC < 50 mg/l.(120) Patients with cardiac stage I also carry a good prognosis. This information can be used in risk assessment and treatment decisions.

***In patients with MIg-associated amyloidosis, who are eligible for chemotherapy we recommend treatment according the guidelines of the International society of Amyloidosis. The currently preferred induction treatment consists of a combination of Daratumumab, Cyclophosphamide, Bortezomib and Dexamethasone OR if Daratumumab is not available a combination of Cyclophosphamide, Bortezomib and Dexamethasone or Bortezomib, Melphalan, and Dexamethasone. Bortezomib should be used cautiously in patients with polyneuropathy or suspected lung fibrosis.***

Historically, AL amyloidosis was associated with a very poor outcome. The use of Melphalan-prednisone only slightly improved survival rates. The introduction of bortezomib-containing regimens, which resulted in faster and better hematologic response rates contributed to clearly improved renal response and higher survival rates. The addition of daratumumab resulted in even higher hematologic complete response rates, and superior organ response. After 2 years there was no significant difference in overall patient survival. .(104) However, difference in overall survival was noted after long-term follow-up. Kastritis et al. presented the data at the meeting of the American Society of Hematology, published in abstract form only. (121). Median follow-up was 61 months. Estimated overall 5 year survival amounted 76% with daratumumab and 65% in the control group. Of note, in this latter study (the Andromeda trial) most patients had cardiac involvement, a condition where ultrafast response is most crucial. The choice of therapy is based on patient characteristics, eligibility for HDM-ASCT, center preference, and national guidelines and reimbursement regulation. It is expected that treatment modalities will change over the years. Therefore, we refer to the 2022 ISA guidelines, and the updates.(122, 123) In patients with evident neuropathy (grade 1 painful neuropathy or grade 2 and higher sensory neuropathy), bortezomib is not preferred and other regimens are used (e.g. lenalidomide-dexamethasone, daratumumab single agent, a daratumumab-Lenalidomide combination, carfilzomib-dexamethasone, or melphalan-Dexamethasone). Issues related to treatment of (planned) kidney transplant recipients are discussed in chapter X.

Although induction treatment consisting of a combination of Daratumumab, Cyclophosphamide, Bortezomib and Dexamethasone is advised, there are still many unsolved questions, which explains the debate in many countries regarding use and reimbursement of Daratumumab in all patients with MIg-amyloidosis. The Andromeda trial included patients with severe disease, with most patients suffering from cardiac involvement. The control group received CyBorDEx for 6 months and no maintenance thereafter. The experimental group received Dara+CyBorDex as induction therapy, and maintenance Daratumumab from 6 to 24 months. Many questions remain:

1. Is it necessary to continue daratumumab as maintenance in patients who have develop complete hematological response after 6 months.

2. In patients with no complete response, is maintenance with daratumumab more effective than consolidation with HDM-ASCT (in eligible patients) or even maintenance with Bortezomib or Lenalidomide-Dexamethasone.

3. In low risk patients (no cardiac involvement, low dFLC, eGFR > 50 ml/min/1.73m^2^ , is induction with Daratumumab superior to sequential therapy starting with a combination of Cyclophosphamide, Bortezomib and Dexamethasone, with assessment of FLC response after 4 weeks, and addition of daratumumab in patients with response less than VGPR.

As discussed above, baseline criteria as well as early dFLC response allow to identify low risk patients. The added value of daratumumab in these patients has not been proven. Indeed, in a retrospective study, that evaluated the value of FLC burden, no difference in outcome was observed in patients with baseline dFLC < 180 mg/l, treated with Bortezomib based regimen with or without daratumumab. (124)

Relevant information is given by Yohannan et al. (125). These authors performed a retrospective analysis of patients with AL-amyloidisis, treated in the period 2018-2022, who received either Daratumumab+CyBorDex or CyBorDex as first line therapy. During follow-up many patients who initiated CyBorDex received second line therapy (presumably because of insufficient hematological response). Follow-up was 30 months in the Daratumumab group and 59 months in the control group, illustrating the more recent introduction of Daratumumab. The two cohorts were not fully comparable, there were more patients with cardiac stage I and kidney stage I in the Daratumumab group. Hematological response was quicker in the Daratumumab group (after 2 months VGPR/CR in 61% versus 31%). Cardiac but not renal response was significantly better in the Daratumab group. Estimated 2 year overall survival was also higher, 82% versus 70%. Interestingly, there was no difference in survival when comparing patients who received Daratumumab as first line therapy and patients who started with CyBorDex and received Daratumumab during follow-up.

The role of HDM-ASCT in the Daratumumab era is less well defined. See paragraph below. On the other hand, the Andromeda trial excluded patients with most severe cardiac injury (stage 3B). Outcome in these patients is considered dismal. Data from small, retrospective studies suggest that the addition of daratumumab to standard therapy improves outcome in these patients. (126, 127). Oubari et al. retrospectively compared 27 patients who received Daratumumab based therapy and 71 patients treated mostly with Bortezomib based therapy. Overall, hematological response, cardiac response, and survival were higher in the daratumumab group (after 6 months at least VGPR 58% vs 32%; cardiac response 46% vs 21%; survival 70% vs 51%). In a comparable study 31 patients treated with daratumumab based therapy were compared with 31 controls ( Bortezomib based therapy). Mean follow-up was 28 months. Daratumumab treatment resulted in improved hematologic response (at 3 months VGRP/CR 41% vs 16%), and survival (median survival 10.3 versus 4 months). Still, prognosis is poor in these patients, and mortality is very high and amounts 10% per month in the first 4 months. The study of Yohannan et al. included 48 patients with cardiac stage IIIB. Approximately 45% of these patients had died within 6 months after start of therapy, with no difference between the two treatment groups. Also, estimated 2 year event free and overall survival was dismal (12.5% and 15.6% respectively in the daratumumab group, compared to 9.3% and 40.6% in the control group, a not significant difference). These data underline the need for further studies and development of treatment protocols.

***We suggest early and close monitoring of hematologic response parameters, at least monthly after start of therapy, and consideration of second line therapy especially in patients not using Daratumumab, patients with high baseline concentration of the involved and non-involved light chain (dFLC), patients with early (one month) hematologic non-response, or absence of organ response after 6-18 months with persistent M-protein/abnormal FLC***

There is an association between hematologic response and renal response. Thus, renal response is observed only in patients with hematologic complete or very good partial response. Hematologic response criteria are used based on a regular evaluation of the monoclonal Ig, and the concentration of the free light chains (Table 2.6). Notably, patients with severe kidney injury at start of therapy may develop kidney failure, even when there is a good hematologic response. As such, kidney outcome is associated with grading of kidney involvement (see above). Renal outcome is also associated with renal response, of which criteria are given in table 2.6 (128). It is important to be aware that in the latter study the analysis was restricted to patients who were treated and had achieved at least partial hematologic response. Notably, as discussed above, data suggest that hematological and renal response can be predicted by measurement of dFLC within 1 week – 1 month after start of therapy. Therefore, we suggest to monitor FLC response at least 4 weeks, and preferably at one week after start of therapy.

Table 2.6 response criteria

| **Hematologic response** |  |
| --- | --- |
| Complete response: | Two criteria must be met:  absence of amyloidogenic monoclonal protein defined by negative immunofication electrophoresis of serum and urine  **AND**  FLC ratio within reference range or uninvolved FLC concentration is greater than involved FLC concentration (with or without normal FLC ratio) |
| Very good partial response:* | dFLC < 40 mg/l |
| Partial response | >50% reduction in dFLC |
| **Renal response** |  |
| Complete response** | Proteinuria < 0.2 g/day |
| Very good partial response** | >60% reduction in proteinuria |
| Partial response** | >30% and <60% reduction in proteinuria |
| No response | < 30% reduction in proteinuria |

Adapted from Anand et al. (79) and (128)

*If the initial dFLC is < 50 mg/liter, a dFLC < 10 mg/L is considered low dFLC partial response (which can be considered equal to VGPR).

** renal response requires stable or improved eGFR (decrease eGFR <25%); renal response predicts renal outcome, but only applicable to patients with hematologic response

***We suggest treatment with HDM-ASCT in selected patients according guidelines of the International society of Amyloidosis. It is possible to defer ASCT in patients who have achieved complete hematologic response after initial chemotherapy***

HDM-ASCT is used in selected patients to improve or maintain hematologic remission. In a recent study, only one third of patients were treated with HDM-ASCT, indicating that the majority of patients with AL amyloidosis are not considered suitable candidates for this therapy. Most centers will consider HDM-ASCT in patients who fulfill the following criteria:

- Age > 18 yrs
- Age < 65-70 years
- ECOG performance status ≤ 2
- Cardiac LVEF > 40 % (45%) or interventricular septum thickness < 15 mm
- O_2_ saturation > 95% on room air
- eGFR > 30 ml/min/1.73m^2^
- Systolic blood pressure > 90 mmHg
- Serum bilirubin < 2 mg/dl (< 2*ULN)
- NT-proBNP < 5,000 ng/L

The efficacy of HDM-ASCT is higher if induction therapy with Bortezomib based chemotherapy is used (129). There are no RCT’s comparing the added value of HDM-ASCT in all eligible patients. The improved hematologic response rate with current therapy likely will affect the use of HDM-ASCT. It is plausible to defer HDM-ASCT in patients with complete hematologic response. The role of HDM-ASCT in the Daratumumab era, and the optimal timing is not well defined. There will be differences between countries and centers. As an example we summarize the strategy, described in a guidance paper of the Swiss Amyloidosis Network.(130). Patients with AL-amyloidosis should be assessed for fitness to undergo HDM-ASCT. Treatment (with daratumumab based therapy in plasma cell clone, or Rituximab based therapy in clonal B cell associated IgM amyloidosis) should aim at reaching CR (and at least VGPR after 3-4 months). If no VGPR is reached after 4 months, eligible patients are treated with HDM-ASCT. Although some suggest that assessment of minimal residual disease can be helpful in decision making, the Swiss authors consider that this is subject of clinical trials and not ready for routine use (131).

***Regular monitoring by measurement of FLC, SPEP/SIFE, and IPEP/UIFE is required in patients with hematologic response. Early treatment must be considered in patients with a relapse, with choice of therapy dependent on duration of response, previous therapy, and patient’s condition. Treatment guidelines are expected to be updated regularly.***

Regular measurement of serum FLC and M-protein will allow early detection of hematologic relapse. The pathophysiology of amyloid fibril formation poses that a lower concentration of monomers is needed to accelerate the fibril growth in case of relapse in patients with persistent amyloid deposits(85) Thus, it can be expected that clinical manifestations will occur more rapidly in case of a relapse. Early treatment appears to improve outcomes in large retrospective studies but prospective data is lacking. In the Swiss guidance paper treatment for relapse is suggested in patients with dFLC > 20 mg/l, and increase > 20% of baseline, and > 50% over the lowest value reached during therapy. Earlier start should be considered in patients with cardiac involvement. The choice for treatment of relapse depends on many variables such as duration of remission, previous treatment, and the clinical condition of the patient. We refer to current ISA consensus statements.

**Chapter 3: Monoclonal Immunoglobulin Deposition Disease**

**Introduction**

Monoclonal Immunoglobulin deposition disease (MIDD) is defined by the presence of non-organized deposits of monoclonal immunoglobulin (MIg) chains, usually light chains (LCDD), less commonly, heavy chains (HCDD) or both (LHCDD), along the basement membranes of various organs. Although kidney injury is the most common clinical presentation, extrarenal manifestations can occur.

**Summary statements**

***Diagnosis, evaluation and management of patients with MIDD should be done in (consultation with) expert centers.***

***A histologic diagnosis of MIDD must be established with high degree of certainty, and requires confirmation of the deposition of a monoclonal light and/or heavy chain along the basement membranes. A diagnosis of MIDD should be reconsidered in the absence of positive staining of the TBM. We suggest to perform EM studies, if available, to support the diagnosis of MIDD.***

***In patients with MIDD a search for extrarenal manifestations should be done. We recommend routine echocardiography, measurement of cardiac biomarkers, and liver enzymes. Additional studies (nerve conduction studies, autonomic function tests, abdominal ultrasound) should be done based on history and physical examination.***

***In patients with MIDD, we recommend detailed hematologic evaluation including serum electrophoresis and immunofixation, and serum FLC as well as bone marrow aspirate with flowcytometry and bone marrow biopsy.***

***All patients with MIDD should receive maximal conservative therapy as advised for CKD. In patients with MIDD, who are eligible for chemotherapy we recommend initial clone-directed treatment, aiming at achieving a hematologic complete or very good partial response. The role of HDM-ASCT as consolidation therapy is debated. In suitable patients without deep hematologic response or in patients who do not tolerate standard hematologic therapy, second-line therapy should be considered.***

**Rationale**

***Diagnosis, evaluation and management of patients with MIDD should be done in (consultation with) expert centers.***

Rationale: MIDD are very rare diseases. It is often difficult to establish a definite diagnosis and select the appropriate treatment. Patients with MIDD will benefit from consultation with expert centers where patients are counseled by a team consisting of nephrologists, hematologists, pathologists, laboratory specialists, and others.

***A histological diagnosis of MIDD must be established with high degree of certainty, and requires unequivocal confirmation of the presence of a monoclonal light and/or heavy chain in the kidney basement membranes on immunofluorescence. A diagnosis of MIDD should be reconsidered in the absence of positive staining of the TBM. We suggest to perform EM studies, if available, to support the diagnosis of MIDD.***

Treatment of MIDD is associated with severe side effects. Accurate diagnosis is needed to ensure that treatment is done appropriately. A kidney biopsy is required. In light microscopy, tubular basement membranes are often thickened, showing a ribbon-like appearance. Nodular glomerulosclerosis is the most characteristic glomerular pattern, with or without MPGN features. Vascular PAS-positive deposits can also be observed. All these features are not diagnostic. In MIDD, immunofluorescence (IF) typically shows a linear staining along basement membranes of the involved monoclonal light and/or heavy chain. When appropriate, IgG sub-class staining should be performed. The linear staining along tubular basement membranes (TBM) is a crucial diagnostic feature. In most patients, linear staining is also present along the glomerular basement membrane (GBM), and in many there is positive staining of the mesangium, and around arterial and arteriolar myocytes. In the absence of TBM staining, an alternative diagnosis should be considered. It is not unusual that the term MIDD was used in patients who should have been diagnosed with PGNMID or MIg-associated membranous nephropathy.

Some cases of MIDD may demonstrate a negative staining because of the probable loss of the targeted epitope on the immunoglobulin, or because the antisera to the involved immunoglobulin chain is not included in routine IF (e.g. IgD). In those cases, the diagnosis of MIDD may be suspected on the basis of light microscopy and EM findings. Thus, ultrastructural (EM) evaluation of a kidney biopsy should be performed whenever possible in the evaluation of a kidney biopsy with a suspected diagnosis of MIDD to demonstrate the characteristic powdery punctate deposits along the TBM,GBM and the mesangium. A diagnosis of MIDD can be made without EM, when the kidney biopsy is evaluated by an expert nephropathologist who confirms the typical IF and LM findings. In rare cases, when the diagnosis is unclear, laser microdissection followed by mass spectrometry can be helpful in identifying the involved immunoglobulin chain. Notably, incidental patients have been described in whom a diagnosis of “LCDD by IF only” was made. In some this might represent an early stage of the disease.

***In patients with MIDD a search for detect extrarenal manifestations should be done. We recommend echocardiography, measurement of cardiac biomarkers, and liver enzymes. Additional studies (nerve conduction studies, autonomic function tests, abdominal ultrasound) should be done based on history and physical examination.***

Patients with MIDD typically present with kidney injury. Recent studies suggest an unexpectedly high prevalence of extra-renal involvement in patients with MIDD, especially of the heart and the liver. Because these are recent findings, evaluation is not evidence-based or standardized. We suggest that initial workup of patients with MIDD should include echocardiography, measurement of NT-proBNP and troponinT levels as well as liver enzyme measurement. Additional studies, based on history and physical examination, may include neurological evaluation.

***In patients with MIDD we recommend detailed hematologic evaluation including serum electrophoresis and immunofixation, serum FLC, as well as bone marrow aspirate with flow-cytometry and bone marrow biopsy.***

Patients with MIDD always have an underlying (often small) B-cell or plasma-cell clone. This corresponds to a true underlying hematologic malignancy in only a minority of cases. Most patients will fulfill the criteria of MGRS. Many patients have measurable monoclonal Ig in serum or urine, and (almost) all patients have abnormal FLC ratio and/or increased dFLC. In patients without such abnormalities, the diagnosis of MIDD should be reconsidered. We advise to perform BM examination in all patients with a diagnosis of MIDD independently of serum abnormalities (See chapter I).

***All patients with MIDD should receive maximal conservative therapy as advised for CKD. In patients with MIDD, who are eligible for chemotherapy we recommend initial clone-directed treatment, aiming at achieving a hematologic complete or very good partial response. The role of HDM-ASCT as consolidation therapy is debated. In suitable patients without deep hematologic response or in patients who do not tolerate standard hematologic therapy, second-line therapy should be considered.***

Although not specifically evaluated in this population, all patients with MIDD should receive conservative treatment according CKD guidelines. We recommend clone-targeted therapy in patients with MIDD and preserved eGFR (> 20-30 ml/min/1.73m²). Most patients have plasma-cell clones, and will receive treatment regimens that include bortezomib. In patients with MIDD, treatment with HDM-ASCT can be considered as consolidation therapy, although its role in this setting is debated in view of the high hematologic response rates with current hematologic therapy. HDM-ASCT can also be considered in fit patients without deep hematologic response despite first line bortezomib or immunomodulatory drugs based therapy. In non-responsive patients, patients who do not tolerate Bortezomib or immunomodulatory drugs, or in patients considered unsuitable for HDM-ASCT treatment with anti-CD38 therapy, if available, might be considered. Future studies should evaluate the role of anti-CD38 therapy in patients who are eligible for HDM-ASCT. In patients with low eGFR at diagnosis (<20-30ml/min/1.73m²), renal outcome is dismal, and the advantage of therapy is less evident. Still, in these patients therapy is advised in the presence of symptomatic extrarenal involvement. Hematologic treatment should also be considered in patients awaiting kidney transplantation (see chapter X). We caution against overzealous treatment in the elderly. In these patients survival time must be factored against the quality of life.

**Supportive evidence**

***Diagnosis, evaluation and management of patients with MIDD should be done in (consultation with) expert centers***.

MIDD are very rare diseases. In China a diagnosis of HCDD was made in 0.05% of kidney biopsies, and of LCDD in 0.1% of kidney biopsies (132, 133). In US studies, a diagnosis of MIDD was made in 0.47-0.70% of kidney biopsies (134). In these studies LCDD was most frequent, and accounted for 70-80% of all MIDD. In these and other series of patients with MIDD, the prevalence of LCDD ranged from 63-91%, of HCDD 6-30%, and of LHCDD 3-10%.(132-138). In these large US centers, 2-3 patients with a diagnosis of MIDD are seen per year. A similar incidence was reported from a large, expert center in the UK, with 55 patients with LCDD seen over a period of 13 years (139). Of note, referral bias cannot be excluded. In Europe, most nephrology units are much smaller in size, thus a diagnosis of MIDD will be made less often. For example, a French collaborative study which included patients with MIDD seen in 55 centers over a period of 34 years, collected 255 patients with MIDD, which amounts less than 1 patient per center per 5 years(135). MIDD is not only a rare disease, it is also difficult to establish a definite diagnosis and select the most appropriate treatment. Patients with MIDD will likely benefit from consultation with expert centers where patients are counseled by a team consisting of nephrologists, hematologists, pathologists, laboratory specialists and others.

***A histological diagnosis of MIDD must be established with high degree of certainty, and requires unequivocal confirmation of the deposition of a monoclonal light and/or heavy chain along the kidney basement membranes in immunofluorescence. A diagnosis of MIDD should be reconsidered in the absence of positive staining of the TBM. We suggest to perform EM studies, if available, to support the diagnosis of MIDD.***

MIDD is defined by the presence of non-organized deposits of monoclonal immunoglobulin chains, usually light chains (LCDD), and less commonly, heavy chains (HCDD) or both (LHCDD), along the basement membranes. In most patients with MIDD, the deposited monoclonal immunoglobulin chain is composed of a single light chain, reflecting the higher prevalence of LCDD over HCDD or LHCDD. . In MIDD, the hallmark is linear staining of a monoclonal light and/or heavy chain along the TBM, and the majority of patients have tubular atrophy and interstitial fibrosis (IF/TA). Glomerular basement membrane staining is not always observed, and occurs in approximately 80% of patients. The characteristic nodular glomerulosclerosis is observed in approximately two thirds of these patients. Nodular glomerulosclerosis is more frequent in patients with HCDD than LCDD: the prevalence ranges from 53-83% in LCDD and 60-100% in HCDD (132-135, 140, 141). It is likely that these variations parallel the differences in clinical characteristics at diagnosis (see below). Rarely, reports have included patients with a suggested diagnosis of MIDD, in whom TBM staining was absent (141). Such cases most likely represent PGNMID. We suggest that in the absence of positive TBM staining the diagnosis of MIDD should be reconsidered if not rejected. As discussed in Chapter 1, rare cases of MIDD caused by a monoclonal IgD or IgE will be missed in routine immunofluorescence. Also, negative staining may be explained by the loss of epitopes. In such cases the diagnosis of MIDD may be suggested by EM studies showing the typical punctate powdery deposits. Additional staining (eg with antibodies against IgD) and/or mass spectrometric analysis of laser microdissected kidney tissue may reveal the causative monoclonal immunoglobulin (see chapter 1). In rare cases, a diagnosis of “LCDD by IF only” has been made. (142). The kidney biopsies in these patients showed monotypic light chain restricted staining of the basement membranes in the absence of any abnormality in EM. In most cases, patients were diagnosed with light chain cast nephropathy, and the linear staining may reflect non-pathogenic trapping of the elevated light chain. However, Nasr et al. reported three patients in whom “LCDD by IF only” represented an early stage of the disease. In these patients, all having received a kidney transplant, a first biopsy (often protocol biopsies) showed linear light chain restricted staining of the basement membranes. Repeated biopsies (performed after 13-41 months for progressive kidney injury) showed typical features of LCDD both in IF and EM.

The clinical presentation of patients with MIDD is quite variable. Recent studies have reported details of clinical characteristics and outcome in cohorts of patients with MIDD from the US, UK, France, and China (133-139, 143). Relevant data are given in table 3.1. Most studies did not differentiate between patients with and without an underlying hematologic malignancy, likely introducing bias. Most patients presented with renal insufficiency, hematuria and moderate proteinuria. Less than one third of patients presented with nephrotic syndrome. As expected from the pathology review, patients with HCDD more often presented with nephrotic syndrome than patients with LCDD. In a recent study nephrotic syndrome was noted in 61% of patients with HCDD as compared to 29% of patients with LCDD (135). Of note, in the study of Cohen (an older study from France) these percentages amounted 42% vs 9%, likely reflecting earlier diagnosis in more recent years. It is important to realize that patients with MIDD, and more specifically LCDD, may present with low eGFR in the absence of proteinuria. These patients typically have deposits restricted to the TBM with light microscopy showing marked interstitial fibrosis and tubular atrophy.(144)

***In patients with MIDD a search for extrarenal manifestations should be done. We recommend echocardiography, measurement of cardiac biomarkers, and liver enzymes. Additional studies (nerve conduction studies, autonomic function tests, abdominal ultrasound) should be done based on history and physical examination***

MIDD was considered a renal limited disease. However, recent studies have pointed to the presence of extrarenal manifestations, although the reported prevalence is quite variable. The heart, liver, and nervous system are most frequently involved. Cardiac involvement was reported in 3 - 17% of patients, liver involvement in 3-17%, and neuropathy in 0-9% (135, 137, 143). There is no evidence to recommend any routine assessment, however since MIDD are rare diseases, and treatment decisions should take into account extrarenal disease, we suggest that all patients should at least be evaluated for the presence of cardiac (cardiac ultrasound, NTproBNP, TroponinT) and liver (liver enzymes) involvement. History and physical evaluation will guide additional specialist consultation (e.g neurological evaluation). Other organs involved include the salivary gland, skin, gastro-intestinal tract, and lung.(135). In the latter study extrarenal involvement was more often observed in patients with LCDD than in patients with HCDD (48% vs 26%).

***In patients with MIDD we recommend detailed hematologic evaluation including serum electrophoresis, and immunofixation, and serum FLC as well as bone marrow aspirate with flow-cytometry and bone marrow biopsy***

In most studies, only a minority of patients with MIDD have an underlying hematologic malignancy (4-36%), mostly symptomatic myeloma (Table 3.1). The study of Pianko et al. is the exception, since in this study 77% of patients had a hematologic malignancy (137). This discrepancy is unexplained. Overall, 64-96% of patients of patients with MIDD fulfill the definition of MGRS.

A M-spike is observed in the serum in 32-64% of patients. These data are likely biased by the inclusion of patients with a hematologic malignancy. it is evident that the absence of a serum M spike does not exclude MIDD. In contrast, most studies suggest that an abnormal FLC ratio is seen in almost every patient, including the patients with HCDD (135, 136, 138). There is a clear к predominance (approximately 3-4:1). These findings are seemingly in contrast with the Chinese studies in which an abnormal FLC ratio was observed in only 60% (HCDD) and 85% (LCDD) of patients (132) (133). Of importance, in one Chinese study TBM staining was observed in only 76% of patients with HCDD.(132) The cause for the discrepancies is not entirely clear and implications for outcome, diagnostics and therapy need to be further investigated. As discussed above, in the absence of TBM staining the diagnosis of MIDD must be questioned.

Since serum abnormalities (M-spike, FLC) are the rule, urine analysis is not routinely done. However, in patients with definite MIDD and negative serum evaluation, urine UPEP/UIFE should be performed. Full hematologic evaluation includes bone marrow biopsy and bone marrow aspirate with flow-cytometry to detect the responsible clone, which is often of small size.

***All patients with MIDD should receive maximal conservative therapy as advise for CKD. In patients with MIDD, who are eligible for hematologic therapy we recommend initial clone-directed treatment, aiming at achieving a hematologic complete or very good partial response. The role of HDM-ASCT as consolidation therapy is debated. In suitable patients without deep hematologic response or in patients who do not tolerate standard hematologic therapy, second line therapy should be considered***

Although not specifically evaluated in this population, we advise that all patients with MIDD should receive conservative treatment according to CKD guidelines. The relevance of such treatment, targeting blood pressure, cholesterol, obesity, and life style measures was less evident in the 20^th^ century, when prognosis of patients with MIDD was unfavorable. The introduction of more potent hematologic therapy has improved prognosis, necessitating proper treatment of CKD to attenuate progressive kidney injury (by preventing hyperfiltration injury) and prevent cardiovascular morbidity and mortality.

In older series median kidney survival was 2 years and median overall survival only 4 years (145). In the latter study 65% of patients had multiple myeloma, which certainly had a negative effect on survival. Most importantly, in the older study treatment consisted of melphalan, cyclophosphamide, and steroids, treatment regimens which are associated with low hematologic response rates. Joly et al. evaluated patients with MIDD diagnosed in the period 1981 - 2015 and showed that year of diagnosis < 2004 was associated with a significantly worse outcome (135). This further underlines that the introduction of novel drugs (proteasome inhibitors, immunomodulatory drugs) and HDM-ASCT has improved outcome.

Overall survival is associated with age and hematologic response. Although hematologic response is often followed by kidney response, approximately one third of patients with hematologic response will not develop kidney response. In multivariable analysis, hematologic response and eGFR at presentation were independent predictors of kidney survival. Patients with an initial eGFR < 20 ml/min/1.73m² are less likely to remain free of dialysis, and patients who need kidney replacement therapy at presentation are unlikely to come off dialysis. Since hematologic response is predicting kidney response, treatment should be aimed at reaching CR of VGPR (outcome in patients with VGPR equals outcome in patients with CR). Literature data support the use of Bortezomib-based regimens (see below). Although in most studies a substantial number of patients received HDM-ASCT, it is not proven that this affected renal survival. Therefore, it seems plausible to use Bortezomib-based therapy as initial therapy, and add HDM-ASCT in patients who do not develop CR/VGPR. The use of HDM-ASCT as consolidation therapy in patients with CR/VGPR after initial chemotherapy can be considered, based on center and patient preference. The introduction of antiCD38 drugs (such as daratumumab) has further extended the therapeutic armamentarium. Although its position in the treatment of patients with MIDD has not been established, treatment with daratumumab may be considered in patients with insufficient response to first line therapy, depending on country specific guidelines and reimbursement. Of note, although the use of antiCD38 therapy as initial therapy in MIDD might be suggested based on its efficacy in patients with AL-amyloidosis, there is no evidence in support. Importantly, in most patients with MIDD there is no need to reduce the free light chains as quick as possible. Patients with cardiac involvement may be the exception, although there is no proof. In patients with eGFR < 20 ml/min/1.73m^2^ and no extrarenal manifestations, especially in the elderly, it might be plausible to abstain from treatment. Indeed, very few patients who were on kidney replacement therapy at diagnosis could stop dialysis, and therefore in many studies patients who presented with ESRD did not receive hematologic therapy(143). Of note, although hematologic therapy will not improve kidney function in patients with ESRD, treatment in such patients still can be considered when preparing for kidney transplantation (see Chapter X)

A narrative overview of recent studies that describe treatment and outcome in predominantly Caucasian patients with MIDD is given below. The clinical characteristics of the patients are given in table 3.1.

Sayed *et al.* studied 53 patients with LCDD.(139) At baseline many patients had severe kidney failure, including 57% of patients with CKD stage 4 or 5. Follow-up was 6.2 years. Kidney survival was dependent on eGFR at the time of diagnosis. Median kidney survival (no need for Kidney Replacement Therapy) was 9 years in patients who presented in CKD stage 2 or 3, and 2.7 years in patients who presented in stage 4 or 5 (not already on dialysis). The authors were able to evaluate hematologic and kidney response after first line therapy in 32 patients. Hematologic response was associated with renal response: eGFR improved by 6.1 ml/min/yr in 21 patients who achieved hematologic CR/VGPR and decreased by 6.5 ml/min/yr in 11 patients with hematologic PR/NR. Only 3 out of 21 patients who developed CR/VGPR progressed to ESRD, as compared to 7 out of 11 patients with PR/NR. Ten patients needed kidney replacement therapy at diagnosis. Although not specifically stated, the data suggest that no patient could stop dialysis during follow-up. In this study initial therapy was variable and included Thalidomide, Bortezomib, or an alkylating agent. Hematologic response was numerically lower with thalidomide (5/11) than with Bortezomib (8/9). Overall 16 patients in this cohort were treated with HDM-ASCT, as first-line therapy in only 4. The role of HDM-ASCT cannot be evaluated. Although not specified, it is likely that most or all patients described by Sayed et al. were included in later study from the UK (143). In this study, the long-term outcome of 77 patients diagnosed with LCDD between 1999 and 2018 was assessed. The median eGFR at diagnosis was 23 ml/min/1.73m^2^ and eight patients (10.4%) were dialysis-dependent at diagnosis. None of the latter received hematologic therapy. The kidney response to therapy was assessed after completion of 1^st^ line treatment, which was bortezomib-based in 37.7% of patients, thalidomide-based in 24.6%, or alkylator-based in 21.7%. Rituximab was administered to 4.3% of the cases, steroids as sole treatment were given to 4.3% of the patients and Lenalidomide was used as treatment in 2.9% of the cases. One third of patients also received ASCT. Kidney function improved (defined by a decrease of serum creatinine of at least 25%) or remained stable in 66.6% of the patients. Interestingly, the median time to dialysis was 62 months. Patients with eGFR <30 ml/min/1.73m^2^ at baseline had a significantly shorter time to dialysis (19 months). Moreover, in patients with a poor hematologic response (no CR or VGPR) AND eGFR <30 ml/min/1.73m^2^ median time to dialysis was only 8 months. Thus, hematologic response and eGFR at baseline were solid predictors of renal response.

Kourelis *et al.* reported 88 patients.(138) Follow-up data beyond 12 months were available for 65 patients. During the study period various treatment regimens were used. This study confirmed that kidney response was associated with hematologic response: kidney response was noted in 21/37 (57%) patients with hematologic CR/VGPR and in 9/27 (33%) patients with hematologic PR/NR. Hematologic response was observed more often in patients treated with ASCT (77%) or Bortezomib based therapies (56%) compared to other therapies (6%). Kidney survival was better in hematologic responders (risk ratio 0.1) and worse in patients with baseline eGFR <20 ml/min/1.73m^2^ (risk ratio 6.4). Sixteen patients were on dialysis at diagnosis, 4 of these were not treated. Overall only 2 patients could stop dialysis, both after having developed hematologic CR with therapy.

Cohen *et al.* reported 49 patients.(136). All received Bortezomib based therapy as initial (77.5%) or second line therapy. Good hematologic response (CR/VGPR) was observed in 31 (70%), with CR in 5 and VGPR in 26 patients. Again, kidney response was associated with hematologic response. Overall, there were 26 patients with a kidney response: there were 22 responders in 31 patients with dFLC < 40 mg/l after therapy (71%) versus 4 in 18 patients with dFLC > 40 mg/L (22%). In this study there was no significant difference in response rate between patients who received HDM/ASCT or not (hematologic response 100% versus 85%, kidney response 61% vs 48%, time to progression 8.4 versus 8.1 years). Obviously, bias cannot be excluded, since the decision for ASCT was not specified. Patients treated with HDM/ASCT were younger, although there was no difference in baseline serum creatinine or underlying hematologic disease.

Joly et al. described 255 patients.(135) This study likely included patients reported by Cohen et al. Many patients were diagnosed and treated before 2004. Hematologic response, the use of Bortezomib, and baseline eGFR were associated with prognosis. Response rate in patients treated with Bortezomib was 60% versus 25% in patients treated with regimens without Bortezomib.

Kastritis et al. retrospectively analyzed 25 patients with MGRS who received daratumumab therapy.(146) This study included 22 patients with MIDD (LCDD in 20). Daratumumab was given either as first line therapy (n=12) or as second/third line therapy. Daratumumab was given with dexamethasone (n=14), with Bortezomib/dexamethasone/cyclophosphamide (n=10) or with Lenalidomide/dexamethasone (n=1). Median age was 61 yr (38-83), and 76% were males. Median eGFR was 26 ml/min/1.73m^2^ (6-102), and median proteinuria 2.1 g/day (0.74-16). Three patients were on dialysis. An abnormal M-protein and/or abnormal FLC ratio were present in 22 patients. In 2 patients without abnormal FLC ratio, a pathological clone was detected in the bone marrow. Median follow-up was 14 months. Overall hematologic CR and VGPR was achieved in 10 patients (out of 23 evaluable patients; 44%), and hematologic PR in another 7 (30%). Overall hematologic response was not significantly different when comparing daratumumab as first-line therapy versus second line therapy (80% vs 69%), the combination of daratumumab with Bortezomib (Dara-VCD) was more effective (overall response 90% vs 62%; deep hematologic response (CR+VGPR) 82% vs 21%). Kidney response was evaluated in patients not on dialysis (patients on dialysis did not respond). In the majority of patients proteinuria decreased while eGFR remained stable after 6 and 12 months. A clear improvement of eGFR by at least 25% was seen in only three patients. In a parallel study the authors described the effect of daratumumab as consolidation therapy in patients without CR after first line therapy. This cohort of 25 patients included 19 patients with AL-amyloidosis and 6 patients with MIDD (147). Hematologic response was evaluated 4 weeks after a short course of daratumumab (4 doses in one month). In the MIDD patients there was improved response, although overall not very impressive (which might be explained by the short follow-up): at start of therapy 3 patients had PR and 3 patients had VGPR, after therapy 1 patient was in CR, 3 in VGPR, and 2 maintained PR. Although interesting, the role of daratumumab as first line therapy is not defined and a comparison with standard therapy (Bortezomib based) is needed. Daratumumab may be of value in patients with limited response to first line therapy. In such patients HDM-ASCT might also be considered, and a comparison is needed. Daratumumab might be especially useful in patients who do not tolerate standard therapy and/or who are not eligible for HDM-ASCT.

The role of upfront ASCT was evaluated by Garderet et al. (148). In this retrospective study the authors summarized data of 51 patients from 24 centers that participated in the registry of the European Society for Blood and Bone Marrow transplantation. Mean age was 55 yr, 63% were male. Most patients had severe kidney injury, 17 patients (of 39 with data available) were treated with renal replacement therapy, and median eGFR in patients not receiving RRT was 52 ml/min/1.73m^2^. Median proteinuria was 0.6 g/day (IQR 0.2-2.0). The majority of patients had multiple myeloma (62%), limiting the applicability of this study to patients with MGRS. Unfortunately there were missing data in 20-40% of patients. ASCT was done median 7 months after diagnosis, and in all but two patients preceded by induction therapy (Bortezomib in 89%). At the time of ASCT hematologic response was CR 12%, VGPR 29% and PR 31%. This improved to CR 44%, VGPR 23% and PR 33% at day 100 after ASCT. Treatment related and overall mortality after 6 months was only 2%. Overall 6 yr survival was good at 88%. Renal response was limited: only 1 out of 17 patients who were on RRT at the time of ASCT were able to stop RRT, approximately 14 months after ASCT. In patients not on RRT, eGFR stabilized. This study thus illustrates the low likelihood of renal response in patients with kidney failure despite favorable hematologic response.

Table 3.1 Overview of studies in MIDD: clinical characteristics of patients

| Author  Year | Sayed  2015(139) | Kourelis 2016(138) | Cohen 2015&(136) | Joly  2019&(135) | Li  2016(133) |
| --- | --- | --- | --- | --- | --- |
| Country | UK | USA | France | France | China |
| Period | 2002-2015 | 1992-2014 | NA | 1981-2015 | 2004-2015 |
| Patients (N) | 53 | 88 | 49 | 255 | 48 |
| Diagnoses   - LCDD - HCDD - LHCDD | 100% | 84%  8%  8% | 72%  24%  4% | 83%  9%  8% | 100% |
| Age (yr) | 56 (29-78) | 56 (22-83) | 64 (55-71) | 64 (53-73) | 54 |
| Gender (M/F) | 37/16 | 58/30 | 27/22 | 133/122 | 29/19 |
| Screat (umol/l) |  | 265 (80-1326) | 190 (134-238) | 269 (169-474) | 239 (80-707) |
| eGFR (ml/min/1.73m^2^) | 27 (0-79) | 22 (2.5-83) | 21 (11-32) | 24 (12-43) | NA |
| RRT (N) | 11 | 16 | 9 | 60 | 5 |
| Salb (g/l) | NA | 35 (17-46) | 35 (31-41) | 33 (29-40) | 36 (21-53) |
| UPCR (g/day) | 4.1  (0.1 – 15.5) | 2.4  (0-17.6) | 1.5  (1-4.3) | 1.8  (0.8-4.0) | 2.8  (1.3-5.1) |
| Hematuria (%) | 90% | NA | 73% | 58% | 75% |
| Nephrotic Syndrome (%) | 22% | 42%!! | 16% | 22% | 46%!! |
| Monoclonal Ig |  |  |  |  |  |
| (SPEP/IFE ) | 43% (64% +Urine) | 64% | 55% | ? | 27% |
| Abnormal FLC | 100% | 99% | 100% | ? | 85% |
| K | 81% | 83% | 74% | 81% | 94%@@@ |
| λ | 19% | 17% | 26% | 19% | 6% |
| MGRS  MM  Other | 87%  11%  2% | 78%  21%  1% | 78%  20%  2% | 64%  34%  2% | 75%  25% |
| remarks | Extrarenal 9% |  |  |  | Low C3 in 33% |

MGRS includes SMM (ie plasmacells> 10); MM = multiple myeloma (symptomatic)

NA = not available; & IQR ††nephrotic range proteinuria; RRT = renal replacement therapy

& patients of Cohen are included in the study of Joly; Since Cohen provides detailed information on Bortezomib treated patients, this study is included. In contrast, the study of Nasr et al. 2012 is not included since all patients were included in the paper by Kourelis et al (2016)(134, 138)

@@@IF evaluation

Table 3.1 Continued

| Author  Year | Zhang 2020(132) | Pianko et al.  2023(137) | Ravichandran et al.  2022 (143) |
| --- | --- | --- | --- |
| Country | China | USA | UK |
| Period | 2008-2018 | 1999-2016 | 1999-2018 |
| Patients (N) | 25 | 34 | 77 |
| Diagnoses   - LCDD - HCDD - LHCDD | 100% | 91%  6%  3% | 100% |
| Age | 50 (SD10) | 49.5 (44-59)* | 59 (26-81)** |
| Gender (M/F) | 14/11 | 20/14 | 52/25 |
| Screat (umol/l) | 141 ( 80 – 574) | NA | 242.5 (71-1209) ** |
| eGFR (ml/min/1.73m^2^) | 50 (SD28) | 23.2 (10.9-42.1)* | 23** |
| RRT (N) | 0 | 7 | 8 |
| Salb (g/l) | 30 (SD6) | 3.95 (3.5-4.2)* | NA |
| UPCR (g/day) | 4 (SD2.5) | 2.7 (0.53-5.8)* | 2.8 (0.1-15.5)** |
| Hematuria (%) | 89% | 15 | NA |
| Nephrotic Syndrome (%) | 40% | 44 | NA |
| Monoclonal Ig |  |  |  |
| (SPEP/IFE ) | 40% | 32% | NA |
| Abnormal FLC | 60% | 100% | NA |
| K | NA | 91% | 79% |
| λ | NA | 6% | 21% |
| MGRS  Symptomatic MM  Other | 96%  4% | 21%#  74%  3% | 80.6%#  15.5%  3.9% |
| Extrarenal disease  Heart  Liver  Neuropathy |  | 3% | 14%  13%  3% |
| remarks | Low C3 in 68% Low C4 in 24%  >10% PC in 95% | Study included 54 patients; data provided of 34 treated patients | LCDD was a selection criterium  % abnormal FLC not given, likely almost all patients based on dFLC of 451 (2.4-25690) |

*Median (IQR), ** Median (range) ^#^inferred from data given, Smouldering multiple myeloma is included in the MGRS category. NA not available.

We excluded the study of Nara et al (2022), since in this study the majority of patients with HCDD/LHCDD had no TBM staining, and EM pictures were not provided.

The study of Ravichandran and Sayed are from the same institution, and likely have considerable overlap.

**Chapter 4: Cryoglobulinemia**

**Introduction**

Cryoglobulinemia is defined as the presence of detectable circulating immunoglobulins that precipitate with cold temperature and dissolve with rewarming. Cryoglobulins are classified according to the composition and clonal restriction of the immunoglobulins that are present in the cryoglobulin fraction.

**Summary statements**

***Evaluate serum for the presence of cryoglobulins in patients who present with kidney injury accompanied by one or more of the following signs/symptoms: purpura, ischemic ulcers, neuropathy, arthralgias, and asthenia /fatigue.***

***Evaluate serum for the presence of cryoglobulins in patients who present with kidney injury and histological findings suggestive of cryoglobulin-associated disease, i.e. a membranoproliferative pattern of glomerular injury with intra-capillary hyaline pseudo-thrombi and/or subendothelial deposits (with substructure).***

***We recommend a kidney biopsy in patients with cryoglobulins in serum and evidence of kidney injury (AKI, CKD stage 3-5, hematuria and/or proteinuria)***

***Evaluate serum for the presence of cryoglobulins in patients with acute or progressive kidney injury and isolated decreased C4 level.***

***A repeated evaluation of serum for the presence of cryoglobulins should be done in patients with suspected cryoglobulinemia if the first evaluation is negative. In such case, special attention should be given to the pre-analytical phase, to guarantee that serum from withdrawal to separation is continuously maintained at 37°C. Also, a period of 7 days is needed to evaluate precipitation. The absence of detectable cryoglobulins in serum does not exclude existence of cryoglobulinemic glomerulonephritis.***

***In a patient with cryoglobulinemia type I, perform thorough hematologic evaluation to identify an underlying hematologic malignancy.***

***In a patient with cryoglobulinemia type II, perform thorough evaluation to identify an underlying disease. Specific attention should be given to: infections (HCV, HBV, HIV), auto-immune diseases (ANA, ENA, Schirmer test, total IgG), and hematologic malignancies.***

***In a patient with cryoglobulin-associated kidney disease and a diagnosis of MGRS, treatment should be guided by disease severity, patient characteristics, and comorbidity. Patients with kidney injury (eGFR < 60 ml/min/1.73m^2^, or proteinuria > 3 g/day, or AKI) should be considered patients with severe disease.***

***We suggest that in selected patients with cryoglobulin-associated kidney disease and MGRS treatment should consist of clone-targeted therapy. Rituximab is first-line therapy in most patients with no detectable clone, although the risk of a Rituximab-induced flare must be considered. The goal of treatment is reduction of the monoclonal Ig load and remission of disease.***

***In a patient with cryoglobulin-associated kidney disease as manifestation of MGRS, consider plasmapheresis in patients with acute kidney injury (and evidence of thrombi or high cryoglobulin levels), patients with evidence of the hyperviscosity syndrome, and patients with high cryoglobulin levels and severe disease such as ischemic ulcers, ischemic cerebral events, or other severe organ damage.***

**Rationale**

***Evaluate serum for the presence of cryoglobulins in serum in patients who present with kidney injury accompanied by one or more of the following signs/symptoms: purpura, ischemic ulcers, neuropathy, arthralgias, and asthenia /fatigue.***

***Evaluate serum for the presence of cryoglobulins in patients who present with kidney injury and histological findings suggestive of cryoglobulin-associated disease i.e a membrano-proliferative pattern of glomerular injury with intra-capillary hyaline pseudo-thrombi and/or subendothelial deposits with substructure.***

***We recommend a kidney biopsy in patients with cryoglobulins in serum and evidence of kidney injury (AKI, CKD stage 3-5, hematuria and/or proteinuria).***

***Evaluate serum for the presence of cryoglobulins in patients with acute or progressive kidney injury and isolated decreased C4 level.***

Cryoglobulin-associated disease can present with variable manifestations: most characteristic are constitutional symptoms (asthenia, fatigue), skin abnormalities (palpable purpura, cutaneous ulcers), neurologic manifestations (sensory or motor neuropathy, cerebral infarction), and vasomotor injury (Raynaud, digital ischemia). Cryoglobulinemia can cause (organ) limited disease or present as systemic disease. Systemic disease caused by cryoglobulins is often defined as Cryoglobulinemic Vasculitis (CryoVasc). Approximately half of patients with cryoglobulin-associated kidney injury have no extra-renal manifestations, whereas kidney involvement occurs in about one quarter of patients with CryoVascIsolated C4 depletion is a characteristic manifestation of cryoglobulinemia. Histologically, the most frequent pattern of injury is membranoproliferative glomerulonephritis with numerous infiltrating monocytes/macrophages. When present, intracapillary PAS-positive pseudo-thrombi are a helpful clue to the diagnosis. Small arteries and arterioles may show vasculitis. By IF, type I cryoglobulinemia shows staining for the involved MIg. In type II and III, staining for IgG and IgM with both kappa and lambda is usually present, reflecting the composition of the deposits. By EM, there are immune-type electron-dense deposits in a subendothelial and mesangial location. Concomitant subepithelial deposits can occur, and in rare cases pure subepithelial deposits are observed. Although the deposits may look amorphous, at higher magnifications substructures are often seen, with curvilinear, microtubular, or annular appearance. In rare patients crystalline intracapillary deposits can be found (crystalglobulin induced nephropathy).

***A repeated evaluation of serum for the presence of cryoglobulins should be done in patients with suspected cryoglobulinemia if the first evaluation is negative. In such case, special attention should be given to the pre-analytical phase, to guarantee that serum from withdrawal to separation is continuously maintained at 37°C. Also, a period of 7 days is needed to evaluate precipitation. The absence of detectable cryoglobulins in serum does not exclude existence of cryoglobulinemic glomerulonephritis***

The detection of cryoglobulins requires a very tightly controlled work-up. Failure to detect cryoglobulins is often explained by insufficient precautions, mostly occurring in the pre-analytical phase, i.e. the period that starts with blood withdrawal and includes the transfer to the lab. It is essential that blood always remains at a temperature of 37 °C, during withdrawal, transport, coagulation and serum separation. Maintaining a lower temperature may lead to precipitation of the cryoglobulins before serum separation, leading to false–negative results. Although cryoglobulins often precipitate within hours to days, slower precipitation can occur and the precipitate may be visible only after 7 days. The detection rate of cryoglobulins in serum might increase using novel techniques. Importantly, with current assay techniques cryoglobulins are not always detected in patients with histologically defined, characteristic lesions of cryoglobulinemic glomerulonephritis.

***In a patient with cryoglobulinemia type I perform thorough hematologic evaluation to identify an underlying hematologic malignancy.***

Although type I cryoglobulins are typically associated with B cell lymphoproliferative diseases, such as Waldenström’s macroglobulinemia, non-Hodgkin B cell lymphoma or multiple myeloma, they can be found in patients without overt hematologic disorders. In such cases, a diagnosis of MGRS should be made.

***In a patient with cryoglobulinemia type II, perform thorough evaluation to identify an underlying disease. Specific attention should be given to: infections (HCV,HBV, HIV), auto-immune diseases (ANA, ENA, Schirmer test, total IgG), and hematologic malignancies***.

Cryoglobulinemia type II can be the consequence of a hematologic malignancy, usually an IgM-secreting hematologic disorder. More often, other underlying causes can be identified. In many developing countries hepatitis C virus infection is the underlying cause in 60-95 % of patients with Type II cryoglobulins. This was also the case in Western countries before the availability of direct acting antiviral drugs. In more recent years, non HCV associated cryoglobulinemia type II has become predominant in Europe., Other causes include hepatitis B infection, Sjögren's syndrome, or other infections or systemic autoimmune diseases. In 30-50% of patients with non-HCV associated cryoglobulinemic vasculitis no underlying cause is identified, these patients often are diagnosed as “essential (“idiopathic”) mixed cryoglobulinemia” and should be considered MGRS. Type III cryoglobulins do not contain a MIg, therefore these are not further discussed here.

***In a patient with cryoglobulin-associated kidney disease and a diagnosis of MGRS, treatment should be guided by disease severity, patient characteristics, and comorbidity. Patients with kidney injury (eGFR < 60 ml/min/1.73m^2^, proteinuria > 3 g/day, or AKI) should be considered patients with severe disease.***

Cryoglobulin-associated disease (CryoVasculitis) are rare diseases. It is pivotal to exclude underlying causes, since treatment primarily should target these underlying diseases. Importantly, while antiviral therapy eradicates the virus in HCV-associated cryoglobulinemia, antiviral therapy alone usually is not sufficient to manage all manifestations such as glomerulonephritis, mononeuritis, or ischemic ulcers. If no underlying cause and no hematologic malignancy is identified, patients with type I or type II cryoglobulinemia will fulfill the criteria for MGRS. The presence of cryoglobulins in serum should not be considered sufficient to claim causality. Patients should have signs/symptoms that are typical for Cryovasculitis or have histological evidence of cryoglobulin-induced injury. Patients with CryoVasc are often aged, and are at risk of infections related to treatment. Thus, treatment must be selected based on comorbidity, age, the severity of the disease, the underlying cause and previous treatment. In patients with kidney injury due to cryoglobulins, the main goal of therapy is to improve kidney function, reduce proteinuria, and prevent progression to ESKD.

***We suggest that in selected patients with cryoglobulin-associated kidney disease and MGRS treatment should consist of clone-targeted therapy. The goal of treatment is reduction of the MIg load and remission of disease.***

Treatment modalities in patients with cryoglobulinemia have been variable. A bone marrow clone can be found, more often in patients with cryoglobulinemia type I than in patients with cryoglobulinemia type II. In a patient with CryoVasc and MGRS, and an underlying clone the treatment should be clone targeted. In patients with no detectable clone (most often type II) initial treatment with Rituximab is suggested, with some caveats (see supportive evidence)

***In a patient with Cryoglobulin-associated kidney disease as manifestation of MGRS consider plasmapheresis in patients with acute kidney injury (and evidence of thrombi or high cryoglobulin levels), patients with evidence of the hyperviscosity syndrome, and patients with high cryoglobulin levels and severe disease such as ischemic ulcers, ischemic cerebral events, or other severe organ damage.***

In patients with high cryoglobulin levels and signs/symptoms compatible with perfusion defects, hyperviscosity, or severe AKI rapid removal of the cryoglobulins requires plasmapheresis (with precautions) combined with therapy directed at reducing the production of the involved immunoglobulins.

**Supportive evidence**

Cryoglobulins are defined as circulating immunoglobulins that precipitate with cold temperature and dissolve with rewarming. Cryoglobulins are classified according to Brouet and based on the composition of the immunoglobulins that are present in the cryoglobulin fraction.(149) Type I cryoglobulins consist of a single MIg, more commonly IgM or IgG with light chain restriction (usually kappa chains). Type II cryoglobulins consists of a combination of a MIg (mostly IgMk) that binds to polyclonal immunoglobulins (mostly IgG). Type III cryoglobulins consist of only polyclonal immunoglobulins, typically polyclonal IgM that binds to polyclonal IgG. The term mixed cryoglobulin is often used and usually encompasses both type II and type III cryoglobulins as they involve two immunoglobulins. Since type III cryoglobulins are not included within the spectrum of MGRS, these are not specifically discussed in this chapter.

Cryoglobulinemia refers to the presence of cryoglobulins in the serum. The mere presence of cryoglobulins is insufficient to diagnose cryoglobulin-associated disease. In a study of 227 patients with type I cryoglobulinemia, the vast majority of patients (191) had no signs or symptoms (150). A diagnosis of cryoglobulin-associated disease, mostly defined as cryoglobulinemic vasculitis (CryoVasc), thus requires the presence of signs and symptoms related to the precipitation of cryoglobulins in the arterioles or the development of immune-complex mediated vasculitis affecting the small to medium-sized blood vessels.

Cryoglobulinemia type I is less common than Cryoglobulinemia type II or III. A UK laboratory study received sera of 887 patients for cryoglobulin evaluation.(151) Of these, 193 sera contained cryoglobulins, with 27 (14.4%) type I, 42 (22.3%) type II, and 119 (63.3%) type III. In a US study that included 486 patients, 109 (22%) were classified as type I, 258 (53%) as type II, and 119 (25%) as type III (152). Studies from France estimated that between 10.3% and 19.8% of patients with cryoglobulinemia had type I, with the remaining classified as mixed cryoglobulins without further specification.(153, 154) Whereas the data on cryoglobulinemia type I are quite consistent, data on the mixed cryoglobulins are quite variable, and dependent on inclusion or exclusion of patients with underlying disease (such as HCV), patients with and without kidney injury, and laboratory performance. Most important is probably the difference in physicians’ attitude toward ordering a serum cryoglobulin test, in some centers cryoglobulin testing may be routine in patients with systemic or auto-immune diseases (eg SLE) or infections (HCV), whereas others will search for cryoglobulins only in patients with signs or symptoms of CryoVasc.

***Evaluate serum for the presence of cryoglobulins in patients who present with kidney injury accompanied by one or more of the following signs/symptoms: purpura, ischemic ulcers, neuropathy, arthralgias, and asthenia /fatigue.***

***We recommend a kidney biopsy in patients with cryoglobulins in serum and evidence of kidney injury (AKI, CKD stage 3-5, hematuria and/or proteinuria)***

***Evaluate serum for the presence of cryoglobulins in patients who present with kidney injury and histological findings suggestive of cryoglobulin associated disease (membrano-proliferative pattern of glomerular injury with intra-capillary hyaline pseudo-thrombi and/or subendothelial deposits with substructure)***

***Evaluate serum for the presence of cryoglobulins in patients with kidney injury and isolated decreased C4 level.***

The presence of cryoglobulins in serum is not sufficient to diagnose cryoglobulin-associated disease, since patients with both type I and type II cryoglobulinemia may be asymptomatic (150, 155, 156). The presentation of patients with symptomatic cryoglobulinemia is quite variable: most characteristic are constitutional symptoms (asthenia, fatigue), skin abnormalities (infiltrative purpura, cutaneous ulcers), neurologic manifestations (sensory or motor neuropathy, cerebral infarction), vasomotor injury (Raynaud, digital ischemia), and glomerulonephritis.(157, 158). The umbrella term Croglobulinemic Vasculitis is often used (CryoVasc).

The clinical characteristics of patients with cryoglobulinemia type I are illustrated in Table 4.1. This table summarizes retrospective studies, mostly including patients with cryoglobulin associated vasculitis (systemic disease) and excluding patients with HCV(150, 152-154, 159). One exception is the study of Sidana et al. which included a small percentage of patients with HCV (8/102).(152) Many patients were diagnosed in the 20^th^ century. In these studies, mean age ranged from 59 to 65 years, and there was no gender dominance. Kidney involvement was present in 14 to 35 % of patients. Patients with kidney injury often presented with acute kidney injury and/or nephrotic range proteinuria. Approximately 50% of patients with cryoglobulin-associated kidney injury may have no extra-renal manifestations (150). There is no clear predominant Ig subtype in cryoglobulinemia type I, with IgM being present in 41-69% of patients. Kidney involvement is more frequent in patients with IgG subtype cryoglobulins (e.g 54% in IgG versus 19% in IgM associated cryoglobulinemia)(159). In almost all patients with (symptomatic) cryoglobulinemia type I hematologic abnormalities are detected (table 4.1). In one study no hematologic abnormalities (no M-protein, normal FLC ratio, no Bence Jones proteinuria, no Bone Marrow abnormalities) were found in 8/102 patients. (152). In 4 patients the underlying disease was HCV. In 4 other patients no underlying disease was found, and the diagnosis was based on the identification of a monoclonal type I cryoglobulin in the kidney biopsy. These patients likely reflect “seronegative” cryoglobulinemic glomerulonephritis (see below) and should be considered MGRS.

The clinical characteristics of patients with cryoglobulinemia type II are illustrated in Table 4.2 (158, 160). Few studies were considered suitable. Many (older) studies included patients with mixed cryoglobulinemia type II and III, thus including patients with and without a monoclonal component. Moreover, most older studies cannot be used since these studies likely included patients with at that time unrecognized HCV infection. Indeed, one study noted that retrospective analysis of blood samples (available in 34 or 102 patients) revealed positivity for HCV in 85%! (161). The largest cohort of cryoglobulin-associated glomerulonephritis in Europe published in 2007 confirmed the strong relationship between HCV and mixed cryoglobulinemia in the 20^th^ century. (162). This Italian cohort included 146 patients, and in the vast majority HCV infection was detected by either positive anti-HCV antibodies, the presence of HCV RNA or both. In only 16 patients (11%) there was no evidence of HCV infection. More recent studies also did not always differentiate between “infectious” and non-infectious mixed cryoglobulinemia. Bryce et al. reported 66 patients, of these 26 were HCV positive at baseline and 40 were HCV positive during follow-up, and 7 were also HBV positive at diagnosis (163). We have not further evaluated studies that included patients with infectious mixed cryoglobulinemia.

Table 4.2 provides details of two studies (158, 160). Of note, the details of patients with renal involvement in the French cohort were reported in a follow-up study, and details are given in Tables 4.3 and 4.4 (164).

The study of Galli et al. included 175 HCV-negative patients with mixed cryoglobulinemia. Of these 96 (55%) were diagnosed with cryoglobulinemia type II. The majority of patients with type II cryoglobulinemia were diagnosed with Sjögren’s syndrome (n=24), SLE (n=6), other auto-immune diseases (n=8), HBV-infection (n=10) or solid tumors (n=2). A lymphoproliferative disorder was diagnosed in 9, and no underlying disease (ie. Essential mixed cryoglobulinemia) was found in 37 patients. There was a female predominance (75%), and kidney involvement was present in only 26 (27%) patients. Notably, in the whole group of patients with essential mixed cryoglobulinemia (n= 69, 37 type II and 32 type III) only 10 patients (14.5%) had kidney involvement, with no separation between patients with type II or type III cryoglobulinemia.

Terrier et al (2012) also excluded patients with HCV, HBV, and HIV infection, and included patients with cryoglobulinemia and either symptoms or biopsy proven evidence of vasculitis.(158) The study included 242 patients with mixed cryoglobulinemia, of which 203 were diagnosed with cryoglobulinemia type II. For comparison, characteristics of patients with cryoglobulinemia type III are included in table 4.2. Renal involvement was more frequent in patients with cryoglobulinemia type II (38% versus 15%). In this study, there was also a female predominance (69%).

Details from this French cohort of 80 patients with renal involvement were published separately (164)(see tables 4.3 and 4.4). In 75 patients the cryoglobulinemia was of type II. The underlying disease was Sjögren’s syndrome in 18 (23%), a hematologic malignancy in 23 (29%) patients (B-cell non Hodgkin lymphoma in 17, Waldenström macroglobulinemia in 5, multiple myeloma in 1), whereas in 49% the diagnosis was essential mixed cryoglobulinemia. In this cohort, 14% of patients had isolated kidney involvement. Since in patients with essential mixed type II cryoglobulinemia a monoclonal component is present in the serum (albeit often only present in the cryoglobulin fraction), these patients fall within the spectrum of MGRS.

In patients with cryoglobulinemia and kidney involvement, a kidney biopsy most often shows a membranoproliferative pattern of glomerular injury with numerous infiltrating monocytes/macrophages.(164) When present, intracapillary PAS-positive pseudo-thrombi are a helpful clue to the diagnosis. Small arteries and arterioles may show vasculitis. By IF, Type I cryoglobulinemia shows restricted staining for the involved MIg. In type II and III, staining for IgG and IgM with both kappa and lambda is usually present, reflecting the composition of the cryoglobulins. By EM, there are immune-type electron-dense deposits in a subendothelial and mesangial location. Concomitant subepithelial deposits occur, in rare cases pure subepithelial deposits were observed. Although the deposits may be amorphous, at higher magnifications substructures are seen in many cases, with curvilinear, microtubular, or annular appearance. (47)

In many patients with cryoglobulinemia complement C3 and/or C4 levels are reduced. Typically, C4 levels are more often reduced than C3 levels. In patients with isolated low C4 levels the presence of cryoglobulinemia should be assessed. Still, C4 levels can be normal in patients with CryoVasc.

It is important to note that in patients with kidney injury and a kidney biopsy showing typical features of cryoglobulinemic glomerulonephritis serum cryoglobulin testing may be negative (even with appropriate testing). A recent study focused on cryoglobulinemia related glomerulonephritis (cryoGN) and compared patients with “seropositive” or “seronegative” cryoglobulinemia (165). This study excluded patients with infection-related cryoglobulinemia. There were 81 patients with biopsy-proven, non-hepatitis CryoGN, 59 (73%) patients with serologic evidence of cryoglobulins (seropositive CryoGN), and 22 (27%) patients without serologic evidence of cryoglobulins (seronegative CryoGN). Clinical characteristics are given in Table 4.5. A total of 33 patients (56%) in the seropositive cohort had type II cryoglobulinemia, 17 (29%) had type I cryoglobulinemia, and 5 (8%) had type III cryoglobulinemia. The type of cryoglobulin was unspecified in the remaining 4 cases (7%). Most patients presented with AKI (frequently with hematuria, and features of a nephritic syndrome). The predominant kidney pathological pattern was MPGN in 78%. Glomerular deposits by immunofluorescence were IgM dominant (84%) in the seropositive group (versus 50% in the seronegative patients). Light chain restriction on IF was observed in 35% in the seropositive group versus 67% in the seronegative cases. Typically, systemic disease was seen less frequently in the seronegative group. Although a similar percentage of patients had a serum M-protein, an abnormal FLC was observed more often in the seropositive group (64%) than in the seronegative group (14%). It is not resolved why serum cryoglobulins are not detected in these patients with “seronegative cryoglobulinemic glomerulonephritis”. It is plausible that the cryoglobulins are preferentially deposited in the glomerulus, with negligible quantities present in serum. Alternatively, current methodology for the assessment of cryoglobulins may be insensitive, as has been suggested by a recent study, which is summarized in the paragraph below. (166)

Rare patients have been described , presenting with AKI and extra-renal manifestations, in whom the kidney biopsy showed intravascular crystals composed of a monoclonal immunoglobulin. The term crystalglobulin nephropathy has been coined. It is important to realize that the composition of the crystals often cannot be determined by routine IF on frozen tissue. In one study, IF on frozen tissue was positive in only 47%, whereas IF on paraffin embedded, pronase digested tissue was positive in 92% of cases. (167)

It is evident that a diagnosis of cryoglobulin-associated kidney disease requires expertise. Many patients will present without the typical features (e.g. not all patients have systemic disease, typical skin lesions, or even measurable cryoglobulins) and consultation with expert centers is advised.

***A repeated evaluation of serum for the presence of cryoglobulins should be done in patients with suspected cryoglobulinemia if the first evaluation is negative. In such case, special attention should be given to the pre-analytical phase, to guarantee that serum from withdrawal to separation is continuously maintained at 37°C. Also, a period of 7 days is needed to evaluate precipitation.***

The detection of cryoglobulins requires a very tightly controlled work-up. Failure to detect cryoglobulins is often explained by insufficient precautions, mostly occurring in the pre-analytical phase, i.e. the period that starts with blood withdrawal(157, 168). It is essential that blood always remains at a temperature of 37 °C, during withdrawal, transport, coagulation, and serum separation. Even a short period of a lower temperature may lead to precipitation of the cryoglobulins before serum separation, leading to false-negative results. The coagulation time is longer at 37°C, and thus a minimum of 1 hour is required. After serum separation, the serum is stored in cold conditions, i.e. 4 °C. Usually, two fractions are stored. Cryoglobulins will precipitate in the cold, with type I cryoglobulins precipitating quicker than type II cryoglobulins. Therefore, although a cryoprecipitate may be observed within 24 hr, it is advised that at least 3 days of incubation are needed, and if negative, to prolong the incubation to 7 days. In one study a cryoprecipitate was observed not until day 7 in 7 out of 66 patients with type II cryoglobulinemia(151). Some authors advise repeating the test 2 or 3 times before declaring negativity. If a precipitate is formed, the sample should be placed at 37°C to demonstrate reversibility. In the last step, the immunoglobulin composition of the precipitate must be evaluated by immunofixation-electrophoresis. This will allow the classification of the cryoglobulinemia as type I, II, or III. A recent study suggested that the detection of cryoglobulins may benefit from using an alternative technique, using hypotonic distilled water to dilute the separated serum 1:1. (166). In this study, 68 patients were evaluated who presented with signs of cryoglobulinemia, but no or only trace amounts of cryoglobulins in serum. In these patients cryoglobulins were detected using the new technique. In most patients (75%) cryoglobulins were polyclonal (type III). Further studies in patients with “seronegative” cryoglobulinemic glomerulonephritis are eagerly awaited.

***In a patient with type I cryoglobulinemia, perform thorough hematologic evaluation to identify an underlying hematologic malignancy.***

Type I cryoglobulins are typically associated with B-cell lymphoproliferative diseases, such as Waldenström macroglobulinemia, and multiple myeloma. In the studies summarized in Table 4.1 an underlying hematologic malignancy (excluding smouldering multiple myeloma) was observed in 42-69% of patients. In type I cryoglobulinemia, the immunoglobulin subtype can be either IgG or IgM. It is suggested that IgG containing cryoglobulinemia type I is more often associated with MGRS, whereas IgM containing cryoglobulinemia type I is more often associated with an underlying hematologic malignancy, specifically Waldenstrom Macroglobulinemia.

In a recent retrospective study from France including 168 patients with cryoglobulinemia type I, 31% of patients were considered as MGRS (better defined as Monoclonal Gammopathy of Clinical Significance since signs and symptoms included other organs than kidney alone), whereas 69% presented with a hematologic malignancy(159). In this study, patients with an IgM cryoglobulin type I were more likely to have a hematologic malignancy. Of 93 patients, the underlying diagnosis was Waldenström Macroglobulinemia in 48%, Multiple Myeloma in 4%, CLL in 2%, and lymphoma in 23%. In 22% MGRS was diagnosed. In 75 patients with IgG cryoglobulin type I the corresponding figures were: Waldenström Macroglobulinemia 0%, Multiple myeloma 30%, lymphoma 16%, and CLL 12%. MGRS was diagnosed in 42%.

***In a patient with cryoglobulinemia type II perform thorough evaluation to identify an underlying disease. Specific attention should be given to: infections (HCV,HBV, HIV), auto-immune diseases (ANA, ENA, Schirmer test, total IgG), and hematologic malignancies.***

Cryoglobulinemia type II is less often the consequence of a hematologic malignancy, usually an IgM-secreting hematologic disorder. A hematologic disorder (mostly lymphoma) was diagnosed in 9/96 patients with type II cryoglobulinemia reported by Galli et al. and in 47/203 patients reported by Terrier et al (158, 160). Most often, other underlying causes can be identified. Before the introduction of direct acting antiviral drugs hepatitis C virus infection was the underlying condition in 60-95 % of patients with type II cryoglobulinemia. Nowadays, in European countries non-HCV associated cryoglobulinemia type II is predominant. Other causes include hepatitis B infection, Sjögren's syndrome, or other infections or systemic autoimmune diseases. In 30-50% of patients with non-HCV associated cryoglobulinemic vasculitis no underlying cause is identified, these patients are given a diagnosis of “essential (“idiopathic”) mixed cryoglobulinemia” and should be considered MGRS.

***In a patient with cryoglobulin-associated kidney disease and a diagnosis of MGRS, treatment should be guided by disease severity, patient characteristics, and comorbidity. Patients with kidney injury (eGFR < 60 ml/min/1.73m^2^, proteinuria > 3 g/day, or AKI) should be considered patients with severe disease.***

***We suggest that in selected patients with cryoglobulin-associated kidney disease treatment should consist of clone-targeted therapy. The goal of treatment is reduction of the monoclonal Ig load and remission of disease. In patients without a detectable clone, rituximab may be considered as first option***

***In a patient with cryoglobulin-associated kidney disease perform plasmapheresis in patients with acute kidney injury (and evidence of thrombi or high cryoglobulin levels), patients with evidence of the hyperviscosity syndrome, and patients with high cryoglobulin levels and severe disease such as ischemic ulcers, ischemic cerebral events, or other severe organ damage.***

Cryoglobulin-associated diseases (CryoVasc) are rare diseases. It is pivotal to identify potential underlying conditions since treatment should be targeted to these diseases (e.g. clone-directed therapy in patients with hematologic malignancy). Although antiviral therapy is indicated in patients with HCV-associated cryoglobulinemia, it is important to note that antiviral therapy is not always sufficient, and often unable to treat the manifestations such as glomerulonephritis, neuritis, and ulcers. Treatment of HCV-associated cryoglobulinemia is not further discussed and we refer to recent literature. (169)

If no underlying cause is identified, both Cryoglobulinemia type I and type II can be considered MGRS, since there is a monoclonal protein present in the serum (or in the kidney biopsy).

There are no randomized controlled trials to guide treatment in patients with MGRS-cryoglobulinemia . Moreover, most of the reported studies have used variable treatment regimens, likely resulting in confounding by indication. Also the reports do not allow to make a clear distinction between patients with and without a hematologic malignancy as well as between patients with and without renal involvement. Thus, response and survival rates mostly concern the overall cohort of patients with CryoVasc. Subanalyses are often not reported. In most studies outcome is limited to overall survival.

A more detailed narrative is given below. Whenever possible, we extracted data on treatment and outcome for patients without hematologic malignancy (thus diagnosed with “MGRS”).

Terrier et al. reported 64 patients with CryoVasc.(154) In 28 the diagnosis was MGRS, and 23 patients received therapy. Initial first line treatment mainly consisted of corticosteroids (n=20), in some combined with alkylating agents (n=5), Rituximab (n=2), or azathioprine (n=1). Plasma exchange was added in 3 patients. Initial response was persistent remission in 8, remission followed by relapse in 9, and non-response in 6. Relapsing and non-responding patients received second (and sometimes third) line of therapy. At final follow-up, all but 5 patients were in remission without therapy. In 5 patients maintenance therapy was used, mainly consisting of plasmapheresis with or without rituximab. Overall survival in the whole cohort at 10 years was 87%, with mortality determined by progression of hematologic malignancy. Thus, 10 year survival in the MGRS subgroup was estimated at 100%. Details of kidney response are not given. Of note, in this study 23 patients received Rituximab as first, second, or third line therapy. The authors report the development of worsening vasculitis in three patients within 48 hr after administration of Rituximab (see below).

The study of Harel et al. included 64 patients. (153). Only 13 patients had renal involvement, and their outcome is not specified. 28 patients (out of 64) were not initially treated mainly because of a diagnosis of “MGUS”, or because of indolent disease, and absence of severe symptoms.(153) Of these 18 never received treatment and their situation remained stable during follow-up of almost 7 years. Ten patients started therapy because of development of hematologic disease (n=3) or symptomatic CryoVasc (n=7). Fifteen patients were treated for an underlying hematologic malignancy. In 31 patients treatment was started because of symptoms related to the cryoglobulins (25 IgG subtype, 6 IgM subtype). Many patients received more than one line of therapy. Treatment regimens were variable, and consisted of alkylating agents, IMIDs, Bortezomib, HDM-ASCT, and Rituximab. Overall response rate was less than 50%, partly explained by the low response rate to monotherapy with Rituximab (3/10) and alkylating agents (6/13) in patients with IgG Cryoglobulin type I. The low response rate to Rituximab was readily explained since non-responders had a predominant plasmocytic proliferation, whereas responders all had an IgG producing Lymphoplasmocytic lymphoma. Thus, response rate was good when only considering patients who received clone directed therapy using novel agents, with a response rate of more than 70%.

Neel described 36 patients with symptomatic Cryoglobulinemia type I.(150) In only 13 patients there was no hematologic malignancy, and in only 4 of 13 kidney involvement was noted. Treatment was guided by symptoms, age, and patient preference. Thus 4 patients were not treated, and these patients had stable disease during a follow-up of 26 months. Treatment varied in the other patients, in the end 6 patients had received cyclophosphamide or Rituximab as first or second line therapy. Details of the 4 patients with kidney involvement are reported, all received therapy, with cyclophosphamide given in 3. One patient received steroid monotherapy, follow-up ended after 1 month (death due to biopsy associated bleeding). Two patients responded to therapy, although both died from pneumonia or heart failure after 70 months and 66 months respectively. The non-responding patient developed ESRD after 40 months.

Sidana reported 102 patients with Cryoglobulinemia type I, of which 39 were diagnosed as “MGUS”.(152) Treatment evaluation was possible in 21 treated patients, 5 received steroid monotherapy, 11 received an alkylating agent + prednisone, and 5 received Rituximab. An improvement in symptoms was observed in the majority of patients (4/5; 9/11; and 4/5 respectively.) Details on patients with kidney involvement are not given.

The most recent and largest study in patients with type I cryoglobulinemia, is the study from Ghembaza et al.(159) This study included 168 patients with type I Cryoglobulinemia. Of these 52 had MGRS. The authors reported data regarding treatment for 128 patients (suggesting that 40 patients were not treated or not analyzed). Subgroup analysis according underlying disease (MGRS versus hematologic malignancy) was not reported. Most patients received more than one line of therapy. Treatment regimens included corticosteroids, in most combined with cyclophosphamide (69%), rituximab (59%), and other alkylating agents (49%). Bortezomib or Immunomodulatory Drugs were used in 24% and 14% of patients respectively. Overall, complete response at 6 months was 38.7%, with no significant difference between immunoglobulins isotypes. However, relapse rate was higher in patients with IgG subtype (66% vs 34%). In patients with IgG subtype, treatment lines with novel therapies (i.e., immunomodulators and/or proteasome inhibitors) had higher 6-month complete response rate (60%–100%) as compared to alkylating agents alone (25%, 95% CI 7%–52%). Six-month complete response to treatment was greater in the patients with an IgM subtype who had received novel drug therapies (40%, 95% CI 5%–85%) or Rituximab combined with alkylating agents (47%) than in patients treated with Rituximab alone (29%). Overall survival after 1, 5, and 10 years was 97.1%, 77.0% and 52.5% respectively. Patients with IgG type I cryoglobulinemia had significantly poorer overall survival as compared to patients with IgM type I cryoglobulinemia (35.8% vs. 71.3% at 10 years, respectively). This might be related to the limited use of clone-directed therapy in this subgroup. Twenty-eight patients died during the follow-up (median 50 months), mainly from infection, clonal hematologic disorder, or heart failure. Detailed data of patients with kidney involvement are not provided, however in multivariable analysis kidney involvement was associated with lower event free survival. Interestingly, or notably, event free and relapse free survival were higher in patients with a hematologic malignancy than in patients with MGRS. The data showed that patients with IgG cryoglobulinemia I had more often renal involvement and also more often were diagnosed with MGRS. Therefore, we cannot exclude that the reported differences in response and outcome are fully explained by undertreatment in the IgG subgroup.

Plasmapheresis has been used in 15-25% of patients mostly added to cytoreductive therapy. Plasmapheresis was mainly used in patients with severe disease manifestations and particularly in patients with high cryocrit.

In conclusion: few studies report in detail the response to therapy in patients with cryoglobulinemia type I associated kidney disease. Since patients with Cryovasculitis are often older, and may suffer from severe adverse events from the treatment, the treatment must be selected based on individual patient characteristics, such as comorbidity, age, the severity of the CryoVasc, the underlying disease, and previous treatment. Some patients with cryoglobulinemia type I and limited symptoms may benefit from a wait and see strategy. In patients with kidney injury due to cryoglobulins, the main goal of therapy is to improve kidney function, reduce proteinuria, and prevent progression to ESKD. In general kidney injury attributed to cryoglobulinemia is considered a severe disease manifestation and a compelling indication for more aggressive therapy. The choice of therapy is dependent on the presence of an underlying bone marrow clone, the severity of disease (and the urgency to reverse the disease manifestations). Rituximab, alkylating agents, Bortezomib can all be considered depending on the underlying clone. It should be noted that administration of rituximab could be associated with worsening/flare of vasculitis, more frequently in patients with high levels of cryoglobulins(158, 170). (see below)

There is very limited data on treatment of patients with non-HCV type II cryoglobulinemia. In type 2 cryoglobulinemia, plasmapheresis therapy has been used successfully. Brouet already noted the effectiveness of plasmapheresis therapy in patients with acute and/or severe manifestations related to high cryoglobulin levels such as ulcera, necrosis, or acute kidney injury.(149) Nevertheless, the efficacy was not sustained, with frequent and early relapse after plasma exchange discontinuation. The relatively limited efficacy of plasmapheresis was confirmed in a retrospective study(171). This study included 159 patients treated in 22 centers. Mean age was 68 yr, and 74% were females. HCV was the cause in 71% of patients. All patients were treated with plasmapheresis, on average 10 sessions. Whereas 87% of patients received prednisone, only 50% of patients were treated with immunosuppressive therapy (rituximab in 30%, alkylating agents in 19.5%). Overall, 39% of patients with kidney involvement did not respond, and 22.5% only had a temporary improvement. Although detailed information is lacking, the data suggest that plasmapheresis as sole therapy is insufficient. Moreover, this study included many patients with HCV disease, and predated the era of very effective antiviral therapy.

There are few studies that solely or predominantly included patients with non-HCV associated type II cryoglobulinemia (table 4.2). Galli et al. reported 175 patients with mixed type II and type III cryoglobulinemia (160). Of these 96 had type II cryoglobulinemia, and no underlying cause was identified in 37 patients (‘essential’ mixed type II cryoglobulinemia). In this study only overall survival was mentioned which amounted 66% after 8 years. However, the authors did not include therapy in the analysis in view of the “broad spectrum of disorders associated with cryoglobulinemia and the variety of treatments administered”. Therefore, conclusions on treatment efficacy and kidney response are impossible.

Terrier et al. described 203 patients from the CryoVasc cohort with type II cryoglobulinemia, with no underlying cause in 95 (47%) (158). In an ancillary study the outcome in patients with mixed-cryoglobulinemia associated glomerulonephritis was described(164). This study included 80 patients with cryoglobulin-associated biopsy proven kidney disease diagnosed in the period 1995-2010. Clinical characteristics are given in table 4.3. No underlying cause was found in almost half of the patients. In all but 5 patients a monoclonal Ig was found (cryoglobulin type II). Most patients had extra-renal symptoms. In 80% of patients C4 levels were below normal. All but 4 patients had documented hematuria, 38% of patients had severe renal failure (eGFR < 30 ml/min/1.73m²), and 4 required dialysis. Half of the patients had nephrotic range proteinuria. The average follow-up was 50 months. Four patients were untreated, and of these two developed spontaneous remission, one declined therapy, and one died. Detailed information on outcome was provided for treated patients, according to initial therapy (Table 4.4). Initial treatment consisted of steroid monotherapy (n= 21), Rituximab + steroids (n=16), cyclophosphamide + steroids (n=28), or a combination of Rituximab, cyclophosphamide and steroids (n=8). Three patients received other immunosuppressants in addition to steroids and are not further discussed here. The choice of therapy was not random, and likely related to the severity of the disease, and the underlying cause (see table 4.4). Rituximab was usually given as four doses of 375mg/m^2^, cyclophosphamide was given by i.v. pulses in the majority of patients (82%), but dose and duration of therapy were not reported . Plasmapheresis was used in 33% of patients. Overall, primary non-response was documented in 17 patients (23%), most often in patients treated with steroid monotherapy. A relapse occurred in 29 patients (40%), less often in rituximab treated patients. The differences in relapse rate may be related to the underlying disease, or the dose and duration of the initial therapy. Of note, rituximab treated patients experienced more severe infections, which was attributed to the subgroup of patients who concomitantly received very high doses of steroids. Details of the 39 patients with essential mixed cryoglobulinemia at end of follow-up are described. Guided by clinical response and relapse, these patients had received different treatment schedules. Overall, 31% of patients only received steroid monotherapy, 28% Rituximab and steroids, 19% cyclophosphamide and steroids, and 17% Rituximab, cyclophosphamide and steroids. At the end of follow up (49 ± 41 months), 53% of patients were in complete remission, and 32% in partial remission. Non-response rate was 16%. Of note, renal non-response rate was higher at 29%, likely explained by patients with stable disease, no extrarenal symptoms, but insufficient improvement of kidney injury parameters. The course of eGFR can be estimated: at baseline 85% of patients had eGFR < 60 ml/min/1.73m^2^, and 44% had eGFR < 30 ml/min/1.73m^2^. At the end of follow-up (49 months), 58% had eGFR < 60 ml/min/1.73m^2^, and 34% eGFR < 30 ml/min/1.73m^2^. This improvement in eGFR is reflected in the mean eGFR, which increased from 39 ± 23 ml/min/1.73m^2^ at baseline to 48 ± 29 ml/min/1.73m^2^ at end of follow-up. In total 24% of patients had died, mainly due to severe infections or bleeding complications, which occurred in half within 1 year after start of therapy. Extra-capillary proliferation and nephrotic range proteinuria were predictors of poor kidney outcomes.

Although not proven, this study supports the initial use of rituximab-containing therapy in type 2 cryoglobulinemia with kidney involvement, although many patients will respond to steroid monotherapy or a combination of cyclophosphamide and steroids.

Patients not responding to or relapsing after Rituximab therapy often respond to renewed therapy, with best efficacy obtained by alkylating agents or a combination of Rituximab with either an alkylating agent or Belimumab.(172). Notably, infection risk was highest in patients treated with the combination of Rituximab and belimumab.

When using Rituximab as initial therapy in patients with cryoglobulinemia one must consider the risk of a flare of vasculitis, most likely caused by immune complex formation. Desbois et al. performed a retrospective study in 185 patients who received Rituximab for the treatment of autoimmune disease in the period 2005-2014 (173). A flare only occurred in patients with cryoglobulinemic disease and never in patients with other auto-immune diseases or non-cryoglobulinemic vasculitis. In patients with cryoglobulinemia flare rate was 14% (7/50), occurred on average after 8 days, and was characterized by acute kidney injury and skin manifestations. When compared with patients without flare, patients with flare more often had B cell proliferative disease, higher cryoglobulin levels, lower IgG and lower C4 levels. Few patients had received concurrent therapy with high dose steroids or plasma exchange (n=2, resp 1). All patients had cryoglobulinemia type II, consisting of IgMκ. Four of seven patients died during follow-up, all within 3 years after treatment start. Notably, kidney biopsy revealed an immune complex glomerulonephritis, and in one biopsy Rituximab was demonstrated as component in the deposits.

A similar study evaluated flare rate in 70 patients with cryoglobulinemia treated with rituximab (170). Data of 64 patients were available, 17 patients with type I cryoglobulinemia, 44 patients with type II cryoglobulinemia, and 3 patients with type III cryoglobulinemia. A flare was observed in 14 patients, and occurred 5.5 days (IQR 2-18) after Rituximab infusion. A flare was observed more often in type I (6/17) as compared to type II (7/44) and type III (1/3). A flare was characterized by variable features, mostly involving the skin (n=10; skin rash and purpura, leg ulcers), the kidney (n=6, AKI, with > doubling of serum creatinine in most), peripheral nerves (n=3), joints (n=1), gastrointestinal tract (n=1), or CNS (n=1). At the time of flare an increase in cryoglobulins and a decrease in C4 was notable, again compatible with immune complex formation. Interestingly, a flare was also observed more frequently in patients with an underlying B-cell proliferative disease. The reason is unknown, and relevant details are lacking. Notably, when comparing patients with B cell proliferative disease with and without flare, there was a large difference in serum IgM levels, with very elevated IgM levels (15.7 g/l) in patients without flare as compared to 1.3 g/l in patients with a flare. This may seem unexpected, however it is well known that immune complex formation (e.g in serum sickness) is dependent on the relative concentrations of the antigen and antibody, thus a large excess of the antibody may prevent immune complex formation.

Although the authors suggest that a flare does not indicate treatment failure and should not lead to abandon Rituximab therapy, we suggest some caution and advise to consider prevention of a flare. First many patients with a flare develop AKI superposed on CKD, and it is well known that AKI is a risk factor for future CKD, morbidity and mortality. Also, at least one patient needed acute start of dialysis and admission to the ICU. Second, it should be noted that 8/14 patients with a flare had died during follow-up (5 within 3 years). Therefore, we suggest to measure cryoglobulin levels and C4 in patients with cryoglobulinemia. In patients with high cryoglobulin levels (> 1-2 g/l) and low C4, we suggest a pretreatment with plasmapheresis, methylprednisolone, and cyclophosphamide followed by Rituximab when cryoglobulin levels have decreased. This recommendation is not limited to patients with Cryo type II, but also applies to patients with Cryo type I, specifically IgM containing cryoglobulinemia.

Table 4.1 overview of studies in patients with cryoglobulinemia type I: clinical characteristics

| Author and year | Terrier  2013 (154) | Néel  2014(150) | Harel  2015(153) | Sidana  2017(152) | Ghembaza  2023(159) |
| --- | --- | --- | --- | --- | --- |
| Study design | Retrospective  29 centers | Retrospective  2 centers | Retrospective  2 centers | Retrospective  One center | Retrospective  multicentric |
| Country | France | France | France | USA | France |
| Period | 1995-2010 | 1983-2012 | 2006-2010 | 1990-2015 | 2001-2018 |
| HCV/HBV | Excluded | Excluded | excluded | N = 8 | Excluded |
| Patients (N) | 64 | 36 | 64 | 102* | 168 |
| Age (years) | 65 (± 11) | 63 (41-85) | 62 | 59 (31-91) | 65 (56-73) |
| Gender (M/F) | 28/36 | 20/16 | 31/33 | 55/47 | 96/72 |
| **Kidney injury (%)** | **19 (30%)** | **11 (31%)** | **13 (20%)** | **14 (14%)** | **58 (35%)** |
| Skin (%) | 55 (86%) | 21 (58%) | 33 (52%) | 64 (63%) | 47 (28%) |
| Ulcers (%) | 18( 28%) | 8 (22%) | 16 (25%) | 35 (34%) | 6 (4%) |
| Neuropathy(%) | 28 (44%) | 17 (47%) | 9 (15%) | 30 (30%) | 33 (20%) |
| Joint (%) | 18 (28%) | 7 (19%) | 8 (13%) | 24% | 24 (14%) |
| Low C3  Low C4 | 36% (16/45)  81% (38/47) |  | ( 19%)9/48  (46%) 22/48 |  |  |
| Hematol.disease  - MGCS**  - Malignancy  (WM/MM/other) | 28 (44%)  36 (56%)  13/12/11 | 13 (36%)  23 (64%)  12/4/7 | 37 (58%)  27 (42%)  16/2/9 | 53 (56%)  41 (44%)  18/6/17 | 52 (31%)  116 (69%)  45/26/45 |
| Type of MIg  - IgG/IgM | NA | 11/25 | 38/26 | 50/37 | 75/93 |
| Remarks |  |  |  |  |  |
| Kidney biopsy | 18 | 10 | 9 | 13 |  |
| Histological pattern | MPGN 17 | MPGN 7 | MPGN 9 | MPGN 9 |  |
|  | C3GN 1 | TMA 2 |  | TMA 1 |  |
|  |  |  |  | Prol GN 3 |  |
| Kidney injury  AKI  Nephrotic Syndr. |  | 8  7*** | 5  8 |  |  |
| eGFR < 60 ml/min/1.73m^2^ | 33% |  | N=6 | N=11  All proteinuria |  |

Note: likely overlap of patients included in studies of Ghembaza and Terrier, not specified.

MGCS = monoclonal gammopathy of clinical significance; HM hematologic malignancy; WM = waldenström; MM = multiple myeloma; AKI = acute kidney injury; TMA = thrombotic microangiopathy; C3GN = C3 glomerulonephritis. NA =not available;ProlGN = proliferative glomerulonephritis. *in 8 patients no hematologic abnormality, in 4 HCV, in 4 no underlying disease and no detectable M-protein/abnormal FLC/cryoglobulin in serum. ** some studies report and include SMM as hematologic malignancy, in this table SMM is not included as HM, and counted as MGCS. *** nephrotic range proteinuria

Table 4.2 overview of studies in patients with mixed cryoglobulinemia: clinical characteristics

| Author and year | Galli 2017  Retrospective, multicenter  2004-2013, prevalent and incident patients, Cryo II | Terrier 2012  Retrospective, multicenter 1995-2010, Cryo type II | Terrier 2012  Retrospective, multicenter 1995-2010, Cryo type III |
| --- | --- | --- | --- |
| Patients (N) | 175 | 203 | 27 |
| Age (yrs) | 66 (55-74) | 63 (±15) | 60 (±15) |
| Gender (M/F) | 37/138 | 67/136 | 7/20 |
| Skin | 121 (69%) | 172 (85%) | 19 (70%) |
| Ulcers/necrosis | 31 (18%) | 32 (16%) | 6 (22%) |
| Neuropathy | 85 (49%) | 114 (52%) | 6 (22%) |
| Kidney | 46 (26%) | 77 (38%) | 4 (15%) |
| Arthralgia/arthritis | 140 (80%) | 84 (41%) | 8 (30%) |
| HCV | - | - | - |
| Non HCV   - Sjögren - Other AID - Hematologic - Essential - Other | Incident prevalent  16 (22%) 21 (21%)  9 (12%) 29 (29%)  3 (5%) 9 (9%)  38 (51%) 31 (31%)  8 (10%) 11 (11%) | 61 (30%)  47 (23%)  95 (47%)  - | 9 (33%)  3 (11%)  15 (56%)  - |
|  |  |  |  |
|  |  |  |  |
|  |  |  |  |
|  |  |  |  |

Adapted from (158, 160)

Table 4.3 clinical characteristics of patients with Mixed Cryoglobulin associated glomerulonephritis in one study (164)

|  | Kidney disease (n=80) | remarks |
| --- | --- | --- |
| Age (yr) | 63 (±14) |  |
| Gender (M/F) | 30/50 |  |
| Cause   - Auto-immune - Hematologic Malignancy - Essential | 18 (23%) (primary SS)  23 (29%) in 8 preceded by pSS  39 (49%) |  |
| Extrarenal disease   - Skin - Ulcers/necrosis - Neuropathy - Arthritis/arthralgia - CNS - Gut - Lung | 57 (71%)  7 (9%)  34 (43%)  28 (35%)  1 (1%)  9 (11%)  4 (5%) |  |
| Cryoglobulin type II | 75 (94%) |  |
| Low C3 and C4 | 29 (41%) |  |
| Low C4 only | 28 (40%) |  |
|  |  |  |
| eGFR (ml/min/1.73m^2^) | 40 (± 20) | 83% eGFR < 60 ml/min/1.73m^2^ |
| U protein (g/d) | 3.5 (± 3) | 49% UProt > 3g/day |
| Hematuria | 76 (97%) |  |
| Kidney biopsy features  MPGN  Mesangial proliferative  Intraluminal thrombi  Extracapillary proliferation  Interstitial infiltrates  Lymphoid nodules  Necrotizing arteritis  Immunofluorescence | 62 (93%)  5 (8%)  32 (48%)  9 (13%) (involving 12±9% of glom.)  38 (57%)  16 (24%)  11 (18%)  C3 100%; C1q in > 33%  light chain restriction in no patient | * 67 biopsies available for review |
| Remarks | 11 patients with isolated renal involvement; in 61 renal involvement at diagnosis, in 19 patients renal injury during follow-up with median interval 43 (12-156 mo)  Composition monoclonal component:  IgMκ 81%  IgGκ 9.5%  IgGλ 3.2%  Biclonal 6.3% |  |

Adapted from (164)

Table 4.4 outcome according initial first-line therapy in patients with mixed cryoglobulin associated glomerulonephritis(164)

|  | Steroids  (21) | Rituximab +steroids  (n=16) | cyclophosphamide + steroids  (n=28) | Rituximab  +Cyclophosphamide+ steroids  (n=8) | P value |
| --- | --- | --- | --- | --- | --- |
| Age (yr) | 62 ± 15 | 58 ± 16 | 64 ± 13 | 74 ± 8 | 0.05 |
| Cause (%)  Autoimmune  Hematologic  Essential | 24  5  71 | 13  37  50 | 36  29  36 | 0  87  13 | 0.001 |
| eGFR (ml/min/1.73m^2^) | 37 ± 14 | 45 ± 27 | 41± 21 | 35 ± 19 | 0.82 |
| Proteinuria (g/d) | 4 ± 3 | 3.9±4.4 | 3.3 ±2.3 | 2.3 ± 1.2 | 0.7 |
| Low C4 (%) | 84% | 71% | 92% | 83% | 0.6 |
| ***Response*** |  |  |  |  |  |
| Complete renal response (%) | 35% | 56% | 68% | 37% |  |
| eGFR month 6  (ml/min/1.73m2) | 46 ± 17 | 58 ± 23 | 51 ± 28 | 55 ± 23 |  |
| No response | 45% | 19% | 11% | 12% | 0.06 |
| Relapse | 40% | 13% | 64% | 13% | 0.002 |
| Severe infections | 15% | 38% | 11% | 38% | 0.10 |
| Death < 1 yr (%) | 5% | 25% | 4% | 38% | 0.02 |

Adapted from (164)

NB 4 patients were not treated, whereas 3 patients received prednisone and another agent.

NB: relapse includes non-renal relapse.

NB: although numerical differences, there were no statistical differences; in multivariable analysis treatment was not associated with complete renal remission; relapse rate may be dependent on underlying disease; no information on competing risk analysis (death and severe infection may affect relapse rate)

NB Final outcome including second line therapy in 73 patients: 12 steroid mono; 20 RTX + steroids; 20 CP+steroids, 21 combination therapy: 37 complete and 17 partial remissions , non -response n =18 (details unknown). Isolated low C4 associated with higher remission rate. Severe infections in 23; new onset malignancy in 5 patients

Table 4.5: clinical characteristics of patients with “seropositive” and ”seronegative” cryoglobulinemic glomerulonephritis

|  | Seropositive (N=59) | Seronegative (N=22) |
| --- | --- | --- |
| Study design | Retrospective, single center | |
| Country | USA | |
| Period | 1994-2020 | |
| HCV/HBV | Excluded | |
| Age (years) | 60 (29-84) | 64 (39-84) |
| Gender (M/F) | 22/37 | 13/9 |
| **Kidney injury (%)** | **100%** | **100%** |
| Extrarenal disease | 78% | 27% |
| Skin (%) | 64% | 23% |
| Acral ischemia (%) | 12% | 5% |
| Neuropathy(%) | 15% | 5% |
| Joint (%) | 25% | 9% |
| Low C3  Low C4 | 65%  80% | 40%  65% |
| Positive SPEP/SIF | 76% | 73% |
| Abnormal FLC | 64% | 14% |
| Underlying hem. clone  - lymphocytic  - plasmacytic  - unknown | 59%  13%  28% | 33%  22%  45% |
| Type of MIg  - IgG  - IgM | 15%  67% | 38%  31% |
| Light chain isotype  -kappa  -lambda  Biclonal | 70%  13%  18% | 35%  41%  25% |
| Kidney injury Bx |  |  |
| Histological pattern  -MPGN | 76% | 82% |
| IF  -light chain restriction  -IgM dominant | 35%  84% | 67%  50% |
| Proteinuria (g/day) | 2.8(0.1-19) | 1.8 (0.5-9) |
| Nephrotic syndrome | 27% | 22% |
| eGFR (ml/min/1.73m^2^) | 39 (4-86) | 32 (7-94) |
| AKI (%) | 50% | 73% |

adapted from (165)

**Chapter 5: Proliferative glomerulonephritis with monoclonal immunoglobulin deposits (PGNMID), includes monoclonal membranous nephropathy**

**Introduction**

In its first description, PGNMID was defined by a proliferative pattern of glomerular injury, characterized by glomerular non-organized deposits staining for a monoclonal IgG, most commonly IgG3-kappa. Recent studies have expanded this entity to include other immunoglobulin classes and also non-proliferative patterns of glomerular injury. A recent consensus report introduced the term monoclonal membranous nephropathy to discern the latter group. Since in most studies of PGNMID patients with membranous patters of injury were included, we do not discuss these entities separately. We use (P)GNMID, which includes PGNMID and monoclonal membranous nephropathy. A very recent study provided evidence that most patients with PGNMID may not represent a true monoclonal disorder. The authors show that it is important to differentiate monotypic lesions (e.g. IF staining only one light chain or heavy chain) from true monoclonal lesions ( i.e. monoclonality proven by highly sensitive techniques such as sequencing of immunoglobulin repertoire).

We summarize this study in the introduction of the supportive evidence, and discuss its implications for diagnosis and management. It is important to realize that all studies published before 2025 and summarized in this chapter have considered all patients with (P)GNMID to suffer from a monoclonal disease.

**Summary statements**

***Proliferative glomerulonephritis with monoclonal immunoglobulin deposits and monoclonal membranous nephropathy should be considered part of a spectrum of glomerular disease with monoclonal immunoglobulin deposits. Future studies should use detailed descriptive terminology.***

***Diagnosis, evaluation and management of patients with (P)GNMID should be done in (consultation with) expert centers.***

***A diagnosis of (P)GNMID requires a kidney biopsy, showing a proliferative or membranous glomerular pattern of injury by light microscopy, with non-organized glomerular deposits on EM and staining for a monoclonal immunoglobulin by IF.***

***Extra-renal organ involvement is not seen in (P)GNMID. Therefore, in patients with (P)GNMID there is no need for a routine evaluation to detect extra-renal manifestations.***

***In patients with (P)GNMID, we recommend detailed hematologic evaluation including serum and urine electrophoresis, and immunofixation, and serum FLC. A bone marrow aspirate with flow-cytometry and bone marrow biopsy to detect a lymphocytic or plasmacytic clone should always be done in patients with PGNMID and detected hematological abnormalities, and considered in MIg negative patients. A detectable MIg in serum or urine or an abnormal FLC ratio is not required to diagnose (P)GNMID.***

***In patients with (P)GNMID we recommend maximal conservative treatment as advised for patients with CKD.***

***In patients with (P)GNMID hematologic treatment decisions should take into account patient characteristics and severity of kidney injury.***

***We recommend clone-targeted treatment in treatment-eligible patients with (P)GNMID and documented bone marrow abnormalities. In selected patients without a detectable clone empirical therapy can be considered, with Rituximab preferred as first option.***

**Rationale**

***Proliferative glomerulonephritis with monoclonal immunoglobulin deposits and monoclonal membranous nephropathy should be considered part of a spectrum of glomerular disease with monoclonal immunoglobulin deposits. Future studies should use detailed descriptive terminology***

PGNMID was originally defined by the presence of proliferative glomerular patterns of injury, characterized by glomerular non-organized deposits which stained for a monoclonal IgG, most commonly IgG3κ. It is now evident that other classes of Ig and other patterns of injury such as membranous nephropathy can occur. All glomerular injury patterns associated with non-organized deposits of a MIg should be considered within the spectrum of (P)GNMID. A recent consensus report underlined the importance of detailed description and terminology, identifying and including the monotypical component, e.g PGNMID-IgG, PGNMID-IgA, PGNMID-IgM, PGNMID-LC, and monoclonal membranous nephropathy. In many patients with a diagnosis of (P)GNMID, the monotypic lesions may not represent a monoclonal disorder.

***Diagnosis, evaluation and management of patients with (P)GNMID should be done in (consultation with) expert centers.***

Rationale: (P)GNMID are very rare diseases. It is often difficult to establish a diagnosis and select the appropriate treatment. Patients with (P)GNMID will benefit from consultation with expert centers where patients are counseled by a team consisting of nephrologists, hematologists, pathologists, and laboratory specialists.

***A diagnosis of (P)GNMID requires a kidney biopsy, showing a proliferative or membranous glomerular pattern of injury by light microscopy, with non-organized glomerular deposits by EM and staining for a monoclonal immunoglobulin by IF.***

In the initial description of (P)GNMID kidney lesions were defined by a proliferative pattern of glomerular injury and the presence of non-organized glomerular deposits, composed of a complete monotypic IgG. This definition was later expanded to also include proliferative glomerulonephritis caused by deposition of monotypic IgA or IgM, as well as non-proliferative patterns of glomerular injury. By IF, (P)GNMID is typically characterized by glomerular deposits composed of a complete MIg (e.g. single heavy chain with light chain restriction) in a granular or pseudo-linear pattern. IgG subclass staining should be performed, and reported. Although IgG3κ is most common, (P)GNMID can be associated with other IgG subclasses, and with IgM, and IgA. (P)GNMID can also be associated with only a single light chain (light chain only (P)GNMID). We expect that Heavy chain only (P)GNMID might be added to the spectrum in the next years. We advise ultrastructural (EM) evaluation of the kidney biopsy, to confirm the presence of non-organized electron dense deposits, located in the mesangium, or along the glomerular capillary wall mainly in a subendothelial and less frequently in a subepithelial location. In rare cases, deposits are found exclusively in a sub-epithelial location, associated with a pure membranous pattern of injury.

***Extra-renal organ involvement is not seen in (P)GNMID. Therefore, in patients with (P)GNMID there is no need for routine evaluation to detect extrarenal manifestations.***

(P)GNMID typically is a renal-limited disease. Thus, renal progression is the only relevant outcome parameter. Treatment decisions should be primarily guided by kidney injury parameters, and the (expected) rate of progression of kidney disease.

***In patients with (P)GNMID, we recommend detailed hematologic evaluation including serum and urine electrophoresis, and immunofixation, and serum FLC. A bone marrow aspirate with flow-cytometry and bone marrow biopsy to detect a lymphocytic or plasmacytic clone should always be done in patients with PGNMID and detected hematological abnormalities, and considered in MIg negative patients. A detectable MIg in serum or urine or an abnormal FLC ratio is not required to diagnose (P)GNMID.***

Very few patients with (P)GNMID are diagnosed with an underlying hematologic malignancy. In approximately 30-40% of patients, hematologic evaluation will reveal the presence of a M-protein in serum or urine, or an abnormal FLC ratio. In these patients, bone marrow evaluation is routine. In approximately 60-70% of patients no MIg will be detected in serum or urine, and the FLC ratio is normal. The likelihood of finding bone marrow abnormalities is low in patients with (P)GNMID with normal SPEP/sIFE and normal FLC. This may be explained by the observation that many patients with (P)GNMID and no hematologic abnormality may represent a oligoclonal or polyclonal disorder, outside the spectrum of MGRS. We suggest to consider bone marrow examination including flow cytometry in patients with (P)GNMID without identified MIg, who are considered candidates for hematologic therapy based on clinical disease progression. Identification of a clonal disorder will be helpful to guide the therapeutic strategy and allow evaluation of hematologic response during follow-up.

***In patients with (P)GNMID we recommend maximal conservative treatment for kidney injury as described in chapter I.***

Patients with (P)GNMID are characterized by kidney injury. Although not specifically evaluated in this population, all patients should be treated according the guidelines for CKD.

***In patients with (P)GNMID treatment decisions should take into account patient characteristics and severity of kidney injury.***

Patients with (P)GNMID are not at risk for extrarenal complications, therefore the decision to start intensive therapy must take into account the risk of progression of kidney injury and the risk of side effects of therapy, the patient’s age, and his/her life expectancy.

***In patients with (P)GNMID at risk for progression, we recommend clone targeted treatment in patients with documented bone marrow abnormalities. In selected patients without a detectable clone empirical therapy can be considered, with Rituximab preferred as first option.***

There are no randomized trials in patients with (P)GNMID, and the case-series and cohort studies are retrospective, with treatment likely biased by patient characteristics. Most reports showed a benefit of clone-targeted therapy in patients with documented BM abnormalities. In patients without a detectable bone marrow clone, in the presence of a detectable serum M-protein, empiric therapy is often advised. There is variation between countries/centers. Some prefer initial treatment with a regimen including rituximab and steroids (with/without cyclophosphamide) whereas others use a regimen that includes Bortezomib. Although there is some logic to prefer rituximab based therapy in patients with an IgM monoclonal protein and bortezomib-based therapy in patients with a serum MIg of the IgG or IgA class, superiority of any of these regimens is not supported by the literature. The introduction of daratumumab might allow other treatment approaches. There is debate on the treatment of patients with (P)GNMID without measured hematologic abnormalities in serum, urine, or bone marrow. A recent study provided evidence to suggest that these patients do not represent MGRS. This might explain that in the literature, stable disease and even remission have been reported with conservative therapy only. The decision to start therapy should take into account the risk of progression, age, life-expectancy, and side effects of therapy. Since many of these patients may not represent true MGRS, it is advised to consider Rituximab as first line therapy in patients with progressive disease.

**Supportive evidence**.

Introduction:

Since its description in 2004 (P)GNMID has been considered a subtype of MGRS. The defining criterium was the presence of glomerular monotypic deposits (in IF positive staining for a single immunoglobulin class) in the absence of cryoglobulins. In EM the deposits could be present in the mesangium, or in a subendothelial and/or subepithelial location, and in LM glomerular lesions could be variable from mesangioproliferative, endocapillary, membranoproliferative, to purely membranous. Monotypic staining does not proof monoclonality, i.e. κ light chain restriction might reflect overproduction of oligoclonal or polyclonal IgGκ.Proof of monoclonality requires sensitive techniques. In a recent study Javaugue et al. used RNA-based immunoglobulin repertoire analysis to determine monoclonality. (2). In addition, they performed IF studies using antibodies against four different subgroups of the κ variable domains. The study included 56 patients with (P)GNMID. Serum SPEP/SIFE was positive in 9, and a bone marrow clone was found in 7. Using RNA-sequencing, monoclonality was detected in 13 patients. Thus, in 43 patients no clone was detected. In a subgroup, absence of monoclonality was confirmed by positive staining for all κ variable domain subgroups. Hematologic abnormalities were only observed in patients with monoclonal disease (serum 9/13; bone marrow 7/13). Notably, although numbers were small for most subgroups, clone detection rate differed according the immunoglobulin subclass: a clone was detected in 2/33 (6%) of IgG3κ, 2/8 (25%) of IgGλ, 2/5 (40%) of IgM, 1/4 (25%) of IgA, 3/3 (100%) of IgG1, and 3/3 (100%) of light chain only variant of (P)GNMID. Obviously these findings are relevant, and may explain the heterogeneity of patients with (P)GNMID reported in literature. In the following paragraphs, we summarize literature data, with studies that have included patients with (P)GNMID all defined as MGRS. The data must be interpreted with some caution in view of the recent findings. At the end of this chapter, we try to draw conclusions taking into account these recent findings.

***Proliferative glomerulonephritis with monoclonal immunoglobulin deposits and monoclonal membranous nephropathy should be considered part of a spectrum of glomerular disease with monoclonal immunoglobulin deposits. Future studies should use detailed descriptive terminology***

Proliferative glomerulonephritis with monoclonal IgG deposits was recognized as a separate entity in 2004 (174). Histologic evaluation showed resemblance with immune-complex glomerulonephritis, however the deposits typically stained for a monotypic IgG (mainly IgG3κ). All proliferative patterns of glomerular injury could be observed: mesangial proliferative, endocapillary proliferative and MPGN, the latter being the most frequent. Deposits were observed in the mesangium, subendothelially, and sometimes subepithelially. It is now evident that (P)GNMID can be caused by other M-proteins (e.g IgA, IgM, and even light chain only) (175-177). A recent cohort study included a patient with a suspected diagnosis of HCDD (178) However, in IF the TBM was negative. There was IgG1 positive staining in a membranous pattern. We suggest that this patient might present a form of heavy chain only (P)GNMID. Indeed, it is clear that some patients with (P)GNMID present with pure membranous nephropathy, with solely subepithelially localized monoclonal deposits (179, 180). As noted in Chapter I, monoclonal immune deposits may not always be detected in routine immunofluorescence on frozen tissue, especially in centers that receive kidney biopsies stored in transport medium. In such cases antigen retrieval using pronase/proteinase digestion may be necessary to obtain positive staining. In 2014 this entity was described as “membranous nephropathy with masked IgG-kappa deposits” (181). We suggest that most cases now should be part of the (P)GNMID spectrum. A recent consensus report of the RPS/IKMG proposed to provide detailed description of the different entities, including the pattern of injury as well as the principal component of the deposits. (5) Thus, the following terms were used: PGNMID-IgG, PGNMID-IgA, PGNMID-IgM, PGNMID-LC, and monoclonal membranous nephropathy. Further subclassification requires IgG subclass staining. It can be questioned if patients with (P)GNMID characterized by monotypic IgG3κ deposits should be considered MGRS.

***We recommend that diagnosis, evaluation and management of patients with (P)GNMID should be done in (consultation with) expert centers.***

(P)GNMID are rare diseases. In the original study, the incidence of (P)GNMID in native kidney biopsies was only 0.21% (174). Patients will benefit from consultation with expert centers, to discuss diagnosis and treatment.

***A diagnosis of (P)GNMID requires a kidney biopsy, showing a proliferative or membranous glomerular pattern of injury by light microscopy, with non-organized glomerular deposits on EM and staining for a monotypic immunoglobulin by IF.***

By light microscopy, proliferative and non-proliferative patterns of glomerular injury can be found (Table 5.2). A diagnosis of (P)GNMID requires positive staining in IF for a monotypic protein (intact immunoglobulin or light chain only), in the mesangium and/or the capillary wall. The deposits are non-organized in EM, and can be located in all glomerular compartments, and are rarely limited to one compartment only (174, 179). Identification of the culprit monotypic protein may require studies on pronase/proteinase treated tissue, especially when biopsies are stored in transport medium (see Chapter I)(181). The mere finding of a monotypic immunoglobulin in IF is not sufficient to diagnose (P)GNMID. (P)GNMID should be differentiated from amyloidosis, MIDD, immunotactoid GN, and type I cryoglobulinemic glomerulonephritis. Diagnosis requires nephropathological expertise and study of the kidney biopsy with all techniques (LM with appropriate and specific staining; IF and/or IHC with appropriate antibodies, including IgG subclass staining, and preferably electron microscopy).We strongly advise IgG subclass staining in patients in whom the diagnosis of (P)GNMID is solely based on the presence of light chain restriction in the kidney biopsy, and certainly in patients without any confirmatory hematologic abnormalities in serum or urine.

***Extra-renal organ involvement is not seen in (P)GNMID. Therefore, we in patients with (P)GNMID there is no need for recommend routine evaluation to detect extrarenal manifestations.***

(P)GNMID are renal limited diseases, therefore routine evaluation of other organs is not necessary. Outcome is determined by the severity of kidney injury.

***In patients with (P)GNMID, we recommend detailed hematologic evaluation including serum and urine electrophoresis, and immunofixation, and serum FLC. A bone marrow aspirate with flow-cytometry and bone marrow biopsy to detect a lymphocytic or plasmacytic clone should always be done in patients with PGNMID and detected hematological abnormalities, and considered in MIg negative patients. A detectable MIg in serum or urine or an abnormal FLC ratio is not required to diagnose (P)GNMID.***

In patients with (P)GNMID the pathognomomic abnormality is the presence of a monoclonal protein in the glomerular compartment, assessed by appropriate IF staining. The detection rate of a hematologic malignancy, a circulating MIg or a small bone marrow clone differs according to the subtype of (P)GNMID. In patients with light chain-only (P)GNMID a hematologic malignancy was present in 5/17 (29%) patients, a serum monoclonal light chain in 13 (76%), an abnormal serum free light chain ratio in 10/12 patients (83%), and in parallel bone marrow abnormalities were detected in 15/16 patients (94%)(175). In contrast, in most studies that report “classical” (P)GNMID few patients are diagnosed with an underlying hematologic malignancy (3-7%; Table 5.1). An exception is the study of Guiard et al., in this study 35% of patients had a hematologic malignancy (182). This study is also notable, because in this study the majority of patients had a membranous pattern of glomerular injury (54%; see Table 5.2). A possible explanation for these apparent discrepancies is suggested below.

A MIg was found in 30-37% of patients with classical (P)GNMID, and in 9/10 patients without abnormal SPEP/sIFE, FLC ratio was also normal (Table 5.1). Although small bone marrow clones were predominantly observed in patients with a detectable M-protein or abnormal sFLC ratio, bone marrow abnormalities have been found in few patients without detectable monoclonal IgG. Gumber et al. reported 19 patients with (P)GNMID, an M protein was found in 7, and in 4 of these a bone marrow clone was found; on the other hand, 2 patients had a bone marrow clone, but no detectable M protein (180). Similarly, in the study of Kousios including 14 patients, a BM clone was found in 2/5 patients with a detectable M protein, but there were 3 patients with a BM clone but no detectable M protein (176). Admittedly, results of FLC assay were often not reported, thus, it is unknown if these patients had a normal FLC ratio. Another study questioned the relevance of bone marrow evaluation in selected patients (69). These authors evaluated the accuracy of hematologic testing in patients with (P)GNMID. This study included 60 patients, mean eGFR 36 ml/min/1.73m^2^, proteinuria 3.6 g/day. Serum immunofixation was positive in 12 patients, in 4 confirmed by abnormal к/λ ratio. In addition, there were 8 patients with an abnormal FLC assay, without abnormal serum electrophoresis. In 40 patients bone marrow was evaluated, including flow cytometry. Abnormal BM findings were found in 100% of patients with abnormal SPEP and FLC ratio (3/3), in 75% of patients with abnormal SPEP and normal FLC ratio (6/8), in 16% of patients with abnormal FLC ratio only (1/6) and *in none of the patients with normal SPEP and FLC ratio* (0/23). This study thus suggests that in patients with (P)GNMID, no detectable M-protein, and normal FLC the value of added BM studies is negligible. Vignon et al. also did not find a BM clone in patients without detectable M-protein (177). Since bone marrow evaluation is rarely abnormal if at all in patients with (P)GNMID, negative SPEP/SIF and normal FLC ratio, the decision to perform detailed hematologic and bone marrow evaluation in these patients should be balanced, in particular in view of the evidence that not all (P)GNMID may represent MGRS (see below). We suggest to consider bone marrow studies in patients who are advised to start treatment with Rituximab or other agents.

Although in the majority of patients the culprit M-protein is of the IgG3κ class, the study of Guiard et al. suggested an association between the pattern of injury and the IgG subclass: in patients with MPGN and positive IgG staining, IgG3 was found in 80%, in contrast in patients with a membranous pattern of injury IgG3 was observed in only 18%, with IgG1 being dominant (64%) (182). In one study, mainly including patients with a proliferative pattern of injury, an association between the presence of an M-protein and the IgG subclass was found: an M-protein was present in only 2/21 patients with IgG3 subclass staining, in 5/9 with IgG1, and 2/2 with IgG2 (179). These findings fit with the recent findings of Javaugue et al. (2). This study reported a low incidence of “true” monoclonal disease in patients with IgG3κ associated PGNMID, whereas monoclonality was the rule in patients with IgG1 or light chain only variants of PGNMID. Still, even in the latter study in 4/13 patients with a detected clone serum and bone marrow studies were normal. Therefore, when using routine techniques, absence of a hematologic abnormality cannot be used to definitely rule out monoclonal disease.

***In patients with (P)GNMID we recommend maximal conservative treatment for kidney injury as described in chapter I.***

Although there is no evidence, we suggest that all patients with kidney injury and (P)GNMID should be treated according CKD guidelines.(KDIGO CKD)

***In patients with (P)GNMID treatment decisions should take into account patient characteristics and severity of kidney injury.***

Patients with (P)GNMID do not develop M-protein related extrarenal complications. The outcome is mainly determined by the risk of progressive kidney injury. Patients with (P)GNMID are often elderly, and many do not present with any hematologic abnormality. The literature data on treatment and outcome is mainly based on retrospective studies, which included a limited number of patients, were uncontrolled, with treatment decisions likely biased and based on clinical characteristics(175-177, 179, 180) (183).Treatment decisions should balance risks and benefits, taking into account the (estimated) progression of kidney injury, the presence of hematologic abnormalities, the risks of therapy, the age of the patient, comorbidities, and life expectancy. In the following paragraph a detailed description of literature studies is given.

***We recommend clone-targeted treatment in patients with (P)GNMID and documented bone marrow abnormalities. In selected patients without a detectable clone empirical therapy can be considered, with Rituximab preferred as first option***

Recent studies that describe the characteristics and outcome of patients with (P)GNMID in larger patient cohorts are summarized in tables 5.1 and 5.3. The studies differed in patient selection: The 2009 study of Nasr et al. only included patients with classical (P)GNMID (IgG only), the studies of Guiard et al., Gumber et al. and Kousios et al. included patients with IgG- and non-IgG-associated (P)GNMID, the study of Vignon et al. only included patients with (P)GNMID characterized by pure IgA monoclonal deposits, the study of Bu et al. only included patients with the IgM variant of (P)GNMID, whereas the study of Nasr et al. published in 2020 only included patients with (P)GNMID characterized by light chain-only deposits (175-177, 179, 180, 182, 184). The most recent study specifically evaluated the outcome in patients with (P)GNMID and no detectable clone (including negative M-protein screening and normal FLC) (178). The data underline that (P)GNMID is a rare disease, with in these large centers an incidence of 1-2 patients per year. Most patients with (P)GNMID presented with severe kidney injury, hematuria, and variable degree of proteinuria. A nephrotic syndrome was observed in 15-56% of patients. The reported studies were all retrospective, and included patients who were seen over a period of 10-20 years. Not unexpectedly, treatment has been quite variable (Table 5.3). Many authors prefer clone-directed therapy, based on the efficacy of clone directed therapy in retrospective studies. However, the treatment and outcome data should be interpreted with caution, since treatment was not controlled, and clone-directed therapy was by definition only used in patients with a documented bone marrow clone. Some studies reported results of “empirical therapy” in patients without detectable clone, consisting of rituximab + steroids (+/- cyclophosphamide), cyclophosphamide +/- steroids, or bortezomib +/- dexamethasone. Also, conservative therapy has been used, more often in patients without a detectable M-spike, and more likely in patients with less severe kidney injury. Of note, stable disease (even with partial or complete remission) has been described in patients treated with conservative therapy only.(185) (Table 5.3).

We provide a narrative review of the studies. Of note, these studies were reported well before the abovementioned publication of Javaugue and colleagues. It is likely that a diagnosis of MGRS does not apply to many patients included in these studies.

The oldest study included 32 evaluable patients (179). Overall outcome was unfavorable, with only 37.5% of patients developing complete or partial remission. Few patients were treated with the newer and currently used drugs such as bortezomib or immunomodulatory agents. Almost half of the patients (14/32) were not treated (n=5) or treated with RAAS blockade only (n=9). Most untreated patients presented with severe renal insufficiency and showed severe IFTA on kidney biopsy. The remaining patients either received steroid monotherapy, a combination of an alkylating agent with steroids, or rituximab or mycophenolate mofetil based therapy. Obviously, treatment was biased. Unfortunately, there is no information of the baseline characteristics in untreated and treated patients. Still, 9 out of 10 patients with an M-spike at diagnosis received some form of hematologic therapy, whereas only 9 out of 22 patients without an M-spike did. Although one could argue that the relatively low remission rate of 37.5% could be related to the lack of hematologic therapy, or the use of less active hematologic therapy, it is notable that 4 out of 9 patients treated with RAAS blockade only developed complete or partial remission. In multivariable analysis, only the percentage of global sclerosis was associated with development of ESKD, and treatment was not. The latter could reflect confounding by indication.

Guiard et al. reported 26 patients including 9 in whom a hematologic malignancy had been diagnosed (182). Most patients with a malignancy received appropriate hematologic therapy. A complete renal remission was obtained in 5/9 of these patients, associated with a hematologic response in all these patients. There were three patients without hematologic malignancy, but a detectable M-spike. Two patients received cyclophosphamide and steroids and developed complete remission. Patients who relapsed after CR, all responded to renewed therapy. Treatment was variable in 14 patients without a malignancy or M-spike. Five patients received either cyclophosphamide or Rituximab, followed by complete remission in four. In contrast, nine patients were untreated (n=5) or treated with prednisone monotherapy (n=4). Five patients developed ESRD or died, still a complete remission was observed in 2 patients.

Gumber *et al.* reported overall good outcome in patients with (P)GNMID using “clone-directed” therapy in 4 patients with a bone marrow abnormality, non-directed therapy in two patients with a clone, or empirical therapy, consisting most often of rituximab and steroids (+/- cyclophosphamide) in 10 patients without a detectable clone (180). Only two patients received empirical initial therapy with bortezomib. Overall, 13/16 patients responded with a complete or partial renal remission. No patient developed ESKD (median follow up was only 693 days after diagnosis). This study thus suggests good outcome with more aggressive therapy. Of note, this study included patients with lower UPCR and better preserved eGFR. Importantly, in this study 9 patients who received empirical therapy with rituximab (with/without cyclophosphamide and steroids) were positive for IgG M-protein staining, and 5 developed persistent remission suggesting that Bortezomib might not always be necessary therapy in patients with an IgG-associated (P)GNMID. During follow-up seven evaluable patients were retreated for relapse ((n=5) or non-response (n=2), most with a regimen including Bortezomib. A response was noted in five patients (CR 3, PR2).

Kousios et al. reported 14 patients with (P)GNMID (176). A bone marrow clone was detected in five patients. Clone directed therapy in two patients resulted in complete or partial kidney response. Three patients received no treatment. Somewhat unexpected, one patient was adjudicated as complete renal reponse. However, this patient presented with normal eGFR and low grade proteinuria (UPCR 36 mg/mmol). In this study complete response was defined by stable eGFR and reduction of proteinuria to less than 50 mg/mmol. Thus this patient already fulfilled the CR criteria at baseline. Moreover follow-up was short (11 months). Three patients with detectable M-protein were treated with either Rituximab (n=2, both NR), or Bortezomib (n=1, CR). Finally, six patients without M-protein received Rituxumab (n=3, all PR), cyclophosphamide (1, PR), steroids monotherapy (1, CR), or no treatment (1, NR).

Vignon *et al.* described 19 patients with (P)GNMID characterized by monoclonal IgA deposits.(177) Four patients (without M protein) only received conservative therapy, with three patients having stable kidney function during 18 months follow-up. Six patients without M-protein received treatment mainly consisting of steroids and/or cyclophosphamide. A major renal response was observed in 1, and stable kidney function in three. Rituximab as second line therapy in two patients failed. Finally, 6 patients received intensified therapy (Bortezomib, alkylating agent, or immunomodulatory drug based). In 4 patients with a M protein, hematologic response was CR in 1, VGPR in 3. All six patients developed a major renal response. While the authors suggest that the data suggest superiority of “clone-directed” or anti-myeloma therapy, a cautious note is warranted. Clearly treatment was biased toward patients with a detectable M-spike. Moreover, in patients without an M-protein renal function has remained stable in many, at least for a period of 1-2 years.

Bu et al. described 23 patients with (P)GNMID and monotypic IgM staining (IgMκ in 12 and IgMλ in11). All patients were considered MGRS (including 3 with low grade chronic lymphocytic leukemia or small lymphocytic lymphoma). Interestingly, a M-protein was detected in 50% of patients, however in only half of these the detected M-protein was compatible with the nephropathic IgM. Four patients received symptomatic therapy, 4 patients were treated with steroids only, whereas 15 patients received hematologic therapy (including anti-B cell therapy in all but one). Overall, complete and partial remission rate was low (3 patients), and 12 patients progressed to ESKD. ESKD free survival was associated with lower initial and peak serum creatinine, and lower degree of interstitial fibrosis. Some patients attained remission or had stable eGFR (with persistent moderate proteinuria which may simply reflect the often present glomerulosclerosis) independent of therapy, with some response noted in 1/4 patients treated conservatively, in 2/4 patients treated with steroids only, and in 5/13 patients treated with anti-B cell therapy.

Terashita M et al. analyzed the outcome of patients with MGRS and undetectable clone (178). This retrospective study analyzed 29 patients with a diagnosis of MGRS, with negative SIFE, normal FLC (eGFR corrected) and normal bone marrow biopsy. Mean age was 58 yr, 52% were male, mean serum creatinine 1.8mg/dl (159 umol/l), and proteinuria 4.6 g/day. Kidney biopsy revealed a MPGN pattern of injury in most patients (18/29). A diagnosis of (P)GNMID was made in 28 patients. This is not unexpected since in most patients with other MGRS subtypes hematologic abnormalities are found. In one patient a diagnosis of HCDD was made, although this diagnosis can be questioned since staining of the TBM was negative. In fact, this patients had IgG1 positive staining in a membranous pattern, thus this may present a form of heavy chain only (P)GNMID. Notably, in the majority of patients (26/29) the involved M-protein (detected in IF) was of the IgG subclass. Initial therapy varied (conservative in 8, plasma cell targeted in 6, B-cell targeted (Rituximab) in 7, and non-specific immunosuppressive therapy in 8 (in 5 steroid monotherapy). Although overall outcome was reasonable, with complete remission in 9 patients and partial remission in 8 patients (of 24 patients who were evaluable with initial UPCR > 0.5g/g), it is important to realize that in most patients treatment was adjusted to the initial response, thus the majority of patients received two or more lines of therapy. Obviously there is bias since initial treatment was based on clinical characteristics (low UPCR in patients on conservative therapy), and non-response was followed by initiation of second line therapy. Median follow-up was 55 months. In 24 patients with initial Uprot > 0.5g/day, outcome could be analyzed according initial therapy: 4 patients were treated with conservative therapy only (2CR, 2NR), 2 patients received cyclophosphamide (2CR), 6 patients received B-cell targeted therapy (1CR and 3PR), and 12 patients needed plasma-cell directed therapy (4CR, 3PR). During follow-up 7/26 patients with (P)GNMID in native kidney developed ESKD. Side effects were more frequent in patients treated with anti-plasma-cell therapy. Although retrospective and uncontrolled this data suggest that the choice of treatment requires a careful decision making: response to conservative therapy might occur in patients with minimal proteinuria; sequential therapy is often needed, however starting with B-cell targeted therapy can be considered, with switch to plasma cell therapy in non-responders. The heterogeneity in response seems compatible with the finding that in many patients the disease is not caused by a monoclonal protein.

Although patients with light chain-only (P)GNMID present with similar clinical characteristics (low GFR, hematuria, severe proteinuria), there are remarkable differences (table 5.1). In the series reported by Nasr *et al.* 5/17 were diagnosed with a multiple myeloma (175). Treatment in these five patients resulted in complete hematologic response, and either complete (n=3) or partial (n=2) renal response. Results were different in patients with no hematologic malignancy. Three patients with increased FLC/positive M protein (thus allowing assessment of hematologic response) received Bortezomib, with variable response (hematologic 1CR, 1PR, 1NR; renal 1 PR, 2NR). Seven patients with non-evaluable hematologic response (often no FLC analysis done) were treated with low intensity therapy (steroids, MMF, only 1 Bortezomib). Most progressed to ESKD. It is likely that in these patients the diagnosis of light chain only (P)GNMID was made in retrospect, which explains the missing FLC data. The poor outcome likely reflects insufficient therapy in patients with a known hematologic abnormality.

The introduction of daratumumab has extended the armamentarium of hematologists, and the drug has been used in MM and AL-amyloidosis (see Chapter II). Recent pilot studies have suggested benefit of Daratumumab in patients with (P)GNMID. However, its role in (P)GNMID is not yet defined, in the absence of large trials or larger cohort studies. Zand et al. performed a phase 2 study in 10 patients with (P)GNMID who received Daratumumab (186). Only 1 patient had a detectable monoclonal immunoglobulin in blood and urine. In seven patients Daratumumab was the initial therapy, three patients had received prior therapy with Rituximab or MMF. All patients experienced an improvement of proteinuria after 6 and 12 months (baseline 4.3g/24h, 6 months 0.7 g/24h, 12 months 1.3 g/24h), with stable eGFR (which was notably quite high at 61 mL/min/1.73m^2^ compared to other studies). Safety profile was acceptable, but 2 patients were withdrawn from the study due to severe adverse event (severe headache and closed-angle glaucoma). At 12 months follow-up, three patients were nephrotic, mainly due to early relapse (Daratumumab was stopped after 26 weeks). Although this study thus suggest efficacy of daratumumab as initial therapy in (P)GNMID, it is unclear if responses are long-lasting, and within the short follow-up of 6 months after last infusion relapse rate was 30%. Also, there is no comparison with the “standard” plasma cell targeted therapy (bortezomib based).

Almaani et al. described 5 patients with “treatment resistant” (P)GNMID who received Daratumumab (187). In only one patient a monoclonal component was detected. Noteworthy, three patients in this study were very young (< 29 , 29 and 30 years). Three patients did not respond well, and developed ESKD or remained nephrotic. There were two patients with a renal response. Still, the observation period after start of Bortezomib based therapy was short, respectively 1 and 7 months, and it is unclear if renal response was solely attributable to daratumumab. In conclusion, rescue therapy with Daratumumab may not be very effective, and there is no data to suggest superiority over the routinely used therapies.

The variation in treatment and response in the above studies may well be explained by the findings that the majority of patients with PGNMID, especially in PGNMID-IgG3k, do not fulfil the criteria for MGRS (no monoclonality proven using sensitive techniques). Since these techniques are not readily available for routine use, we suggest that the treatment advise can be maintained: treatment should only be considered in patients with severe/progressive kidney disease; treatment should be clone directed in patients with a documented bone marrow clone; in patients without a clone empiric therapy with Rituximab is advised as first line therapy (comparable to treatment of polyclonal fibrillary GN). In patients with (P)GNMID who do not respond to Rituximab, additional therapy should be considered targeting the plasma cell or B cell. We strongly suggest that the latter patients should be referred to expert centers, which are able to study monoclonality with the novel, sensitive techniques.

Table 5.1 Overview of studies of patients with (P)GNMID: clinical characteristics

|  | Classical (P)GNMID | | | | |  |  | Light chain only (P)GNMID |
| --- | --- | --- | --- | --- | --- | --- | --- | --- |
| Author  Year | NASR  2009  (179) | Guiard 2011  (182) | Gumber  2018  (180) | Kousios  2019 (176) | Vignon  2017  (177) | Bu*  2025  (184) | Terashita*  2023  (178) | NASR  2020(175) |
| Country | USA | France | USA | UK | France | US | USA | USA/France |
| Period | 1999-2009 | 1980-2008 | 2000-2016 | 2006-2017 | 1998-2015 | 2009-2023 | 2010-2022 | NA |
| Patients (N) | 37 | 26 | 19 | 14 | 14 | 23 | 29 | 17 |
| Age (yr) | 55  (20-81) | 52  (29-77) | 58  (25-83) | 65  (25-76) | 55  (33-75) | 72  (28-87) | 58  (47-66) | 62  (44-84) |
| Gender (M/F) | 14/23 | 10/16 | 12/7 | NA | 9/5 | 18/5 | 15/14 | 13/4 |
| Screat (umol/l) | 245  (62-1500) | 211 ±190 | 159*  (106-353) | NA † | 138  (71-275) | 168  (53-990) | 159  (115-238) | 177  (80-504) |
| Salb (g/l) | 31  (11-49) | 26 ± 7 | NA | NA | 34  (19-41) | NA | NA | NA |
| UPCR (g/day) | 5.7  (0.36-17) | 5.3 +/-4.6 | 3.6*  (2.3-8.0) | 5.1**  (0.36-10) | 4.2  (0.4-7.8) | 3.1  (0.3-25.1) | 4.6  (2.3-7.9) | 4.7  (2-12) |
| Hematuria (%) | 77% | 87.5% | NA | NA | 93% | 91% | NA | 94% |
| Nephrotic Syndrome (%) | 49% | 85% | NA | NA | 15% | 36% | NA | 56% |
| Low C3 | 19% | 23% | NA | NA | NA | 25% | NA | 50% |
| **Monoclonal Ig** |  |  |  |  |  |  |  |  |
| (SPEP/SIFE + urine) | 30% | 31% | 37% | 36% | 36% | 27%& | 0% | 76% |
| Abnormal FLC | 1/4  (tested in normal SPEP) | N/A |  | 2 (with normal SPEP, all tested??) | 0/6  (in normal SPEP) | 26% | 0% | 10/12  (1/2 with normal SPEP) |
| Hematologic malignancy | 3%  MM 1 | 35%  MM 2  CLL 4 NHL 3 | 5%  1 | -- | 7%  MM 1 | 0% | 0% | 29%  MM 5 |
| Bone marrow  Abnormalities | 2  (9.1%)# | 9  (35%) | 6  (32%) | 5  (36%) | 3  (21%) | 4  (26%) | 0% | 15  (94%)## |

Values are mean (±SD ) or median (range or *IQR); NA =not available; † 9 patients with eGFR < 60 ml/min/1.73m2; of these 4 with eGFR < 30 ml/min/1.73m2

**UPCR in g/10 mmol

# % of 22 tested patients; ## % of 16 tested patients

& abnormal SPEP/SIFE in 50%, but in many patients SPEP/SIFE identified another uninvolved M-protein

Table 5.2 Overview of studies in (P)GNMID: Kidney biopsy characteristics

| Author  Year | | NASR  2009  N=37 | Guiard 2011  N=26 | Gumber  2018  N=19 | Kousios  2019  N=14 | Vignon  2017  N=14 | Bu  2025  N=13 | Terashita  2023  N=29 | NASR  2020  N=17 |
| --- | --- | --- | --- | --- | --- | --- | --- | --- | --- |
|  | **Predominant LM pattern of injury** | | | | | | | | |
| MPGN | | 57% | 46% | 95%&& | NA | 43% | 83% | 62% | 76% |
| Endocapillary | | 35% | 0% | 5% | NA |  | 4% | 10% | 18% |
| Mesangial Proliferative | | 3% | 0% | 5%* | NA | 50% | 13% | 22% |  |
| Membranous | | 5% | 54% | 0%* | NA | 7% | 0% | 10% |  |
| Crescents | | 32% | 50% | NA | NA | 71%  (6-40%) | 9% | NA | 14% |
| Crescents (>50%) | | 5.4% | NA | NA | NA | 0 | 0% | NA | 6% |
| **IF staining** | |  |  |  |  |  |  |  |  |
| -IgG | | 100% | 81% | 89% | 79% | - | 0% | 90% | 0 |
| -IgG3$ | | 66%$$ | 48%$$$ | 50%# |  | - | - | 45% | - |
| -IgG1 | | 28% | 38% | 50%# |  | - | - | 31% | - |
| -IgM | |  | 0% | 11% | 14% | - | 100% | 0% | 0 |
| -IgA | |  | 0% |  | 7% | 100% | 0% | 10% | 0 |
| κ | | 73% | 81% | 58% | 50% | 50% | 52% | 62% | 71% |
| λ | | 27% | 19% | 42% | 50% | 50% | 48% |  | 29% |
|  | |  |  |  |  |  |  |  |  |
| C1q | | 64% | NA | NA | NA | NA | 17% | NA | 6% |
| C3 | | 97% | NA | NA | NA | 78% | 96% | NA | 100% |

$ subclass as percentage of stained and IgG positive biopsies $$ 32 biopsies stained for subclass # only 8 biopsies stained for IgG subclass; && in many biopsies additional patterns of injury, eg endocapillary and membranous. 1 biopsy showed combined endocapillary and mesangial proliferative pattern of injury; $$$IgG subclass distribution is dependent on pattern of injury: MPGN 80% IgG3, in MN only 18% IgG3.

NA data not provided

Most studies indicate that there are often combined patterns of injury e.g. in MPGN there is often endocapillary proliferation. In many biopsies there is a membranous pattern of injury. In NASR 2009, deposits were found subendothelially (100%), in the mesangium (>96%), and also subepithelially (~57%). The subepithelial deposits are often only segmental.

Table 5.3 Overview of studies in (P)GNMID: outcome

| Author  Year | NASR  2009 | | Guiard 2011 | | Gumber  2018 | | Kousios  2019 | Vignon  2017 | BU  2025 | Terashita  2023 | NASR  2020 |
| --- | --- | --- | --- | --- | --- | --- | --- | --- | --- | --- | --- |
| Patients with follow up (N) | 32 | 25 | | | 19 | | 14 | 14 | 23 | 24@ | 15 |
| Patients treated with any “hematologic” therapy | 18 | 20 | | | 16 | | 10 | 10 | 19 | 20 | 14 |
| Clone directed and/or Rituximab/Bortezomib  or thalidomide | 7 | 12^ | | | 12 | | 8 | 6## | 15 | 18 | 9 (incl 5 MM) |
|  |  | | |  | | Overall outcome | | | | | |
| Complete remission | 5*** | 13 | | | 6 | | 4 | 7# | 1 | 9 | 3 |
| Partial remission | 8* |  | | | 7 | | 5 |  | 3 | 6 | 2 |
| Persistent renal dysfunction | 12 |  | | | 3 | | 3 |  | 7 |  | 2 |
| ESKD | 7** | 6 | | | 0 | | 2 | 3 | 12 | 7 | 8 |
| Death | 5 | 2 | | |  | |  |  | 6 | NA |  |
| Persistent hematuria | 1 |  | | |  | |  |  |  | NA |  |
|  |  | | |  | | outcome in patients not treated with chemotherapy/Rituximab/Bortezomib | | | | | |
| Untreated  (includes RAS only) | 14 | 5 | | | 3& | | 4 | 4 | 4 | 4 | 1 |
|  | 3CR | 1 CR | | |  | | 1CR$ |  |  | 2CR |  |
|  | 2PR |  | | |  | |  |  | 1 PR |  |  |
|  | 4 ESRD | 3 ESRD | | | 3  ESRD | |  |  |  |  |  |
|  |  |  | | |  | |  | 3 “stable” after 18 months FU |  |  | Persistent dysfunction |

* 1 died, **4 died, *** 1 patient had persistent hematuria

$ presented with minimal proteinuria

&untreated because of severe renal insufficiency and moderate to severe scarring on biopsy

## 2 patients received Bortezomib as third line therapy after having failed steroids and Rituximab (see text)

# CR = major renal response defined by UPCR < 1g/day and 30% increase eGFR

^9 patients with Hematologic malignancy were treated with various regimens + 3 patients without received Rituximab. No patient received Bortezomib

NB in evaluable patients: renal response is only observed in patients with hematologic response

@ evaluated 24 patients with initial Uprot > 0.5g/day

**Chapter 6: Immunotactoid glomerulopathy.**

**Introduction**

Immunotactoid glomerulopathy is a rare glomerular disease, characterized by the presence of Congo red-negative, immunoglobulin-derived, organized deposits with a microtubular structure in the mesangium and glomerular capillary wall. The term GOMMID is sometimes used (glomerulonephritis with organized microtubular monoclonal immunoglobulin deposits). In most patients, but no all, the deposits are composed of a monoclonal immunoglobulin.

**Summary statements**

***Diagnosis, evaluation and management of patients with immunotactoid glomerulopathy should be done in (consultation with) expert centers.***

***A diagnosis of immunotactoid glomerulopathy requires a kidney biopsy, showing glomerular patterns of injury, with positive staining of glomerular deposits for IgG, and organized microtubular deposits in EM. Perform pronase/protease immunofluorescence microscopy as salvage technique in immunotactoid glomerulopathy when there is inadequate tissue or only weak Ig staining on routine immunofluorescence microscopy***

***In patients with immunotactoid glomerulopathy, and no underlying disease, we recommend hematologic evaluation, independent of the clonality of the IgG deposits (monoclonal or polyclonal). The evaluations should include the search for a MIg in serum or urine, and bone marrow aspirate with flow-cytometry, and bone marrow biopsy***

***In patients with immunotactoid glomerulopathy we recommend conservative treatment for kidney injury as described in chapter I.***

***We recommend clone targeted treatment in selected patients with immunotactoid glomerulopathy and documented bone marrow abnormalities. We suggest “empirical” therapy in patients with MIg associated immunotactoid glomerulopathy and undetectable bone marrow clone with treatment initiation dependent on comorbidity, age and severity of kidney injury.***

***We suggest “empirical” therapy as used for monoclonal immunotactoid glomerulopathy in patients with polyclonal ITG and progressive kidney disease.***

**Rationale**

***Diagnosis, evaluation and management of patients with immunotactoid glomerulopathy should be done in (consultation with) expert centers.***

Immunotactoid glomerulopathy (ITG) is a very rare disease. It is often difficult to establish a diagnosis and select the appropriate treatment. Patients with ITG will benefit from consultation with expert centers where patients are counseled by a team consisting of nephrologists, hematologists, pathologists, and laboratory specialists.

***A diagnosis of immunotactoid glomerulopathy requires a kidney biopsy, showing glomerular patterns of injury, with positive staining of glomerular deposits for IgG, and organized microtubular deposits in EM.***

A diagnosis of immunotactoid glomerulopathy (ITG) can only be made with EM evaluation of a kidney biopsy. EM studies should show the characteristic microtubules, often in parallel arrays, in the mesangium and the capillary wall. In light microscopy, different glomerular patterns of injury can be recognized: endocapillary proliferative pattern, membranoproliferative pattern and membranous pattern, with considerable overlap. A purely membranous pattern of injury is rarely observed. The deposits stain positive for IgG in IF. Typically, light chain restriction is observed in the majority of patients, thus confirming ITG as a subtype of MGRS.

**Perform *pronase/proteinase immunofluorescence microscopy as salvage technique in ITG when there is inadequate tissue or only weak Ig staining on routine immunofluorescence microscopy***

Pronase/protease immunofluorescence microscopy is useful in kidney biopsies lacking glomeruli in the frozen tissue sample. Also, it might be an unmasking technique in kidney biopsies which show negative or weak IgG staining, especially in centers that use transport medium for shipment of kidney biopsies (see Chapter 1).

***In patients with immunotactoid glomerulopathy, and no underlying cause, we recommend hematologic evaluation, independent of the clonality of the IgG deposits (monoclonal or polyclonal). The evaluations should include the search for a MIg in serum or urine, and bone marrow aspirate with flowcytometry, and bone marrow biopsy .***

Although rare, ITG has been described secondary to HIV and HCV infections, and such underlying causes should be excluded. In the majority of patients with ITG a monoclonal immunoglobulin can be found in serum or urine, and an underlying hematologic malignancy is also often present. Although the incidence of hematologic abnormalities is higher in patients with monoclonal ITG, abnormalities are detectable in approximately 25% of patients with polyclonal ITG. Therefore, we suggest to perform hematologic evaluation in all patients with ITG, independent of the clonality of the IgG deposits.

***In patients with Immunotactoid glomerulopathy we recommend conservative treatment for kidney injury as described in chapter I.***

Although there is no evidence specific for patients with ITG, we advise all patients be treated according guidelines for CKD.

***We recommend clone targeted treatment in selected patients with immunotactoid glomerulopathy and documented bone marrow abnormalities. We suggest “empirical” chemotherapy in patients with MIg associated immunotactoid glomerulopathy and undetectable bone marrow clone with treatment initiation dependent on comorbidity, age and severity of kidney injury.***

Many patients with ITG will develop ESKD without therapy. In patients with ITG and an identified bone marrow clone, therapy targeting the abnormal clone is advised. In patients with ITG with monoclonal immunoglobulin deposits (with or without M-protein or abnormal FLC ratio) empirical therapy is advised, and start of therapy should be weighed against age, comorbidity, and life expectancy. Not supported by evidence we suggest Rituximab as first line option for empirical therapy, as described for PGNMID (chapter V)

***We suggest “empirical” therapy as used for monoclonal ITG in patients with polyclonal ITG, and progressive kidney disease.***

There is no data to support treatment advise for patients with polyclonal ITG. However, some patients with “polyclonal” ITG may represent monoclonal ITG. Therefore, in patients with polyclonal ITG and rapid disease progression and/or severe disease we suggest empirical treatment as given for monoclonal ITG

**Supportive evidence**

***Diagnosis, evaluation and management of patients with immunotactoid glomerulopathy should be done in (consultation with) expert centers.***

Immunotactoid glomerulopathy (ITG) is an extremely rare glomerular disease. The kidney biopsy incidence is estimated at 1 in 2,500 (0.04%).(188) It is often difficult to establish a diagnosis and select the appropriate treatment.(189) Patients with ITG will benefit from consultation with expert centers where patients are counseled by a team consisting of nephrologists, hematologists, pathologists, and laboratory specialists.

***A diagnosis of immunotactoid glomerulopathy requires a kidney biopsy, showing glomerular patterns of injury, with positive staining of glomerular deposits for IgG, and organized microtubular deposits in EM.***

ITG is a descriptive diagnosis, based on kidney biopsy findings, with EM studies showing the characteristic microtubules, often in parallel arrays, in the mesangium and the capillary wall. Many studies quote 30-90 nm as diameter of the microtubules. ITG must be differentiated from fibrillary glomerulonephritis (FGN), a glomerular disease characterized by the deposition of randomly distributed fibrils, with a diameter < 30 nm. Interestingly, in an older study where the differential diagnosis of FGN and ITG was based on diameter (< > 30 nm) or arrangement of the deposits, a diagnosis of ITG was made more often when using arrangement, indicating that microtubules were often < 30nm in diameter. Recent studies showed that the diameter may range from 14-60 nm, with many patients having values < 20 nm. (188, 190). Thus, the organized microtubular arrangement is the most important diagnostic feature.

On light microscopy, different glomerular patterns of injury can be recognized: endocapillary proliferative pattern, membranoproliferative pattern and a membranous pattern, with considerable overlap (Table 6.2). A purely membranous pattern of injury is observed in less than 10% of patients. The deposits contain IgG, often accompanied by C3 and in 2/3 of cases by C1q. Typically, ITG is considered a subtype of MGRS. Indeed, light chain restriction was observed in 13/14 patients with ITG, and a recent French study even used monotypic deposits as inclusion criterium (190, 191). In contrast, in a US cohort, that included 73 patients, the deposits were monoclonal in 49 (67%), and polyclonal in 24 (33%) patients(188). The latter finding suggests the existence of a polyclonal variant of ITG. In monoclonal ITG there is predominant staining of κ (κ/λ = 2:1), and IgG subclass staining mostly showed dominant staining for the IgG1 and IgG2 subclass (Table 6.2). In most kidney biopsies there is mild-moderate tubular atrophy and interstitial fibrosis, with a tendency of higher severity in polyclonal ITG. The latter might suggest a longer disease duration in patients with polyclonal ITG, or a longer interval till diagnosis. Importantly, in patients with ITG and an underlying CLL interstitial neoplastic infiltrates of CLL were observed in 35% to 60% of patients (188, 190).

***Perform pronase/proteinase immunofluorescence microscopy as salvage technique in ITG when there is inadequate tissue or only weak Ig staining on routine immunofluorescence microscopy***

Pronase/proteinase immunofluorescence microscopy using paraffin tissue is useful in kidney biopsies lacking glomeruli in the frozen tissue sample. Also, it might be an unmasking technique in kidney biopsies which show negative or weak IgG staining, and especially in centers that use transport medium for shipment of kidney biopsies (discussed in Chapter I).

***In patients with immunotactoid glomerulopathy, and no underlying cause, we recommend hematologic evaluation, independent of the clonality of the IgG deposits (monoclonal or polyclonal). The evaluations should include the search for a MIg in serum or urine, and bone marrow aspirate with flow-cytometry, and bone marrow biopsy***

Although rarely described, case reports and small case series have reported an association between HIV and HCV infection and ITG (192, 193). Although in these cases the IgG deposits mostly were polyclonal in IF staining, in some patients monoclonal ITG has been described. Therefore, also in patients with monoclonal ITG an underlying cause should be excluded. Older studies of ITG were very limited and the cohorts included few patients ( < 20). More information is provided by two recent studies that included larger patients cohorts, of 27 respectively 73 patients with ITG (188, 190). The former study only included patients with the monoclonal variant of ITG. In the latter study, there were 49 patients with monoclonal ITG and 24 patients with polyclonal ITG. The clinical characteristics of the patients are given in the Table 6.1. Average age was 60 years, most patients presented with proteinuria (nephrotic syndrome in more than 50% of patients), hematuria, and mild-moderate renal insufficiency. In patients with a monoclonal ITG, a hematologic condition was found in 50-60% of patients (the main cause being CLL). A serological abnormality (either M-protein by serum or urine immunofixation or abnormal FLC ratio was found in at least 50-70% of patients). In the French cohort, ITG was considered the consequence of CLL in 37% of patients, and a MGRS-associated lesion in 52% of patients. In contrast, in the US cohort, a hematologic diagnosis was made in 82% of patients with monoclonal ITG, including MGRS in one quarter. Still, no hematologic diagnosis (serum. Urine, FLC) was made in 18% of patients with monoclonal ITG. We suggest that these cases should also be diagnosed as MGRS, since the absence of abnormalities in serum and urine does not exclude MGRS. Notably, in view of the findings in PGNMID, future studies should use more sensitive technique to proof or exclude monoclonality..

Although a hematologic abnormality is observed less frequently in patients with polyclonal ITG, it is notable that hematologic abnormalities were found in a relative high percentage of patients (26%). Therefore, we suggest that evaluation of patients with polyclonal ITG should be similar to patients with monoclonal ITG. The pathogenesis of polyclonal ITG and its association with an M-protein is further suggested by the finding that a “recurrence” of ITG after kidney transplantation was observed in 3/5 patients with polyclonal ITG, notably in two of these patients the recurrence was characterized by the mere presence of monoclonal deposits (188). One wonders if the “polyclonality” in some patients with ITG is the consequence of concomitant positive staining for IgM and IgA, which is frequently observed (and might be partly explained by the more severe sclerosis and fibrosis, with entrapment of IgM in the glomeruli). In contrast, Carrera et al. described a patient with ITG, who was biopsied three time over a period of 10 years.(194) In the initial biopsy deposits were polyclonal, and the pattern changed to monoclonal during follow-up.

***In patients with Immunotactoid glomerulopathy we recommend conservative treatment for kidney injury as described in chapter I.***

Although there is no evidence specifically for patients with ITG, we advise all patients be treated according the guidelines for CKD.

***We recommend clone targeted treatment in selected patients with immunotactoid glomerulopathy and documented bone marrow abnormalities. We suggest “empirical” chemotherapy in patients with MIg associated immunotactoid glomerulopathy and undetectable bone marrow clone with treatment initiation dependent on comorbidity, age and severity of kidney injury. We suggest “empirical” therapy as used for monoclonal ITG in patients with polyclonal ITG, and progressive kidney disease***.

In the study of Nasr et al. most patients with monoclonal ITG were treated with intensive therapy(188). Both rituximab and various chemotherapeutical agents were used. Remissions developed in 50-80% of patients and ESKD occurred in less than 10% of treated patients. In multivariable analysis treatment with Rituximab and/or chemotherapy was associated with improved survival. Outcome was dismal for patients with polyclonal ITG, with 9/17 patients developing ESKD. However, these data are likely biased since less than half of patients with polyclonal ITG received immunosuppressive therapy.

Javaugue et al. observed better outcome in patients with CLL versus patients with MGRS.(190) This is not unexpected: most patients with CLL received clone targeted therapy, and in univariate analysis clone-targeted therapy and achieving hematologic response were associated with better survival. Many patients with MGRS were not treated with hematologic therapy, and although 2/6 patients developed complete response with solely ACE-inhibition many patients developed ESKD. Of note, the patients who developed spontaneous remission were non-nephrotic and had normal eGFR at diagnosis. Therefore, when making treatment decisions it is important to consider disease severity (level and course of proteinuria, eGFR) and patient characteristics (e.g. in elderly patients with limited life expectancy a wait and see policy may be preferred). In patients deemed good candidate for therapy we suggest to use clone-targeted therapy in patients with monotypic ITG and a defined bone marrow clone. In patients with ITG and an undetectable bone marrow clone (with or without M-protein/abnormal FLC) we suggest to start therapy in patients with severe or progressive kidney disease (nephrotic syndrome; proteinuria and reduced eGFR) who are deemed fit for therapy. Studies have often used Rituximab and chemotherapy with apparent success. In view of the side effect profile, we suggest Rituximab as first treatment option, with addition of second line therapy (chemotherapy with cyclophosphamide, anti-plasma-cell therapy with Bortezomib and Dexamethasone) in non-responders. We suggest a similar approach in patients with polyclonal ITG and progressive disease in view of the abovementioned facts: in up to 25% of patients with polyclonal ITG a hematologic abnormality can be found; the diagnosis of polyclonal ITG is less straightforward (differences in staining intensity between κ and λ light chains are not accounted for, and thus monoclonal ITG may be masked especially in biopsies with positive IgM and IgA staining).

Table 6.1 studies of patients with ITG: clinical characteristics

| Author  Year | Javaugue 2020  Monoclonal ITG* (190) | Nasr 2021  Monoclonal ITG**  (188) | Nasr 2021  Polyclonal ITG (188) |
| --- | --- | --- | --- |
| Country | France | USA | USA |
| Period | 1980-2018 | 1993-2019 | 1993-2019 |
| Patients (N) | 27 | 49 | 24 |
| Age (yr) | 61 (30-79) | 61 (53-73) | 61 (56-69) |
| Gender (M/F) | 18/9 | 21/28 | 13/11 |
| Screat (mg/dl) | 1.5 (0.6-6.5) | 1.5 (1.0-1.9) | 1.9 (1.1-2.9) |
| Salb (g/l) | NA | 3.2 (2.6-3.5) | 2.6 (2.4-3.3) |
| UPCR (g/day) | 6 (1-23) | 6.4 (3.3-8.3) | 7 (3-9.1) |
| Hematuria (%) | 20 (74%) | 39/44 (89%) | 18/22 (82%) |
| Nephrotic Syndrome (%) | 19 (70%) | 26/44 (59%) | 13/23 (57%) |
| Low C3 | 8 (30%) | 10/40 (25%) | 9/21 (43%) |
| M-Protein | 19 (70%) | 23/46 (50%) | 3/19 (16%) |
| FLC abnormal | 3/16 (19%) | 8/29 (28%) | 2/13 (15%) |
| Hematologic condition | 13 (48%) | 40^&&^ (82%) | 5/19 (26%)^##^ |
| MGRS | 14$ (52%) | 10/49 (20%)# | 1. (0%) |

*values are given as median (range) or **median (IQR). &6 patients had bone marrow flow cytometry, which was abnormal in 5; && MGRS was listed as hematologic condition in this study. ## MGUS was listed as hematologic condition in this study. # It is unclear why patient with monoclonal ITG , without abnormal immunofixation or abnormal FLC would not fulfil the MGRS definition.

Table 6.2 Studies of patients with ITG: kidney biopsy characteristics

| Author  Year | Javaugue  2020  Monoclonal ITG | Nasr  2021  Monoclonal ITG | Nasr  2021  Polyclonal ITG |
| --- | --- | --- | --- |
| Country | France | USA | USA |
| Period | 1980-2018 | 1993-2019 | 1993-2019 |
| Patients (N) | 27 | 49 | 24 |
| Pattern of injury* |  |  |  |
| Endocapillary |  | 15 (31%) | 10 (42%) |
| MPGN | 11 (41%) | 15 (31%) | 6 (25%) |
| Membranous | 16 (59%) | 16 (33%) | 5 (21%) |
| Mesangial prol. |  | 3 (6%) | 2 (8%) |
| Biopsy with crescents | 2 (8%) | 10 (20%) | 5 (25%) |
| Immunofluorescence |  | ** |  |
| IgG1 | 11/18 (61%) | 67% |  |
| IgG2 | 5/18 (28%) | 27% |  |
| IgG3 | 2/18 (11%) | 0% |  |
| IgG4 | 0% | 7% |  |
| Kappa | 17 (63%) | 31 (67%) |  |
| Lambda | 10 (37%) | 18 (37%) |  |

*often mixed patterns, predominant pattern is indicated. ** IgG subclass staining was done in 43 patients and restriction was observed in 29 patients with monoclonal ITG and in one patient with polyclonal ITG. Polyclonal IgG was observed in 11 patients, of which 8 with light chain restriction and 3 without light chain restriction In 2 patients IgG subclass staining was negative.

**T**able 6.3 Studies of patients with ITG: treatment and outcome

| Author  Year | Javaugue  2020  Monoclonal ITG | Nasr  2021  Monoclonal ITG | Nasr  2021  Polyclonal ITG |
| --- | --- | --- | --- |
| Country | France | USA | USA |
| Period | 1980-2018 | 1993-2019 | 1993-2019 |
| Patients (N) | 27 | 39* | 17* |
| **Treatment** |  |  |  |
|  |  |  |  |
| Rituximab based | 9/27 (33%( | 15/35 (43%) | 7/13 (54%) |
| Alkylating agent | 8/27 (29%) | 7/35 (20%)** | 7/13 (54%) |
| Other | 1.27 (4%) | 12/35 (34%) | 7/13 (54%) |
| Emperical | 3/27 (11%) |  |  |
| RASblockers +/- steroids | 6/27 (22%) | 7/35 (20%) | 7/13 (54%) |
| **Outcome** |  |  |  |
| Complete renal response | 12 (44%) | 15/38 (39%) | 2/17 (12%) |
| Partial renal response | 8 (30%) | 4/38 (11%) | 2/17 (12%) |
| No-response | 7 (26%) | 19/38 (50%) | 13/17 (76%) |
| ESKD | 7 (26%) | 4 (10%) | 9 (53%) |
|  |  |  |  |

*in many patients no information; treatment known in 35 resp 13 patients; ** these patients also received rituximab, and are included in the rituximab group also.

**Chapter 7: Fibrillary glomerulonephritis with monoclonal immunoglobulin deposits.**

**Introduction**

Fibrillary glomerulonephritis is a rare disease, characterized by the presence of Congo red-negative, immunoglobulin derived, fibrillary deposits in the mesangium and/or glomerular capillary wall. In the majority of cases the fibrillary deposits consist of polyclonal IgG, and demonstrate positive staining for DNAJB9. Only in very rare cases the deposits are composed of a monoclonal immunoglobulin.

**Summary statements**

***Diagnosis, evaluation and management of patients with fibrillary glomerulonephritis should be done in (consultation with) expert centers.***

***A diagnosis of fibrillary glomerulonephritis requires a kidney biopsy, showing glomerular patterns of injury, with positive staining of glomerular deposits for IgG, and organized fibrillary deposits in EM.***

***In patients with a suspected diagnosis of fibrillary glomerulonephritis, the biopsy should be stained for DNAJB9. Patients with DNAJB9 positive, polyclonal fibrillary glomerulonephritis are not included within the spectrum of MGRS.***

***In patients with suspected monoclonal fibrillary glomerulonephritis evaluation of the kidney biopsy should include IgG subclass staining and DNAJB9 staining. Additional IF/IHC on pronase/proteinase digested tissue should be considered.***

***Patients with confirmed monoclonal fibrillary glomerulonephritis should be evaluated and treated as patients with immunotactoid glomerulopathy.***

**Rationale**

***Diagnosis, evaluation and management of patients with fibrillary glomerulonephritis should be done in (consultation with) expert centers.***

Fibrillary glomerulonephritis (FGN) is a rare form of glomerulonephritis. It not only requires expertise (including EM facilities) to diagnose FGN, but it is even more difficult to make a definite diagnosis of monoclonal immunoglobulin associated FGN. Patients with FGN (be it polyclonal or monoclonal) will benefit from consultation with expert centers where patients are counseled by a team consisting of nephrologists, hematologists, pathologists, and laboratory specialists.

***A diagnosis of fibrillary glomerulonephritis requires a kidney biopsy, showing glomerular patterns of injury, with positive staining of glomerular deposits for IgG, and organized fibrillary deposits in EM.***

Patients with FGN present with variable degree of kidney insufficiency and proteinuria, often accompanied by hematuria. The diagnosis requires a kidney biopsy, and EM evaluation is essential to demonstrate that the deposits contain the characteristic organized, non-branching, randomly oriented fibrils with a mean diameter of 12-22 nm. On light microscopy, different patterns of glomerular involvement can be identified. By immunofluorescence (IF), the somewhat ill-defined, ‘smudged’ deposits stain most intensely for IgG, and are typically positive for both kappa and lambda light chains. In very rare cases of FGN, IF staining is characterized by light chain restriction, and the presence of a single IgG subclass, which defines monoclonal FGN. FGN must be differentiated from amyloidosis. Typically, amyloid deposits are congo-red positive whereas the deposits in FGN are congo-red negative. However, some FGN cases stain positive in congo-red staining (Congophilic FGN). The discovery of positive DNAJB9 staining in FGN has allowed accurate differentiation. In EM amyloid fibrils are on average smaller in diameter, although there is considerable overlap.

***In patients with a suspected diagnosis of fibrillary glomerulonephritis, the kidney biopsy should be stained for DNAJB9. Patients with DNAJB9 positive, polyclonal fibrillary glomerulonephritis are not included within the spectrum of MGRS***

It has been shown that DNAJB9 is a major component of the fibrils in FGN. This DNAJB9 staining is highly specific, and positive immunohistochemical staining for DNAJB9 enables an accurate diagnosis of FGN in the absence of EM evaluation. Most cases of DNAJB9 positive FGN show polyclonal IgG staining. Polyclonal FGN is not considered a subtype of MGRS.

***In patients with suspected monoclonal fibrillary glomerulonephritis evaluation of the kidney biopsy should include IgG subclass staining and DNAJB9 staining. Additional IF/IHC on pronase/proteinase digested tissue should be considered.***

Very few patients will present with monoclonal FGN. Therefore, all efforts must be made to confirm the diagnosis. In some centers the observed light chain restriction is an artefact, due to masking, and additional studies using pronase/proteinase digestion will reveal that the deposits are polyclonal. IgG subclass staining also allows to differentiate between polyclonal and monoclonal FGN. Although DNAJB9 staining is often negative in monoclonal FGN, positive staining for DNAJB9 does not exclude monoclonal FGN. Thus, a definite diagnosis of monoclonal FGN requires expert evaluation of the kidney biopsy.

***Patients with confirmed monoclonal FGN should be evaluated and treated as patients with immunotactoid glomerulopathy.***

There is no evidence to support treatment guidance for patients with monoclonal FGN. We suggest that patients with a definite diagnosis of monoclonal FGN should be managed according patients with ITG (discussed in chapter VI)

**Supportive evidence**

***Diagnosis, evaluation and management of patients with fibrillary glomerulonephritis should be done in (consultation with) expert centers.***

A diagnosis of fibrillary glomerulonephritis (FGN) is made in 0.5-1.0% of native kidney biopsies (195). Typically, IF staining for IgG is polyclonal. In only 5-10% of cases there is evidence of light chain restriction (“monoclonal” or “monotypic FGN”). It not only requires expertise (including EM facilities) to diagnose FGN, but it is even more difficult to make a definite diagnosis of monoclonal-immunoglobulin-associated FGN. Patients with FGN (be it polyclonal or monoclonal) will benefit from consultation with expert centers where patients are counseled by a team consisting of nephrologists, hematologists, pathologists, and laboratory specialists.

***A diagnosis of fibrillary glomerulonephritis requires a kidney biopsy, showing glomerular patterns of injury, with positive staining of glomerular deposits for IgG, and organized fibrillary deposits in EM.***

Patients with FGN present with variable degree of kidney insufficiency and proteinuria, often accompanied by hematuria. The diagnosis requires a kidney biopsy, and EM evaluation is essential to demonstrate that the deposits contain the characteristic organized, non-branching, randomly oriented fibrils with a mean diameter of 12 - 22 nm. (196) For comparison the fibrils in AL amyloidosis typically are 8-15 nm. On light microscopy, different patterns of glomerular involvement can be identified, most commonly mesangial and endocapillary proliferative glomerulonephritis, MPGN, and more rarely crescentic glomerulonephritis, or pure membranous nephropathy.(196) In immunofluorescence (IF), the somewhat ill-defined, ‘smudged’ deposits stain most intensely for IgG, are typically positive for both κ and λ light chains, and usually also for C3. Staining for C1q, IgM and/or IgA may be positive. In rare cases of FGN, IF shows light chain restriction, We suggest that the suspicion of monoclonal FGN be confirmed by IgG subclass staining, with monoclonal FGN defined by the presence of a single IgG subclass. FGN must be differentiated from amyloidosis, which is also characterized by the presence of fibrils, usually of slightly lower diameter. Typically, amyloid deposits are congo-red positive whereas the deposits in FGN are congo-red negative. However, in one study 4% of all FGN cases stained positive in congo-red staining (“Congophilic FGN”) (46). The discovery of positive DNAJB9 staining in FGN has allowed accurate differentiation.

***In patients with a suspected diagnosis of fibrillary glomerulonephritis, the kidney biopsy should be stained for DNAJB9. Patients with DNAJB9 positive, polyclonal fibrillary glomerulonephritis are not included within the spectrum of MGRS.***

It has been shown that DNAJB9 is a major component of the fibrils in FGN. DNAJB9 staining is very specific, and therefore positive immunohistochemical staining for DNAJB9 allows an accurate diagnosis of FGN in the absence of EM evaluation(45). The latter study reported a sensitivity of 98% and specificity of 99% for the diagnosis of FGN. Most cases of DNAJB9 positive FGN show polyclonal IgG staining. These cases of polyclonal FGN are not part of the MGRS spectrum, and this entity is nor further discussed here.

***In patients with suspected monoclonal fibrillary glomerulonephritis evaluation of the kidney biopsy should include IgG subclass staining and DNAJB9 staining. Additional IF/IHC on pronase/proteinase digested tissue should be considered.***

The existence of monoclonal FGN is debated. Da et al. suggested that a diagnosis of monoclonal FGN is sometimes based on light chain restriction only, in the absence of confirmation by IgG subclass staining. (197). They explicitly mention the limited sensitivity of light chain staining (favoring one light chain). Based on literature data, monoclonal FGN as entity cannot be excluded, although it is very rare. Older studies reported light chain restriction in 3-11% of patients (191, 195, 198-200). It is likely that not all of these cases really represented monotypical FGN, since these studies mostly lacked confirmation by IgG subclass staining. This is nicely illustrated by a French study, which included 27 patients with FGN (200). In two patients light chain restriction was noted. It is unlikely that these patients had monoclonal FGN (and thus MGRS): in both cases, the kidney biopsies showed polyclonal Ig staining; in one patient there was positive staining for IgG and IgA, and in the other patient IgG subclass staining showed both IgG1 and IgG4. Moreover, although one of these patients was diagnosed with a serum M-protein, the culprit M-protein was IgGκ, whereas the kidney biopsy showed lambda light chain restriction. Of note, the mere presence in the serum or urine of a monoclonal M-protein should not be used to define monoclonal FGN, since most patients with FGN are of older age, and MGUS can be expected. On the other hand, the absence of light chain restriction may not necessarily provide 100% certainty that there is no association with a monoclonal immunoglobulin (see below).

Light chain restriction detected by routine IF staining of frozen tissue samples with antibodies against κ and λ chains may signal monoclonal FGN. However, it is important to be aware of “masked” deposits. This may be particular relevant in centers that use special storage medium for transport of kidney biopsies. Said et al. reported a 9% prevalence of apparent light chain restriction in FGN (14/151 biopsies in the period 2017-2019) (201). They retrieved additional biopsies, collected after 2011. In total they collected 35 kidney biopsies of patients with DNAJB9 positive FGN, with apparent light chain restriction (21 λ, 14 κ). All biopsies were assessed using IF on formalin-fixed paraffin embedded tissue after pronase digestion (IF-P), which allowed unmasking of “hidden” light chains. In 15/35 biopsies IF-P revealed a hidden light chain (with unmasking of κ in 14, and unmasking of λ in 1). In the remaining 20 biopsies light chain restriction was confirmed, in 12/15 available biopsies monoclonality was confirmed by IgG subclass staining.

Kidney biopsy tissue should be stained for DNAJB9 if FGN is suspected. DNAJB9 negative FGN is rare if at all. In DNAJB9 negative FGN a diagnosis of monoclonal FGN must be considered. In one study, which included 84 biopsies with FGN, only 2 biopsies stained negative for DNAJB9 (45). Both biopsies showed staining for IgG only (and were negative for κ or λ light chains), and both patients had clinical evidence of a monoclonal gammopathy. These patients are likely examples of Heavy Chain FGN (202).

The value of detailed evaluation of a kidney biopsy in patients with suspected monotypical FGN is illustrated by Kudose *et al. (203).* These investigators retrieved 29 biopsies of patients with FGN and light chain restriction on routine IF-F. The biopsies were evaluated in more detail, using congo-red staining, DNAJB9 immunostaining, IgG subclass staining and IF-P. This resulted in a major reclassification of the biopsies: they diagnosed polytypic DNAJB9-positive FGN in 14 patients, monotypic DNAJB9-positive FGN in 7 patients, polytypic DNAJB9-negative FGN in 2 patients and monotypic DNAJB9 negative FGN in 6 patients. Thus, in this study only 13/29 cases with an initial diagnosis of monotypic FGN were confirmed. The study also confirmed that DNAJB9 negative FGN is often associated with monoclonal Ig deposition (in 6/8 patients). In 5 of these 6 patients there was evidence of an underlying hematologic disease (chronic lymphocytic leukemia in 4 and B cell lymphoma in 1). Of note, kidney biopsies of patients with monotypic DNAJB9-negative fibrillar IgG deposits often had features of immunotactoid GN including focally parallel fibril alignment, but absence of definitive hollow cores. Thus, patients with DNAJB9 negative monotypic FGN should be considered part of the MGRS spectrum, with close resemblance to ITG.

Only few patients with monotypic DNAJB9-positive FGN have hematologic abnormalities. Two studies reported positive M-protein finding in only 1 of 6 patients (45, 203). It remains debatable whether these patients with DNAJB9 positive monoclonal FGN represent a subtype of MGRS. Still, the absence of an M-protein in serum or urine, and a normal FLC ratio is not sufficient to exclude monoclonal-immunoglobulin-associated kidney disease as discussed for PGNMID in chapter V.

On the other hand, it can be questioned if the absence of light chain restriction is sufficient to rule out monoclonal immunoglobulin-associated FGN. In the study of Said et al. 2/13 patients with polyclonal FGN had a M-protein detected by SPEP (201). Of interest, in one patient FLC ratio was increased (κ/λ ratio 20), and bone marrow biopsy revealed a smouldering multiple myeloma. It is also important to realize that in some patients with “absent” light chain restriction there is often dominant staining for one light chain (e.g kappa 1+ and lambda 2-3+). In many of these cases, IF staining of the biopsy discloses IgA and IgM staining, in addition to the IgG. IgM deposition might be non-specific, however may be responsible for the added low intensity light chain staining.

***Patients with confirmed monoclonal FGN should be evaluated and treated as patients with immunotactoid glomerulopathy.***

Monotypic FGN is a very rare entity, and very strict criteria are needed to establish the diagnosis. There are no large cohorts of patients with monotypic FGN, therefore it is impossible to provide any evidence based treatment advice. We suggest that patients with monotypic FGN should be evaluated and treated as described for patients with immunotactoid glomerulopathy (chapter VI). In the absence of hematologic disease and/or hematologic abnormalities it seems best to restrict hematologic therapy to patients with proof of severe or progressive disease.

**Chapter 8: Light-chain proximal tubulopathy and light chain crystalline podocytopathy**

**Introduction**

Light-chain proximal tubulopathy (LCPT) is defined by the presence of cytoplasmic light chain (most often crystalline) inclusions in the proximal tubular cells, causing proximal tubular toxicity, with or without interstitial inflammation, and associated with complete or incomplete Fanconi syndrome.

Light chain crystalline podocytopathy (LCCP) is defined by the presence of crystalline deposits in the podocyte, causing podocyte injury.

**Summary statements**

Light chain proximal tubulopathy (LCPT)

***Diagnosis, evaluation and management of patients with (suspected) LCPT should be done in (consultation with) expert centers.***

***A diagnosis of LCPT must be suspected in patients with a known monoclonal immunoglobulin and evidence of proximal tubular dysfunction.***

***A clinical diagnosis of LCPT can be made with certainty in patients with complete Fanconi syndrome and abnormal serum free light chains. In these patients, a kidney biopsy may be omitted.***

***A histological diagnosis of LCPT requires a kidney biopsy, showing light chain restriction in the proximal tubular cells. The presence of crystals and/or evidence of complete or incomplete Fanconi syndrome support a diagnosis of LCPT. LCPT as disease entity in the absence of crystals in the proximal tubules and features of Fanconi syndrome is debated.***

***We recommend the use of immunofluorescence on pronase/proteinase -digested paraffin embedded tissue in all patients with suspected LPCT and negative routine immunofluorescence.***

***We recommend ultrastructural EM evaluation, to detect crystalline inclusions or intracytoplamic (lysosomal) fibrillary or amorphous deposits, in patients with suspected LCPT.***

***Patients with suspected LCPT should be evaluated for the presence of proximal tubular dysfunction, characterized by aminoaciduria, normoglycemic glycosuria, renal proximal tubular acidosis , hypo-phosphatemia and hypo-uricemia with increased fractional excretion of phosphate or uric acid.***

***We recommend that patients with LCPT should be evaluated for the presence of metabolic complications of the tubulopathy, which are mainly related to the severity of phosphate loss, and manifest as osteoporosis, non-traumatic fractures, and sometimes rickets.***

***Extrarenal light-chain deposition associated organ involvement is not seen in LCPT. Therefore, there is no need for untargeted evaluation of patients with LCPT to detect extrarenal manifestations.***

***In patients with LCPT, we recommend detailed hematologic evaluation including serum and urine electrophoresis and immunofixation, serum FLC, urinary light chain excretion, followed in patients with positive results by bone marrow aspirate and flow-cytometry, and bone marrow biopsy .***

***In patients with LCPT, we recommend maximal conservative treatment for kidney injury as described in chapter I. We recommend treatment of the metabolic complications guided by the observed abnormalities (vitamin D supplementation, correction of acidosis or hypophosphatemia).***

***The decision to start hematologic treatment should be based on individual patient characteristics. We recommend clone-targeted treatment in patients with progressive kidney failure, or patients with severe and untreatable metabolic complications. Treatment should be guided by changes in FLC.***

Light chain crystalline podocytopathy (LCCP)

***A diagnosis of LCCP requires a kidney biopsy, showing crystals in the podocyte that are composed of a monoclonal light chain. Since crystals are not always visible in LM, and immunofluorescence on frozen tissue is often falsely negative a diagnosis of LCCP requires detailed examination of the kidney biopsy with EM and immunofluorescence on paraffin after pronase digestion.***

***Patients with LCCP should be evaluated for the presence of LCPT.***

***Diagnosis, evaluation, and treatment of patients with LCCP should be done as described for patients with LCPT***

**Rationale**

Light chain proximal tubulopathy (LCPT)

***Diagnosis, evaluation and management of patients with (suspected) LCPT should be done in (consultation with) expert centers.***

LCPT is a rare disease. It requires expertise to diagnose LCPT, and in the absence of crystals and/or Fanconi syndrome it is not easy to attribute kidney injury to the culprit light chain. Patients will benefit from consultation with expert centers.

***A diagnosis of LCPT must be suspected in patients with a known monoclonal immunoglobulin and evidence of proximal tubular dysfunction.***

In adults, LCPT is the major cause of Fanconi syndrome. In most patients, the Fanconi syndrome is “incomplete”, thus some of the typical elements of Fanconi syndrome (i.e. aminoaciduria, normoglycemic glycosuria, proximal RTA, hypophosphatemia and hypo-uricemia with increased fractional excretion of phosphate or uric acid) are lacking. Adult patients who present with features of Fanconi syndrome, should be evaluated for the presence of increased light chains in serum and urine. On the other hand, patients with “MGUS” and increased light chains in urine should be evaluated for proximal tubular dysfunction.

***A clinical diagnosis of LCPT can be made with certainty in patients with complete Fanconi syndrome and increased serum FLC. In these patients, a kidney biopsy may be omitted.***

The presence of complete Fanconi syndrome in patients with a monoclonal immunoglobulin and abnormal FLC in serum and urine is considered sufficient to diagnose LCPT. A kidney biopsy is not necessary for a diagnosis of LCPT. Notably, a kidney biopsy should be done in patients who present with features that are not typical for LCPT, such as severe proteinuria or rapid deterioration of kidney function. A kidney biopsy may also provide information on the extent of chronic kidney injury, and be used to guide treatment decisions.

***A histological diagnosis of LCPT requires a kidney biopsy, showing light chain restriction in the proximal tubular cells. The presence of crystals and/or evidence of complete or incomplete Fanconi syndrome support a diagnosis of LCPT. LCPT as disease entity in the absence of crystals in the proximal tubules and the absence of features of Fanconi syndrome is debated.***

In patients with incomplete Fanconi syndrome a kidney biopsy is mandatory to diagnose LCPT. The presence of crystals (light chain positive) is almost pathognomonic for a diagnosis of LCPT. However, crystalline inclusions may be absent, and proximal tubular injury may be present and associated with fibrillary or amorphous deposits or irregular large lysosomes. Notably, proximal tubule light chain restriction may be observed in patients presenting with kidney insufficiency in the absence of Fanconi syndrome or crystals. It is debated if this represents LCPT. These patients have been diagnosed with “light chain nephropathy”, although the role of the light chain in causing kidney injury is not always proven, and the observed light chain restriction may simply reflect normal physiology in patients with an abnormal FLC ratio.

***We recommend the use of immunofluorescence on pronase-digested paraffin embedded tissue in all patients with suspected LPCT and negative routine IF.***

Since routine IF staining is often falsely negative, especially in crystalline LCPT, immunofluorescence on pronase/proteinase pretreated paraffin embedded tissue should be done in all patients with suspected LPCT and negative routine IF.

***We recommend ultrastructural EM evaluation, to detect crystalline inclusions, or intracytoplamic (lysosomal) fibrillary or amorphous deposits, in patients with suspected LCPT.***

In some patients with LCPT light microscopy and immunofluorescence may suffice to diagnose LCPT, the kidney biopsy typically showing crystalline inclusions in the proximal tubular cells, which are positive for light chain staining (usually κ light chain) in IF. Still, in light microscopy crystals are often not easily detected. In addition, non-crystalline proximal tubulopathy does occur, and IF on frozen tissue is often negative. In such cases EM ultrastructural evaluation is informative, by showing either cytoplasmic crystalline inclusions, fibrillary material, or amorphous dense granular deposits in cytoplasm or phagolysosomes.

***Patients with suspected LCPT should be evaluated for the presence of proximal tubular dysfunction, characterized by aminoaciduria, normoglycemic glycosuria, proximal renal tubular acidosis , hypo-phosphatemia and hypo-uricemia with increased fractional excretion of phosphate or uric acid.***

A diagnosis of LCPT is supported by the presence of features of proximal tubular injury, which includes any of the following: renal glucosuria, renal hypophosphatemia, renal hypo-uricemia, proximal renal tubular acidosis, amino-aciduria, accompanied by increased excretion of low molecular weight proteins. A diagnosis of LCPT should be reconsidered in patients with suspected LCPT in the absence of any evidence of Fanconi syndrome.

***We recommend that patients with LCPT should be evaluated for the presence of metabolic complications of the tubulopathy, which are mainly related to the severity of phosphate loss, and manifest as osteoporosis, non-traumatic fractures, and sometimes rickets.***

Most features of renal proximal tubular injury do not cause symptoms (aminoaciduria, glucosuria, hypo-uricemia). Phosphate loss may lead to bone disease. Proximal renal tubular acidosis may contribute to hypokalemia. Dyspnea as a result of compensatory hyperventilation is rare, since in proximal RTA serum bicarbonate levels usually are maintained > 15 mmol/l, the threshold of proximal tubular bicarbonate reabsorption.

***Extra-renal light-chain deposition associated organ involvement is not seen in LCPT. Therefore, there is no need for untargeted evaluation of patients with LCPT to detect extrarenal manifestations.***

LCPT is a renal limited disease. Extrarenal manifestations due to deposits of Mig in other organs do not occur.

***In patients with LCPT, we recommend detailed hematologic evaluation including serum and urine electrophoresis, and immunofixation, and serum and urine FLC, followed by bone marrow aspirate and flow-cytometry, and bone marrow biopsy.***

LCPT is the consequence of increased light chain production, and appropriate hematologic evaluation is mandatory. Patients with a documented M-protein or abnormal FLC should be further evaluated with bone marrow biopsy, and bone marrow aspirate with flowcytometry. If hematologic evaluation of serum and urine is negative, the diagnosis of LCPT should be reconsidered.

***In patients with LCPT, we recommend maximal conservative treatment as described for CKD. We recommend treatment of the metabolic complications guided by the observed abnormalities (vitamin D supplementation, correction of acidosis or hypophosphatemia).***

Most patients with LCPT present with chronic kidney disease, and although not studied in this population, conservative treatment guided by the principles of CKD management is advised. Bone abnormalities are often observed, related to phosphate loss, vitamin D deficiency, and acidosis. These abnormalities should be evaluated and treated.

***The decision to start hematologic treatment should be based on individual patient characteristics. We recommend clone-targeted treatment in patients with progressive kidney failure, or patients with severe and untreatable metabolic complications. Treatment initiation may also be guided by the extent of tubulo-interstitial injury. We suggest that changes in FLC are useful to monitor an dguide treatment..***

Many patients with LCPT will present with an underlying hematologic malignancy, and be treated accordingly. Patients with LCPT as subtype of MGRS often show slow progression rate, and a wait and see policy may be preferred. Patients with metabolic complications, or patients with (high risk of) kidney function deterioration may benefit from hematologic treatment, targeting the abnormal clone. The extent of interstitial inflammation/fibrosis on a kidney biopsy may guide the decision to start therapy. Monitoring of FLC allows to predict treatment response.

Light chain crystalline podocytopathy

**A diagnosis of LCCP requires a kidney biopsy, showing crystals in the podocyte that are composed of a monoclonal light chain. Since crystals are not always visible in LM, and immunofluorescence on frozen tissue is often falsely negative a diagnosis of LCCP requires detailed examination of the kidney biopsy with EM and immunofluorescence on paraffin embedded tissue after pronase digestion**.

LCCP is only recently proposed as disease entity, characterized by the presence of light chain restricted, crystalline deposits in the podocyte. Identification of the crystalline structure often requires EM evaluation. Routine IF on frozen tissue is often falsely negative, and identification of the culprit light chain requires IF or immunohistochemistry on paraffin-embedded tissue after pronase/proteinase digestion.

***Patients with LCCP should be evaluated for the presence of LCPT.***

Many patients with LCCP in parallel show histological features of LCPT. Patients with histological features of LCPT should be evaluated as described above.

***Diagnosis, evaluation, and treatment of patients with LCCP should be done as described for patients with LCPT*** .

LCCP is an ultrarare disease, it is advised to consult expert centers to ascertain diagnosis and aid in management. In patients with LCCP an underlying hematologic disorder (MGRS or MM) is usually detected. Most patients with LCCP present with moderate – severe proteinuria, and will likely benefit from treatment as proposed for patients with proteinuric CKD. The limited available data suggest that in LCCP renal response is observed in patients with hematologic response to therapy. However, in view of the paucity of data, patient characteristics such as age, severity of kidney injury and rate of progression should be balanced against the benefits and risks of aggressive therapy

**Supportive evidence**

Light chain proximal tubulopathy

***Diagnosis, evaluation and management of patients with (suspected) LCPT should be done in (consultation with) expert centers.***

LCPT is a rare disease. LCPT was diagnosed in 4-5% of patients with monoclonal immunoglobulin associated kidney disease (204) (205) and in 0.13-0.18% of all kidney biopsies (204, 206). The diagnosis requires expertise. The mere finding of monoclonal light chain restriction in proximal tubules in the absence of crystalline inclusions and Fanconi syndrome should lead to reconsider the diagnosis. Patients with suspected LCPT will benefit from consultation with expert centers.

***A diagnosis of LCPT must be suspected in patients with a known monoclonal immunoglobulin and evidence of proximal tubular dysfunction.***

Although LCPT are rare diseases, we suggest that evaluation of proximal tubular dysfunction should be considered in patients with a known M-protein, and certainly if there is evidence of abnormal FLC and/or decreased eGFR. The evaluation can be done with simple and cheap tests, e.g. serum phosphorus, uric acid and bicarbonate, and screening of the urine for pH, and glucosuria. In patients with some features of proximal tubulopathy (incomplete Fanconi syndrome) a kidney biopsy is mandatory to diagnose LCPT (205, 207).

***A clinical diagnosis of LCPT can be made with certainty in patients with complete Fanconi syndrome and abnormal serum FLC. In these patients, a kidney biopsy may be omitted.***

In adult patients, Fanconi syndrome is mostly an acquired disorder, with tubular dysfunction caused by drugs, a systemic disease, or monoclonal immunoglobulin-induced cell injury. Of these, light chain induced proximal tubular injury is the major cause of Fanconi syndrome. In case of complete Fanconi syndrome (i.e. aminoaciduria, normoglycemic glycosuria, proximal RTA, hypo-phosphatemia, and hypo-uricemia with increased fractional excretion of phosphate or uric acid), associated with abnormal FLC, many experts acknowledge that a kidney biopsy is not necessary for the diagnosis of LCPT (207). In many studies of patients with “LCPT”, histological confirmation of the diagnosis is thus lacking. For example, a kidney biopsy was available in 39/49 (80%) patients in a French study and in only 50% of patients in two Chinese studies (207-209). Still, a kidney biopsy may be needed in such patients, especially when presenting with features that are not explained by LCPT, i.e. nephrotic syndrome or AKI. A kidney biopsy may also be helpful to guide treatment decisions. Severe chronic interstitial fibrosis and tubular atrophy in a patient with low eGFR (< 20-30 ml/min/1.73m^2^) may signal futility of treatment. On the other hand, the presence of interstitial inflammation, and interstitial fibrosis in a patient with preserved eGFR may foster start of therapy.

***A histological diagnosis of LCPT requires a kidney biopsy, showing light chain restriction in the proximal tubular cells. The presence of crystals and/or evidence of complete or incomplete Fanconi syndrome support a diagnosis of LCPT. LCPT as disease entity in the absence of crystals in the proximal tubules and the absence of features of Fanconi syndrome is debated.***

***We recommend the use of immunofluorescence on pronase/proteinase-pretreated paraffin embedded tissue in all patients with suspected LPCT and negative routine IF.***

***We recommend ultrastructural EM evaluation, to detect crystalline inclusions, or lysosomal/cytoplasmic fibrillary or amorphous deposits in patients with suspected LCPT.***

LCPT is attributed to the deposition of a monoclonal light chain in the proximal tubular cell causing tubular cell injury, with ensuing tubular cell dysfunction (resulting in Fanconi syndrome) and peritubular inflammation (resulting in IFTA and decreased eGFR). The classical features of LCPT include: 1. Light chain restricted intracytoplasmic staining of proximal tubular cells (with positive staining for κ light chain in > 90%); 2. The presence of crystalline structures in the proximal tubular cells (structured deposits reflecting the monoclonality); 3. Evidence of Fanconi syndrome, often incomplete.

Although this may seem straightforward, there are many caveats.

1. Routine IF staining on frozen tissue (IF-F) is often false-negative (especially in crystalline LCPT). Therefore, IF or IHC on pronase/protease digested paraffin embedded tissue should be done in all patients with suspected LPCT and negative IF-F. In a US study of patients with LCPT, with crystalline deposits in almost 90% of cases, routine IF-F was positive in only 35% of patients with LCPT, whereas 97% of cases showed proximal tubular light chain restriction using IF after pronase digestion (205). Similar data were reported from China (210). In this study of 47 patients with LCPT, only 34% of cases were detected by IF-F, and 95% by IF-P.

2. Routine light-microscopy may miss crystals. In light microscopy, no crystals were observed in 5/38 patients with crystalline LCPT, a false negative rate of 13% (205). Another study included 24 patients with crystalline LCPT, and in only 17 patients the crystals were detected by LM, a false-negative rate of 29% (207). The presence of crystals was proven by EM in all cases. Thus, EM is indispensable to disclose crystals.

3. Importantly, and rarely discussed in the literature, interpretation of laboratory test results for the evaluation of Fanconi syndrome is difficult in patients with kidney dysfunction: patients with reduced eGFR might have low-normal serum phosphorus and serum uric acid despite increased fractional excretion. Also, in patients with low eGFR acidosis is expected and might not easily be recognized as sign of proximal RTA. This is illustrated in the report of Messiaen et al.(211). In this study of 11 patients, there were 4 patients with normal serum phosphorus levels (0.92-1.13 mmol/l) despite evident tubular phosphate loss as reflected by decreases TmP/GFR (0.32 – 0.50).

Table 8.1 illustrates the characteristics of patients with LCPT from three larger cohort studies from Europe, the US, and China(210) (205, 207). The study of Vignon et al. included 49 patients. In 10 patients a kidney biopsy was not available, since the authors favored a clinical diagnosis of LCPT in patients with a MIg and complete Fanconi syndrome. In biopsied patients in all studies LCPT was defined by cytoplasmic light chain restricted staining of the proximal tubular cells. In 62%-94% of patients, the crystalline variant of LCPT was observed. The lower prevalence of 62% might be partly explained by omitting a kidney biopsy in patients with complete Fanconi syndrome. Light chain κ is the dominant light chain in LCPT, and was present in 44/46, 37/39, and 41/47 patients respectively (mean prevalence 93%). The studies differ with respect to the presence of Fanconi syndrome. Vignon et al. required the presence of at least two abnormalities of proximal tubular dysfunction for inclusion in the study, thus features of Fanconi syndrome were present in all patients.(207) In contrast, features of Fanconi syndrome were present in only 17 of 45 evaluable patients reported by Stokes et al.(205) However, the authors acknowledge that in the 28 “negative” patients no detailed test results were available, thus it is unknown if all features of Fanconi syndrome were tested. Moreover, 83% of patients had renal insufficiency, which affects the interpretation of features such as hypophosphatemia, hypo-uricemia, and acidosis. Fanconi syndrome was present in 68% of patients in the study from China. Although this is not specifically mentioned, this likely represents complete Fanconi syndrome since features of Fanconi syndrome were present in many more patients (see table 8.1), and aminoaciduria and acidosis were not mentioned. Therefore, the studies do not allow to conclude that LCPT can present without features of Fanconi syndrome.

Still, the prevalence of Fanconi syndrome features in patients with LCPT is debated. In a recent review, Kousios et al. summarized 6 large case series, totaling 186 patients(206) (211, 212) (205) (207-209). Most patients had crystalline LCPT, and there was predominant κ light chain restriction (90%). Features of Fanconi syndrome (complete or incomplete) were observed in all but one studies, although this data is somewhat biased since the presence of Fanconi syndrome was an inclusion criterium in most studies. The only study that included many patients with no reported Fanconi syndrome, is the study of Stokes et al., of which the limitations have been discussed.

Therefore, we suggest that LCPT can be diagnosed in patients who present with mild-moderate proteinuria or decreased eGFR, who have evidence of crystalline deposits in the proximal tubular cells, with light chain restriction (mostly κ) in IF, and features of Fanconi syndrome. Of note, there also must be evidence of a M-protein, an abnormal FLC ratio, and increased urine light chain proteins (see below). Very few patients (if at all) with crystalline LCPT will present without evidence of Fanconi syndrome. Said et al. described a patient with a kidney biopsy showing crystals in LM, confirmed by EM, and κ light chain restriction in IF (213). This patient was known with an IgGκ M-protein, and at the time of kidney biopsy a diagnosis of SMM was made (20% κ restricted plasma cells, IgGκ M protein in serum, κ BJP in urine, not quantified). Apparently, there were no features of Fanconi syndrome. Although this report indeed suggests that crystalline LCPT can present without Fanconi syndrome, this case is peculiar since at the time of kidney biopsy, the patient had stable CKD, with serum creatinine at 1.6mg/dl unchanged over a period of 3 years. In this period there also was stable low grade proteinuria (800 mg/day), likely representing light chain proteinuria. Thus, the contribution of the kappa light chain deposits to decreased eGFR can be questioned. At best, this patient may manifest “early” LCPT with subclinical features. Alternatively, the κ dominant staining in IF may merely be the consequence of the increased κ light chain production. Another interesting case was reported by Ryan et al.(214). In this patient proximal tubular epithelial crystalline deposits were initially overlooked on renal biopsy and thus the patient did not receive treatment for over a decade. Despite this, he had no progression of his renal disease. This case prompted a review of the literature to investigate how other patients with this rare renal disease had been treated, and what their outcomes were. The authors’ review of the literature showed that for many patients with κ-light chain crystalline proximal tubulopathy and a plasma cell dyscrasia, chemotherapy did not seem to drastically improve renal function and may not be necessary in the absence of multiple myeloma. Watchful waiting may, in fact, be more beneficial.

Although LCPT is typically characterized by the presence of crystalline deposits, we feel that there is sufficient data to support that non-crystalline forms of LCPT exist. The study of Vignon et al. included 39 patients with biopsy-proven LCPT (207). Based on LM, the non-crystalline variant was present in 15 patients. All patients had evidence of Fanconi syndrome (an inclusion criterium) and κ was the most prevalent light chain involved (in 14/15 cases). Obviously, crystals can be missed in LM, however the absence of crystals was in 9 patients confirmed by EM, which showed numerous and enlarged lysosomes (a somewhat non-specific finding). Kousios et al (2023) described 12 patients , of which 7 patients with non-crystalline LCPT (206). Again, there was predominant κ light chain restriction (in 5/6; one undetermined). The absence of crystals was confirmed by EM. Interestingly, in many patients EM showed intracytoplasmic, fibrillary substructures. Although the authors suggest that the patients often lacked features of Fanconi syndrome, there is no detailed information. Overall, Fanconi syndrome was incompletely tested or not documented in 4 patients. Normoglycemic glucosuria, hypophosphatemia and hypo-uricemia were present in three patients and absent in five. As discussed, it is unclear if all tubular functions were tested. Anyway, a normal serum phosphorus or normal uric acid may not exclude proximal tubular dysfunction in patients with reduced eGFR (average eGFR in the 7 patients was 36 ml/min/1.73m^2^, range 20-70 ml/min/1.73m2). Therefore, it is impossible to definitively exclude the presence of Fanconi syndrome. It is likely that the light chain contributed to kidney injury, since in patients who received hematologic treatment hematologic response was followed by renal response with improvement in eGFR.

We suggest that a diagnosis of LCPT can be made in patients who present with evidence of kidney injury, a measurable M-protein or abnormal FLC ratio, a kidney biopsy showing light chain restriction (κ preferred), and clinical features of Fanconi syndrome, also in the absence of crystalline structures.

Although some authors suggest that Fanconi syndrome is not required to diagnose LCPT, we caution to define LCPT merely based on light chain restricted staining in IF. We provide e detailed description of the literature to explain our cautionary note. Jung et al. described 5 patients with a presumptive diagnosis of LCPT. No patient had evidence of FS, as assessed by serum phosphorus, uric acid, and bicarbonate, and urine glucose. All patients had multiple myeloma. In two patients there were crystalline inclusions in the proximal tubule, which stained for κ light chain in both. These patients presented with kidney failure, with eGFR of 27 and 31 ml/min/1.73m^2^ respectively. As discussed this may have affected FS screening. In three patients there was λ light chain restriction. Crystals were absent, and EM studies showed “Increased/large/ dysmorphic lysosomes with mottled appearance”. Two of these patients had (near) normal eGFR, however both presented with severe proteinuria, with UPCR of 1.4 g/g and 4.2 g/g respectively, compatible with massive light chain proteinuria. IF findings may simply reflect the observed abnormal FLC ratio in the patients with multiple myeloma.

Larsen et al. searched their biopsy files for patients with evidence of light chain restricted staining of the proximal tubules and identified 13 cases (204). Of these, only 3 cases showed typical crystalline structures, which stained positive for κ in 2. Crystals were absent in 10 patients. In these patients LM showed some evidence of tubular injury (apical blebbing, cytoplasmic swelling, confirmed in EM by mitochondrial swelling and increased lysosomes with some mottled appearance). In all but one patient there was positive staining in IF for λ. These patients had presented with isolated proteinuria, or kidney failure. In five patients who were evaluated there was no evidence of Fanconi syndrome. All patients had increased free light chain concentrations in serum or urine, and 8 patients had multiple myeloma. Outcome data and information on treatment and response was lacking, therefore it remains unproven that the light chain deposits were responsible for the kidney injury.

Kousios et al. reviewed the literature, and collected 336 patients with suspected LCPT, in 286 supported by kidney biopsy (206). In 193 biopsies (69%) crystals were observed, whereas in 83 (31%) a non-crystalline variant was noted. Individual subject data were available of only 150 patients, 103 with crystalline LCPT and 47 with non-crystalline LCPT. There was no difference in reported prevalence of Fanconi syndrome (45/91 in the crystalline forms and 12/24 in the non-crystalline forms). However, this likely reflects lack of detailed data and incomplete assessment in many patients. There were clear differences in characteristics: patients with crystalline LCPT were more often kappa positive (96% vs 45%), had less often multiple myeloma (41% vs 62%) and less often nephrotic range proteinuria (35% vs 58%). Thus the patients with the non-crystalline, λ positive variant of LCPT had more often a hematologic malignancy, and excreted larger amounts of light chains. In their own cohort, Kousios et al. also noted lower proteinuria in crystalline LCPT (UPCR 83 mg/mmol in patients with the crystalline LCPT and 457 mg/mmol in the patients with non-crystalline LCPT). Based on a case report, Li et al. also concluded that non-crystalline LCPT in the absence of Fanconi syndrome is a rare entity (215). Their patient had a 10yr history of hypertension, and was known with stable CKD over a period of 2 years (serum creatinine between 110 and 120umol/l; UPCR 1-2 g/day). The patient also had a M-protein (IgGk) and an increased FLC ratio (5.7). Kidney biopsy was notable for proximal tubular κ restricted staining in routine IF, in the absence of crystals. EM showed swollen mitochondria and lysosomes, some with an irregular, mottled appearance. During a follow-up of 4 months FLC and eGFR have remained stable. In their review of the literature, they specifically focused on reports of LCPT without crystal formation. They summarized 40 cases (partly overlapping with the cases included in the abovementioned review by Kousios et al.). In 28 cases there was λ light chain restriction, Fanconi syndrome was reported in only 3 patients. Most patients had a hematologic disease (MM in 34), and only 3 fulfilled the criteria of MGRS.

In patients with the non-crystalline, λ positive variant of LCPT and absent Fanconi syndrome there is a very high incidence of multiple myeloma. These patients also have severe proteinuria, which in light of the often normal serum albumin levels reflects severe light chain proteinuria. We suggest that these patients may not represent typical LCPT, although the high load of FLC may be responsible for tubular cell damage. These cases may reflect what has been termed “light chain induced tubulopathy”. The association between light chains and tubular injury has been studied by Herrera. (216). These authors evaluated 5410 kidney biopsy reports and selected the biopsies showing light chain or heavy chain restriction (n=126; 2.5%). Of these 88 showed proximal tubular injury, and after exclusion of 31 cases with cast nephropathy, there remained 57 biopsies for evaluation (only 1.5% of all biopsies!). Based on histological and clinical criteria they discerned 4 subgroups: few patients showed proximal tubulopathy with cytoplasmic inclusion (mainly crystalline; n=4) or with lysosomal indigestion/constipation (n=3). In 6 there was κ restriction, and these patients showed glucosuria or phosphaturia, and would qualify for a diagnosis of LCPT. There were 22 patients with proximal tubulopathy without cytoplasmic inclusion (acute tubular necrosis variant; κ restriction in 16). These patients were not reported to have proximal tubule dysfunction. There were 28 patients with tubulopathy associated with an inflammatory reaction (acute tubular interstitial nephritis variant). In these patients there was linear staining of the TBM for the involved light chain, suggesting that these reflected LCDD. Almost all patients had evidence of light chain proteinuria. In this study outcomes were not reported. Again, the association between the presence of the light chain deposits and the kidney injury remained unproven.

In this respect, Stokes et al. already acknowledged that in some patients (with non-crystalline light chain restricted IF) this may represent mere physiological trafficking of light chains (205). In his patient, with increased λ light chains and λ light chain restriction, eGFR was normal and stable over a period of 10 months as was light chain proteinuria. Similar observations have been reported (213-215). The observation that most patients with non-crystalline lambda positive LCPT have an underlying MM and excrete massive amounts of light chains would be compatible with the notion that in (some) of these patients the IF restriction may merely be an epiphenomenon of the abnormal FLC ratio.

In conclusion: we feel that a diagnosis of LCPT is justified if there is PT injury, with crystals, light chain restriction, and at least some proof of (partial) Fanconi syndrome. In addition, there must be a hematologic disorder as signalled by an abnormal FLC or serum or urine M protein. Most of these patients with crystalline LCPT will be κ positive. Patients with PT injury, with evidence of Fanconi syndrome, and light chain restriction in IF staining of the proximal tubules, also can be diagnosed with LCPT, even in the absence of crystalline deposits. The diagnosis is strengthened by the presence of κ light chains and the finding in EM of fibrillary intracytoplasmatic deposits, often with dense amorphous material in lysosomes. A diagnosis of LCPT must be reconsidered in patients with no evidence of Fanconi syndrome, no crystals or organized deposits on EM, especially with λ light chain restriction. Clinically, this may not be very relevant, since the majority of patients with “suspected but unproven” LCPT will be diagnosed with an underlying hematologic malignancy (often MM), with abnormal FLC, and high urine light chain excretion. These patients will be treated according hematologic guidelines.

***Patients with suspected LCPT should be evaluated for the presence of proximal tubular dysfunction, characterized by aminoaciduria, normoglycemic glycosuria, renal tubular acidosis type II, hypo-phosphatemia and hypo-uricemia with increased fractional excretion of phosphate or uric acid.***

Often, the diagnosis of LCPT will be suggested by the renal pathologist after reviewing the kidney biopsy. It is notable that even in studies of patients with a “diagnosis” of LCPT not all patients have been evaluated for the presence of features of the Fanconi syndrome. We suggest that full evaluation should be done for the following reasons: 1. The presence of (incomplete or complete) Fanconi syndrome establishes in our view a diagnosis of LCPT. 2. The tubular abnormalities can be used to evaluate treatment response during follow-up (see treatment below). 3. The presence of tubular abnormalities will guide additional search for metabolic disorders. In the absence of features of Fanconi syndrome, the diagnosis or the clinical relevance of LCPT must be reconsidered (see above).

***We recommend that patients with LCPT and Fanconi syndrome should be evaluated for the presence of metabolic complications of the tubulopathy, which is mainly related to the severity of phosphate loss, and manifests as osteoporosis, non-traumatic fractures, and sometimes rickets.***

Patients with LCPT often present with features of proximal tubular dysfunction, one of the most important being severe hypo-phosphatemia and tubular acidosis. Patients with LCPT should be evaluated for the presence of metabolic complications, which are mainly related to the severity of acidosis and phosphate loss, and manifests as osteoporosis, non-traumatic fractures and sometimes rickets. In one study, bone pain and fatigue as symptoms related to the phosphaturia were seen 15/32 and 7/32 patients respectively (212). In another study, 40% of patients had stress-related bone fractures (207).

***Extrarenal light-chain deposition associated organ involvement is not seen in LCPT. Therefore, there is no need for untargeted evaluation of patients with LCPT to detect extrarenal manifestations.***

Since no light chain deposition associated extra-renal organ involvement has been described in patients with LCPT, routine evaluation for other organ manifestations is not necessary.

***In patients with LCPT, we recommend detailed hematologic evaluation including serum electrophoresis, and immunofixation, and serum FLC, followed by bone marrow aspirate with flow-cytometry and bone marrow biopsy.***

All patients with LCPT present with hematologic abnormalities (presence of M-protein in serum or urine; abnormal FLC) (206) All patients should receive bone marrow evaluation (see Chapter I). An overview of 6 series including 186 patients with “typical” LCPT reported multiple myeloma in 51 (27%) patients, M. Waldenström in 8 (4%),and SMM in 42 (23%) (206). The prevalence of MM is even higher in patients with “non-crystalline LCPT”. The absence of a measurable M-protein or urine FLC’s should lead to question the causality of the abnormal IF staining. In such patients we advise a re-evaluation while questioning the diagnosis of LCPT.

***In patients with LCPT, we recommend maximal conservative treatment as described for CKD. We recommend treatment of the metabolic complications guided by the observed abnormalities (vitamin D supplementation, correction of acidosis or hypophosphatemia).***

Although there is no evidence based on RCT’s we advise maximal conservative treatment for CKD according current guidelines. The metabolic complications should be treated targeting the observed abnormalities (vitamin D supplementation, correction of acidosis or hypophosphatemia). The prognosis and morbidity of patients with LCPT not only depends on kidney dysfunction, which usually is quite slowly progressive, but also on the metabolic complications secondary to the tubular dysfunction(207) (205) (212).

***The decision to start hematologic treatment should be based on individual patient characteristics. We recommend clone-targeted treatment in patients with progressive kidney failure, or patients with severe and untreatable metabolic complications. We suggest that changes in FLC are useful to monitor and guide treatment.***

Literature on the treatment of patients with LCPT as subtype of MGRS is biased, since most studies included patients with a hematologic malignancy. Overall, the data indicate that clone targeted treatment, resulting in a hematologic response led to correction of metabolic complications and stabilization of renal function. However, treatment does not necessarily improve eGFR, and eGFR at baseline proved an important independent predictor of worse outcome. These literature data cannot be generalized to patients with LCPT as subtype of MGRS. In many patients with LCPT kidney injury is slowly progressive. Although ESRD developed in 4 of 8 untreated patients reported by Ma et al. , the median time to ESRD was 196 months (16.3 yr) (212). Therefore, treatment should be individualized, taking into account age, expected survival, comorbidity, and predicted eGFR loss. Especially patients with progressive kidney failure or patients with severe metabolic complications which cannot be controlled by the usual supplementation will benefit most from hematologic treatment. The observations in patients with suspected LCPT with crystalline deposits, but absent Fanconi syndrome are most relevant. Many patients had stable disease over periods of 10 months to 10 years (205, 213-215). The most striking observation is the patient described by Ryan et al.(214). This patient was known with “MGUS”, with M-protein detected in serum and urine. Serum creatinine was 2mg/dl, and proteinuria 3.6g/day, without nephrotic syndrome. A kidney biopsy was done and a diagnosis of arteriolar nephrosclerosis was made. After 10 years, a repeated kidney biopsy was done because of persistent CKD, with serum creatinine 1.7 mg/dl and proteinuria 2.9 g/day. Additional assessment disclosed glucosuria, and his serum phosphate was low-normal. The urine contained approximately 1000 mg of free kappa chains, with serum kappa 1180 mg/l and serum lambda 11.5 mg/l (FLC ratio 100). There was no evidence of multiple myeloma (PC 2%). Kidney biopsy showed crystalline, kappa restricted deposits in the proximal tubular cells compatible with LCPT. Revision of the first kidney biopsy by EM confirmed the presence of crystals. During follow-up without hematologic therapy, serum creatinine and proteinuria have remained stable, although patient developed frank hypophosphatemia (serum phosphate 1.9 mg/dl (0.61 mmol/l) .

Four recent cohort studies illustrate the characteristics of patients with LCPT and report outcomes (Table 8.1)(210) (205-207). Vignon *et al.* described 49 patients, with mean eGFR of 33 ml/min/1.73m^2^ (207). Only 3 patients had eGFR >60 ml/min/1.73 m^2^. Although light chain was present in the urine in all patients, and all patients with available data had abnormal FLC ratio, most patients (80%) were defined as MGRS (including 25 patients with SMM). Median follow-up was 3.8 years. Seven patients (with MGRS) did not receive any specific therapy. These patients were followed for a median of 7.5 yr. Four progressed to ESKD (median time to ESKD 15 yrs) whereas in three eGFR remained stable (follow-up 3 yrs). Although clinical details of these 7 patients are not provided, the data show that renal progression rate can be slow. Forty-two patients received chemotherapy, mainly because of progressive kidney disease. Overall 38 patients had a hematologic response. In these patients eGFR stabilized. An improvement in proximal tubular function was observed in 13 patients. Stabilization of eGFR was observed in all patients with hematologic response and a reduction of FLC, independently of the specific treatment modality. During follow-up renal function remained stable, without maintenance therapy, as long as the decrease of serum FLC persisted. Estimated GFR improved by 14% in patients with CR/VGPR, decreased by 19% in patients with PR, and decreased by 36-40% in treatment naïve or treatment resistant patients. Unfortunately, this percentage change is the change from baseline to last observation, and it is not clear if follow-up time was equal. In fact, untreated patients were more often diagnosed before 2000. Thus Δ eGFR even in untreated patients may be not higher than 5-10 % per year. Tubular response was limited and occurred in 7/14 patients treated with HDM-ASCT as compared to 5/24 patients treated with chemotherapy. Tubular response was associated with deeper hematologic response, which occurred in 8/14 patients treated with HDM-ASCT and in 6/24 patients treated with chemotherapy. Of note, Bortezomib was used in only 11/24 patients, and renal response was similar in HDM-ASCT vs Bortezomib treated patients.

Stokes *et al.* described 46 patients with LCPT (205). Although proteinuria was > 3 g in 37% of patients, serum albumin was normal, which suggests that the urine protein was mainly composed of light chains and tubular proteins. Thirty patients were evaluated for treatment response. In this study, 10 patients were treated with high dose chemotherapy + ASCT, 12 patients received chemotherapy only, and 8 patients were not treated. Patients treated with ASCT were younger (51 vs 66 yrs) and had better kidney function, indicating treatment bias. Notably, hematologic response rate was low, and occurred in only 25-30% of patients. No treated patient developed ESRD. Overall eGFR increased by 10 ml/min/1.73m^2^ in patients treated with ASCT, and decreased by 7 ml/min/1.73m^2^ in untreated patients. These data must be interpreted with caution, in fact, eGFR decreased from 35 to 28 ml/min/1.73m^2^ in the untreated patients over a period of almost 5 years of follow-up i.e. a decrease of 1 ml/min/yr which is not different from the average age-dependent eGFR decrease.

Kousios *et al.* included 12 patients with light chain proximal tubulopathy from a single center (206). Only a minority of patients had crystalline inclusions (n=5; 42%). Nine patients underwent treatment after diagnosis. Four patients received ASCT after melphalan treatment, another four patients were treated with bortezomib and one patient was treated with dexamethasone, rituximab and cyclophosphamide. In all treated patients, at least a partial hematologic response was observed. In all but one of these patients, the eGFR remained stable during follow-up or increased. Data on follow-up of proteinuria was incomplete. Two untreated patients showed continued loss of renal function.

The study of Lin et al included 47 patients (210). Mean eGFR was 55 (SD19) ml/min/1.73m^2^. Although nephrotic range proteinuria was present in 26% of patients, hypo-albuminemia was noted in only 1 patient, again indicating that urine protein was likely composed of light chains. Outcome could be evaluated in 37 patients with mean follow-up of 18 months. Most patients were treated with chemotherapy, in 11 with added ASCT. Hematologic response rates were Cr 7%, VGPR 30%, PR 26% and NR 37% respectively. eGFR improved in only 9% of patients, remained stable in 79%, and progressed in 12%. This data illustrates the slow progression rate in LCPT, with stablisation of eGFR in many patients with limited hematologic response. In the untreated patients there was slow progressive decrease of eGFR over a period of 4-7 years.

We do not discuss in detail two other Chinese studies, since many patients were not biopsied, and biopsy reports did not include information of IF or EM . Also in these studies renal response followed hematologic response, with tubular response being more frequent than eGFR response. Again, in untreated patients the decrease in eGFR was slow, and ranged from 0-20% over a period of 37 months (209), or 10-15 ml/min/1.73m^2^ over 36 months (208)

The data in literature underline the importance of individualized therapy in patients with MGRS-LCPT. Treatment is advised in patients with decreasing eGFR and/or severe metabolic complications of the tubulopathy. A conservative approach can be considered in patients with mild kidney injury, especially in patients aged > 75 years or patients with a limited life expectancy, taking into account the abovementioned eGFR decrease of 1-5 ml/min/yr.

Light chain crystalline podocytopathy

***A diagnosis of LCCP requires a kidney biopsy, showing crystals in the podocyte that are composed of a monoclonal light chain. Since crystals are not always visible in LM, and immunofluorescence on frozen tissue is often falsely negative a diagnosis of LCCP requires detailed examination of the kidney biopsy with EM and immunofluorescence on paraffin embedded tissue after pronase digestion.***

LCCP is a new and only very recently described disease entity. Nasr et al. reported 25 patients, observed over a period of 20 years in 4 large expert centers (217). Although the precise prevalence thus is unknown, it is an ultrarare condition. Median age was 56 years, with male predominance (68% male). Most patients presented with proteinuria, and a kidney biopsy showed crystalline deposits in the podocytes, with light chain restricted staining, predominantly κ (87%). Importantly, crystals were not always observed in routine light microscopy (72%), and light chain staining was positive using routine IF-F in only 12%. Thus examination by EM, and use of paraffin IF after pronase digestion is necessary. Interestingly, in 22% of patients, crystalline deposits were present in the eye (keratopathy). Of note, thus far non-crystalline forms of LCCP have not been described.

***Patients with LCCP should be evaluated for the presence of LCPT.***

In 80% of patients a concomitant diagnosis of LCPT was made, although only 10% of patients had documented Fanconi syndrome. Since in many patients, evaluation for Fanconi syndrome was not done, the true prevalence of LCPT is unknown.

***Diagnosis, evaluation, and treatment of patients with LCCP should be done as described for LCPT.***

Since LCCP is ultrarare, we suggest that patients with a suspected diagnosis of LCCP are referred to expert centers, and preferably entered in disease registries. The underlying disease was MGRS in 55% of patients and multiple myeloma in 45%. Since most patients present with moderate to severe proteinuria, and evidence of podocyte injury, we suggest that these patients receive conservative therapy as advised for patients with proteinuric CKD. Outcome data and/or treatment data was available for 23 patients. 21 patients underwent plasma-cell directed therapies, six patients were treated in addition with autologous stem cell transplantation. 12 out of 20 patients had a complete or a very good partial hematologic response to therapy, whereas 4 had a partial response and 4 were considered non-responder. Renal outcome was available for 18 of these patients. Patients with at least a very good partial response to treatment showed in eight out of 10 cases a renal response whereas patients with no or only a partial response showed a worse outcome; a renal response was reported in only one out of eight cases. Six patients developed ESKD. Interestingly, all of these patients had FSGS in light microscopy.

Table 8.1 overview of studies in patients with LCPT: clinical characteristics of patients

| Author/yr | Vignon 2017  (207) | Stokes 2016  (205) | Lin 2024  (210) | Kousios 2023(206) |
| --- | --- | --- | --- | --- |
| Country/period | France/USA  1988-2014 | USA  2000-2014 | China  2007-2023 | UK  2005-2020 |
| N | 49 | 46 | 47 | 12 |
| Age (yr) | 58 (37-83) | 60 (39-87) | 57 (32-77) | 70 (47-80) |
| Gender M/F | 30/19 | 29/17 | 30/17 | 9/3 |
| Screat (umol/l)  Or  eGFR (ml/min/1.73m^2^) | 171 (70-1278) | 177  (83% renal insufficiency) | 116 (45-442) | 43 (20-78) |
| UPCR (g/day)  Or # g/10mmol | 1.5 (0.5-10) | 2.5  (87% UPCR > 1 g/day)  Normal S albumin | 2 (0.3-10.3) | 3.3 (0-8.2) (50% with proteinuria in nephrotic range) |
| Tubulopathy  Aminoaciduria  Glucosuria  Increase FEph  Hypophosphatemia  Increase FE urate  Hypouricemia  Acidosis  Fracture (stress) | 95%  80%  90%  70%  100%  70%  NA  40% | (N=17 tested)  12%  88%  NA  29%  NA  29%  41%  NA  27 apparently no Fanconi, however unclear which tests were done | NA  84%  34%  47%  NA  56%  NA  Bone pain in 22%  Increased urine RBP in 83% | 3 confirmed  NA  3  NA  3  NA  3  NA  NA  FS incompletely tested/documented in all patients, 5 patients had no glucosuria, and normal phosphate and uric acid (see text) |
| Monoclonal Ig  SPEP/sIFE  Light chain only  К  λ | 70%  30%  95%  5% | 96%  4% | 42% | 8 (IgG5, IgM2, IgA1)  4 (all kappa) |
| Hematologic disease  MGRS  SMM^$$^  MM  Other | 13 (27%)  25 (51%)  7 (14%)  4 (8%) | 21 (45%)  7 (15%)  15 (33%)  3 (7%) | 38 (81%)  9 (19%) | 5 (42%)  7 (58%) |
| Histology  Crystalline  Non-crystalline (increase vacuoles, phagolysosomes) | 24 (only 17 in LM)  15 | 40 (only 35 in LM)  6 | 44*  3 | 5  7 |
| Light chain involved  Kappa  lambda | 37  2 | 44  2 | 41  6 | 11  1 |

$$ SMM should be considered MGRS. * in 8 patients a mixed pattern was present, with crystalline inclusions and amorphous deposits

FE = fractional excretion. Ph = phosphorus

**Chapter 9: Monoclonal Immunoglobulin associated C3-glomerulopathies (C3G)**

**Introduction**

C3G are kidney diseases attributed to alternative complement pathway dysregulation and characterized by dominant deposition of C3. It includes C3 glomerulonephritis (C3GN) and Dense Deposit Disease (DDD). The presence of a monoclonal immunoglobulin in the serum defines monoclonal immunoglobulin associated C3G. Presumably, the monoclonal immunoglobulin interferes with the regulation of the complement system which then drives the development of C3G.

**Summary statements**

***Diagnosis, evaluation and management of patients with C3G should be done in (consultation with) expert centers.***

***A diagnosis of C3G requires a kidney biopsy, showing dominant staining for C3 by IF.***

***We recommend EM studies to differentiate between Dense Deposit Disease (DDD) and glomerulonephritis with isolated C3 deposits (C3GN).***

***In patients with C3G perform a detailed evaluation of the complement system and search for a monoclonal immunoglobulin in serum, with the sequence of evaluation determined by age.***

***We suggest that a diagnosis of monoclonal immunoglobulin associated C3G be made in patients with C3G who are aged >50 yr with a detectable monoclonal immunoglobulin in serum.***

***Extrarenal organ involvement is rarely seen in C3G. Therefore, there is no need for routine evaluation of patients with C3G to detect extrarenal manifestations.***

***In patients with C3G and a documented monoclonal immunoglobulin in serum, we recommend hematologic evaluation including bone marrow aspirate with flow-cytometry and bone marrow biopsy.***

***In patients with C3G, we recommend maximal conservative treatment for CKD.***

***In patients with C3G treatment decisions should take into account patient characteristics and severity of kidney injury.***

***We recommend clone targeted treatment in selected patients with C3G and documented bone marrow abnormalities. We suggest empirical therapy in patients with suspected MIg-associated C3G and no detectable clone, with treatment initiation dependent on comorbidity, age and severity of kidney injury.***

**Rationale**

***Diagnosis, evaluation and management of patients with C3G should be done in (consultation with) expert centers.***

The C3 glomerulopathies (C3G) are a recently defined subgroup of rare glomerular diseases. Diagnosis and management requires expertise in nephropathology, laboratory medicine, including complement assays, and genetics. Patients will benefit from consultation with expert centers.

***A diagnosis of C3G requires a kidney biopsy, showing dominant staining for C3 by IF. We recommend EM studies to differentiate between Dense Deposit Disease (DDD) and glomerulonephritis with isolated C3 deposits (C3GN).***

C3G is defined by dominant staining for C3 in the glomeruli by IF, with intensity at least 2+ higher than the staining for IgG or other immunoglobulins. Based on the findings on electron microscopy, C3 glomerulopathies are subdivided in C3GN and Dense Deposit Disease (DDD).

***In patients with C3G perform a detailed evaluation of the complement system and search for a monoclonal immunoglobulin in serum, with the sequence of evaluation determined by age.***

C3G is the consequence of complement dysregulation. This can be caused by inherited or acquired abnormalities in the complement system (e.g. mutations in genes that encode complement regulatory proteins, auto-antibodies that activate the C3 convertase or prevent its inhibition). Genetic and auto-immune C3G are typically observed in children and young adults. There is an association between the presence of a monoclonal immunoglobulin and C3G. Based on experimental studies, it is likely that the monoclonal immunoglobulin interferes with normal complement regulation. Monoclonal immunoglobulin-associated C3G is considered a subtype of MGRS (MGRS-C3G), and typically but not exclusively presents at age >50 yr.

***We suggest that a diagnosis of MGRS be made in patients with C3G who are aged >50 yr with a detectable monoclonal immunoglobulin in serum.***

In a patient who presents with C3G at age > 50 yr, a diagnosis of MGRS is highly likely if a monoclonal immunoglobulin is present in serum. In routine clinical practice it is impossible to prove that the monoclonal immunoglobulin interferes with the complement cascade. Therefore, the diagnosis is made by exclusion. In younger patients, inherited and acquired defects in the complement system are the most likely cause, and should be ruled out. In elderly patients, the presence of a monoclonal immunoglobulin is considered sufficient to make a diagnosis of MGRS-C3G.

***Extra-renal organ involvement is rarely seen in C3G. Therefore, there is no need for a routine evaluation of patients with C3G to detect extrarenal manifestations.***

C3G is usually a renal limited disease, although a recent study suggests that digital ischemia can develop as rare extra-renal manifestation of C3G. Routine evaluation is not needed to search for subclinical extrarenal disease manifestations.

***In patients with C3G and a documented monoclonal immunoglobulin we recommend hematologic evaluation including bone marrow aspirate with flow-cytometry, and bone marrow biopsy.***

The presence of a monoclonal immunoglobulin in serum is essential to make a diagnosis of MGRS-C3G. Bone marrow examination including flow cytometry is necessary to identify a clone. A minority of patients with MGRS-C3G is diagnosed with an underlying hematologic malignancy,

***In patients with C3G we recommend maximal conservative treatment of CKD.***

Patients with C3G present with kidney injury. Although not specifically evaluated in this population, all patients should be treated according the guidelines for CKD.

***In patients with C3G treatment decisions should take into account patient characteristics and severity of kidney injury.***

C3G is usually a disease limited to the kidney. A wait and see policy may be justified in patients with old age and slow progression of renal disease, taking into account life expectancy, comorbidity, and treatment associated toxicity.

***We recommend clone targeted treatment in selected patients with C3G and documented bone marrow abnormalities. We suggest empirical therapy in patients with suspected MGRS-C3G and no detectable bone marrow clone, with treatment initiation dependent on comorbidity, age and severity of kidney injury.***

In selected patients outcome is improved by aggressive hematologic therapy. Clone directed therapy is (somewhat) more effective than standard immunosuppressive therapy. Treatment efficacy can be measured by monitoring the hematologic response. Hematologic response is linked to kidney response and outcome.

**Supportive evidence**

***Diagnosis, evaluation and management of patients with C3G should be done in (consultation with) expert centers.***

The C3 glomerulopathies (C3G) are a recently defined subgroup of rare glomerular diseases (218). C3G is attributed to complement dysregulation (219). In children and young adults C3G is caused by inherited or acquired abnormalities in the complement system. In older aged patients (>50 years) C3G is most likely caused by a monoclonal immunoglobulin, an association described first in 2011 (220). All forms of C3G are rare, C3G associated with a monoclonal immunoglobulin is least prevalent. A Spanish study evaluated 155 patients with C3G diagnosed in 35 centers in the period between 1995 and 2020 (221). In 23 patients an underlying monoclonal gammopathy was detected (15%). Since C3G are rare diseases, and the differential diagnosis requires expertise it is advised to consult centers with experts in nephropathology and complement diagnostics.

***A diagnosis of C3G requires a kidney biopsy, showing dominant staining for C3 by IF.***

***We recommend EM studies to differentiate between Dense Deposit Disease (DDD) and glomerulonephritis with isolated C3 deposits (C3GN).***

C3G is defined by dominant glomerular staining for C3 by IF, with intensity at least 2+ higher than the staining for IgG or other immunoglobulins. On light microscopy, the findings are variable and can range from minimal glomerular changes to proliferative patterns, including mesangial proliferative, endocapillary proliferative, and membranoproliferative patterns of injury.(221-223) Crescents can also be observed. Based on the findings on electron microscopy, C3 glomerulopathies are subdivided in C3GN and in Dense Deposit Disease (DDD). DDD is characterized by the presence of highly electron-dense deposits that thicken the lamina densa of the glomerular basement membrane, with a ribbon or a sausage-like appearance. Similar deposits can be observed in Bowman’s capsule and the TBM. In C3GN, electron deposits of intermediate density are present in the mesangium, in a subendothelial location and, sometimes, in a subepithelial location. Hump-like deposits can be observed in both DDD and C3GN. The mere presence of C3 dominant mesangial of capillary wall deposits is not sufficient to diagnose C3G. A similar pattern can be observed in post-infectious glomerulonephritis.

***In patients with C3G perform detailed evaluation of the complement system and search for a monoclonal immunoglobulin in serum, with the sequence of evaluation determined by age.***

C3G is attributed to complement dysregulation (219). In 2011, an association between the presence of a monoclonal immunoglobulin and C3G was suggested (220). Thus it is now evident that C3G can be caused by inherited abnormalities in the complement system (e.g. mutations in genes that encode complement regulatory proteins) or by acquired abnormalities, the latter including polyclonal auto-antibodies and monoclonal immunoglobulins that activate the C3 convertase or prevent its inhibition. Already in 1992 it was shown that a monoclonal λ chain could activate the alternative complement pathway (224). In a follow-up study, the λ dimer acted as a “mini-autoantibody” against complement factor H (225). More recently, Li et al. presented a 76 yr old patient with C3G, who presented with nephrotic syndrome, hematuria, progressive kidney insufficiency, low C3, and a monoclonal immunoglobulin in serum (226). They subsequently demonstrated that the monoclonal IgG3λ acted as an antibody against factor H, and was responsible for increased C3-convertase activity. Altogether, these reports clearly demonstrate that monoclonal proteins can interfere with normal complement regulation.

Not unexpected, in patients with a C3G there is a bimodal age-distribution: C3G associated with inherited or acquired (auto-immune) defects in complement regulation is typically diagnosed at young age, whereas C3G associated with a monoclonal immunoglobulin is diagnosed at older age. In one series, median age of patients with C3G without a detectable monoclonal immunoglobulin was 28 yr (range 4-84) as compared to 60 yr (20 – 85) years in patients with MGRS-C3G (223). The prevalence of a MIg as cause of C3G increases with age: a monoclonal immunoglobulin was detected in 36% of patients with C3G aged 50-59 yr, in 59% of patients aged 60-69 yr, and in 94% of patients aged 70-79 yr (222). A thorough evaluation is needed in all patients with C3G. Although there is a bimodal age distribution, there is considerable overlap. Genetic studies may be reserved for the children and adolescents, in contrast an M-protein assay is always required in adults. Unfortunately, the presence of C3-nephritic factor activity, or of anti-factor H antibodies does not differentiate, since in clinical practice it cannot be determined if these complement abnormalities are caused by the polyclonal or monoclonal immunoglobulin compartment.

***We suggest that a diagnosis of monoclonal associated C3G be made in patients with C3G aged >50 yr with a detectable monoclonal immunoglobulin in serum.***

Although experimental studies have proven that MIg can interfere with normal complement regulation, in clinical practice it is impossible to proof that the detected monoclonal immunoglobulin is causing complement dysregulation. Therefore, in patients with biopsy proven C3G a diagnosis of monoclonal immunoglobulin-associated C3G is based on circumstantial evidence. In adolescents and young adults C3G is likely related to genetic or auto-immune dysregulation of the complement pathway. Unfortunately, genetic mutations are found in only 15-20% of patients. In the majority of patients with C3G abnormalities in the complement pathway can be observed (low C3, presence of C3 nephritic factor, antibodies against factor H). Importantly, these *in vitro* tests (using patient serum) cannot discriminate the origin of complement dysregulation: auto-immune *versus* monoclonal immunoglobulin associated C3G. In theory, selective adsorption of the monoclonal Ig might allow performing complement tests in the absence or presence of the monoclonal immunoglobulin. However, this is not feasible in clinical practice. Therefore, in patients with C3G and a documented monoclonal immunoglobulin a presumptive diagnosis of MIg-associated C3G can be made. When in doubt, e.g in patients with C3G presenting at relative young age (30-50 years), who present with barely detectable M-protein without bone marrow abnormalities, consultation of experimental laboratories should be considered.

***Extrarenal organ involvement is rarely seen in C3G. Therefore, there is no need for routine evaluation of patients with C3G to detect extrarenal manifestations.***

C3G are usually diseases limited to the kidney, therefore there is no need to routinely search for heart, liver, or neurological damage or abnormalities of other organs. An association between C3G and digital ischemia/ulcers has been described (227). In this case series, 5 patients with C3G were presented with evidence of severe digital ischemia, manifesting as Raynaud syndrome and/or digital necrosis. Notably, in only one patient there was concomitant presentation of the C3G and the digital ischemia, in the other patients C3G either preceded or followed the digital ischemia by many years. All patients had a MIg, C3 levels were slight below normal in three. Cryoglobulins were absent.

***In patients with C3G and a documented monoclonal immunoglobulin, we recommend hematologic evaluation including bone marrow aspirate with flow-cytometry, and bone marrow biopsy.***

The presence of a monoclonal immunoglobulin in serum defines MIg-associated C3G. hematologic evaluation is required including bone marrow biopsy and flow cytometry, to assess the presence of a bone marrow clone. Few patients with MIg-associated C3G have an underlying hematologic malignancy, thus the majority of patients (60-90%) can be classified as MGRS.

***In patients with C3G, we recommend maximal conservative treatment for CKD.***

Patients with C3G present with CKD. Although there is no evidence specifically for patients with C3G, we suggest that all patients should receive treatment as recommended for CKD.

***In patients with C3G treatment decisions should take into account patient characteristics and severity of kidney injury.***

The clinical course of patients with C3G is variable, and many patients will be elderly and have associated comorbidity. Not every patient will benefit from aggressive therapy.

***We recommend clone targeted treatment in selected patients with C3G and documented bone marrow abnormalities. We suggest empirical therapy in patients with suspected MIg-associated C3G and no detectable clone, with treatment initiation dependent on comorbidity, age and severity of kidney injury.***

There is limited data on Mig-associated C3G. Information is mostly limited to case reports, reviewed by Li et al (226), which cannot guide decision making. Three recent studies, that included 23 – 50 patients are summarized in Tables 9.1 and 9.2 and discussed below.

Chauvet *et al.* reported 50 patients with monoclonal immunoglobulin associated C3G (in the study period there were 141 patients with C3G and no identified M-protein) (222). In 35% of patients the monoclonal immunoglobulin was already present long before the onset of kidney disease (interval 40 (12-120) months). The prevalence of MIg-associated C3G increased with age, from 36% in patients aged 50-59 yr, 59% in patients aged 60-69yr, and 94% in patients aged >70 yr. EM studies were done in 25 patients, and DDD was diagnosed in only one. A hematological malignancy (requiring treatment) was diagnosed in 20 patients. Treatment was variable: 13 patients received conservative therapy only, 8 patients received immunosuppressive therapy, and 29 patients received chemotherapy (mostly bortezomib based) adapted to the identified bone marrow clone. Decisions toward treatment were somewhat biased; clone-directed therapy was more often used in patients diagnosed with SMM, MM, or CLL (17/20) than in patients diagnosed with MGRS (12/30). There were no major differences in clinical characteristics. Follow-up was 24 months. Overall, hematologic response was observed in 18 of 37 treated patients, followed by a renal response in 15 (83%). In contrast, a renal response was observed in only 5 of 19 patients without hematologic response (28%). In multivariable analysis, the hematologic response was the only determinant of renal response. There was a clear benefit of clone directed chemotherapy: more patients achieved hematologic response and renal response. Complete or partial renal remission rate was 74% in chemotherapy treated patients compared to 5% in the other patients. Of note, C3 levels normalized in patients with a hematologic response. Interestingly, some patients had a renal response without clear hematologic response. Also, the comparison group (not clone directed therapy) consisted mostly of patients either left untreated or treated with steroids. In fact, in this group only 2 patients received therapy which could be considered effective i.e either Cyclophosphamide or Rituximab.

Ravindran *et al.* included 36 patients with C3G and a monoclonal immunoglobulin, identified from a cohort of 114 patients with C3G (223). A diagnosis of DDD was made in only 4 (11%) patients. The majority of patients had MGRS (n=28, including 2 with SMM). An underlying hematologic disease was present in the other ( 5 MM, 1 CLL, 1 MALT lymphoma, 1 Cryglobulinemia type I). During follow-up 2 patients with MGRS progressed to MM (and thus treated accordingly with targeted therapy). Clinical characteristics are given in table 9.1. Outcome was evaluated after a median follow-up of 44 months. Nine patients (25%) with C3G and MIg progressed to ESKD (as compared to 12% in patients with C3G without MIg). There were 33 patients who received some form of immunosuppressive/hematologic therapy. Seventeen patients with C3G and MIg, all diagnosed with MGRS, received non-targeted therapy. In the majority this consisted of steroids with or without MMF. Of 14 evaluable patients, 7 achieved a complete or partial renal response. Bias cannot be excluded since some of these patients presented with mild kidney injury. Sixteen patients (including 7 patients with MGRS received ”targeted” treatment). In this subgroup, 4 patients developed complete and 2 developed partial response. There was no difference in renal survival between patients who received targeted vs untargeted therapy. Specifically, renal response rate in patients with MGRS was also not different (complete or partial renal response in 2/7 patients treated with targeted therapy). Of note, in the group of 16 patients treated with targeted therapy, there was an association between hematologic and renal response.
[truncated: 75,742 more chars]
